# New Sulfanilamide Derivatives Incorporating Heterocyclic Carboxamide Moieties as Carbonic Anhydrase Inhibitors

**DOI:** 10.3390/ph14080828

**Published:** 2021-08-23

**Authors:** Andrea Angeli, Victor Kartsev, Anthi Petrou, Mariana Pinteala, Roman M. Vydzhak, Svitlana Y. Panchishin, Volodymyr Brovarets, Viviana De Luca, Clemente Capasso, Athina Geronikaki, Claudiu T. Supuran

**Affiliations:** 1 Department of Chemistry “Ugo Schiff”, University of Florence, Via della Lastruccia 3-13, 50019 Sesto Fiorentino, Italy; claudiu.supuran@unifi.it; 2 Centre of Advanced Research in Bionanoconjugates and Biopolymers Department, “Petru Poni” Institute of Macromolecular Chemistry, 707410 Iasi, Romania; pinteala@icmpp.ro; 3 InterBioScreen, Chernogolovka, 142432 Chernogolovka, Moscow Region, Russia; vkartsev@ibscreen.chg.ru; 4 Department of Pharmacy, School of Health, Aristotle University of Thessaloniki, 54124 Thessaloniki, Greece; anthi.petrou.thessaloniki1@gmail.com; 5 Department of Chemistry of Bioactive Nitrogen-Containing Heterocyclic Bases, V.P. Kukhar Institute of Bioorganic Chemistry and Petrochemistry, NAS of Ukraine 1, Murmanska St, 02094 Kyiv, Ukraine; rmvydzhak@gmail.com (R.M.V.); svpanch@ukr.net (S.Y.P.); brovarets@bpci.kiev.ua (V.B.); 6 Institute of Biosciences and Bioresources, CNR, Via Pietro Castellino 111, 80131 Napoli, Italy; viviana.deluca@ibbr.cnr.it (V.D.L.); clemente.capasso@ibbr.cnr.it (C.C.)

**Keywords:** carbonic anhydrase, metalloenzymes, inhibitors, molecular docking

## Abstract

Carbonic Anhydrases (CAs) are ubiquitous metalloenzymes involved in several disease conditions. There are 15 human CA (hCA) isoforms and their high homology represents a challenge for the discovery of potential drugs devoid of off-target side effects. For this reason, many synthetic and pharmacologic research efforts are underway to achieve the full pharmacological potential of CA modulators of activity. We report here a novel series of sulfanilamide derivatives containing heterocyclic carboxamide moieties which were evaluated as CA inhibitors against the physiological relevant isoforms hCA I, II, IX, and XII. Some of them showed selectivity toward isoform hCA II and hCA XII. Molecular docking was performed for some of these compounds on isoforms hCA II and XII to understand the possible interaction with the active site amino acid residues, which rationalized the reported inhibitory activity.

## 1. Introduction

**Figure 1:**
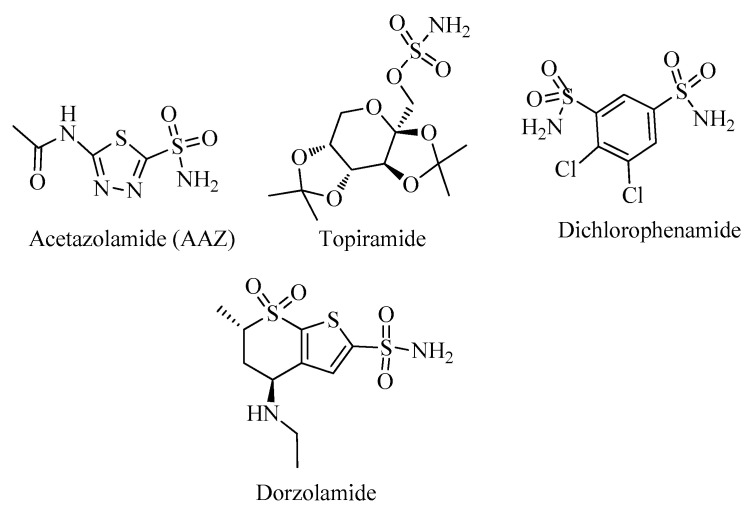
Known CA inhibitors.

Carbonic anhydrases (CAs) are a ubiquitous metalloenzyme family present in both eukaryote and prokaryote organisms [1]. To date, were discovered eight evolutionarily unrelated gene classes encoded as α-, β-, γ-, δ-, ζ-, η-, θ-, ι-CAs [2,3,4,5,6,7,8]. All these enzymes promoting the hydration of carbon dioxide (CO_2_) to bicarbonate (HCO_3_^−^) and protons (H^+^) following a two-step catalytic mechanism [9]. In humans, all CAs belong to the α-class with fifteen isoforms differing by molecular features, oligomeric arrangement, cellular localization, distribution in organs and tissues, expression levels, kinetic properties, and response to different classes of inhibitors [10,11,12]. To date, twelve catalytic active isoforms (CA I−IV, VA−VB, VI−VII, IX, and XII−XIV) are known, with a variable CO_2_ hydrase activity, which play pivotal roles in a variety of physiological processes connected to pH and CO_2_ homeostasis, respiration, electrolyte secretion, biosynthetic reactions (i.e., gluconeogenesis, lipogenesis, and ureagenesis), bone resorption; calcification; and tumorigenicity [13]. On the other hand, abnormal levels or activities of these enzymes have been often associated with different human diseases, some of which have been clinically exploited and validated as therapeutic targets for the treatment or prevention of various pathologies such as glaucoma, edema, neurological disorders, epilepsy and more recently cancer [14,15,16]. In this context, many efforts are made to explore novel and selective Carbonic Anhydrase Inhibitors (CAIs) leading compounds with potential biomedical applications [17,18]. Some of the CA inhibitors mentioned in CheMBL are presented in Figure 1.

In addition, furano- and thienopyroles have attracted interest of medicinal chemists due to their wide range of biological activities, such as antiviral [19,20,21], antimicrobial [22,23,24], anticancer [25,26,27], anti-inflammatory [28,29], antidiabetic [30], carbonic anhydrase inhibitory [31]. On the other hand, some sulfonamides also show anticancer [32,33], antimicrobial [34,35], anti-inflammatory [36,37] antidiabetic [38], and antitubercular [39] activities.

Taking into account all of the mentioned above, here we report the synthesis of *N*-(4-sulfamoylphenyl)-4*H*-thieno[3,2-*b*]pyrrole-5-carboxamide and *N*-(4-sulfamoylphenyl)-4*H*-furano[3,2-*b*]pyrrole-5-carboxamide derivatives incorporating into one scaffold carboximide and sulfonamide moieties. Furthermore, 6-oxo[1,3]thiazolo[3,2-*b*][1,2,4]triazole derivatives were also synthesized. The experimental studies were developed together with in silico techniques, aimed to propose a reliable binding disposition for this class of CAIs.

## 2. Results and Discussion

### 2.1. Design and Synthesis of Compounds

**Scheme 1:**
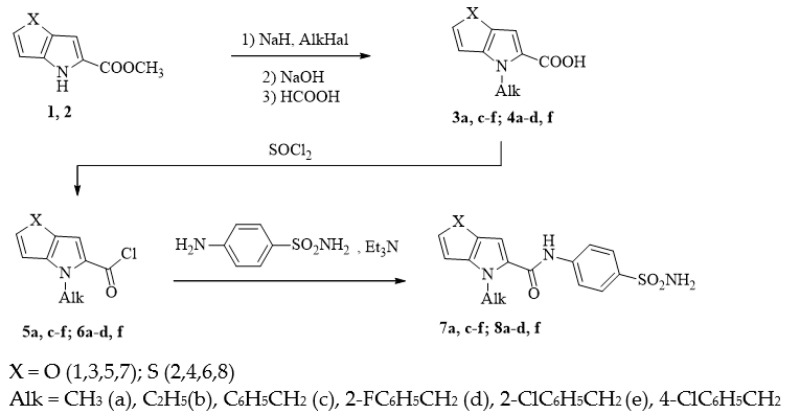
Synthesis of furo[3,2-*b*]pyrrole and thieno[3,2-*b*]pyrrole derivatives.

The synthesis of target sulfonamides **7a,c-f** and **8a-d,f** is presented in Scheme 1. Intermediate esters were obtained by alkylation of the starting compounds **1** [40] or **2** [41] with the appropriate alkyl halides in DMF using sodium hydride as a base. Then, these compounds were used without additional purification to prepare 4-alkyl-4*H*-furo[3,2-*b*]pyrrole-5-carboxylic acids **3a,c-f** and 4-alkyl-4*H*-thieno[3,2-*b*]pyrrole-5-carboxylic acids **4a-d,f**. Acyl chlorides **5** and **6** were obtained (with yields of 50–80%) by the treatment of carboxylic acids **3** and **4** with thionyl chloride in toluene. Relatively unstable compounds **5** and **6** were immediately used for the further step of preparation of the *N*-[4-(aminosulfonyl)phenyl]-4-alkyl-4*H*-furo[3,2-*b*]pyrrole-5-carboxamides **7a,c-f** and *N*-[4-(aminosulfonyl)phenyl]-4-alkyl-4*H*-thieno[3,2-*b*]pyrrole-5-carboxamides **8a-d,f**.

**Scheme 2:**
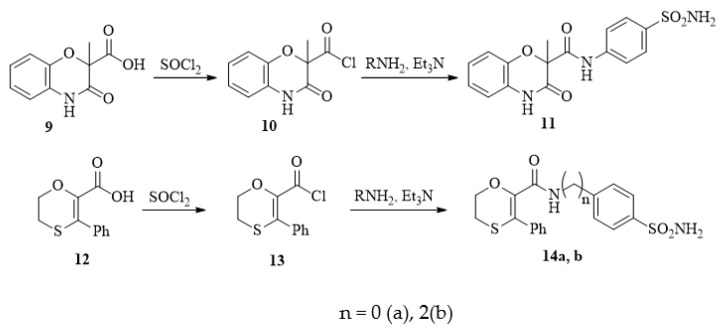
Synthesis of amides **11** and **14a**,**b**.

Synthesis of amides **11** and **14a,b** is shown in Scheme 2. 2-Methyl-3-oxo-3,4-dihydro-2*H*-1,4-benzoxazine-2-carboxylic acid **9 [42]** and 3-phenyl-5,6-dihydro-1,4-oxathiine-2-carboxylic acid **12 [43]** were converted into acid chlorides **10** and **13**, which without further purification upon reaction with corresponding amines led to **11** and **14a,b**.

**Scheme 3:**
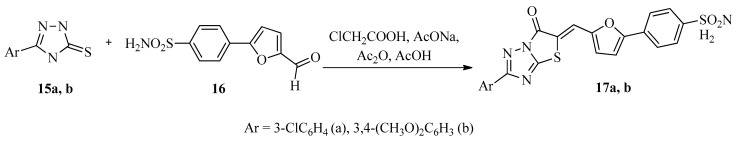
Synthesis of substituted 6-Oxo[1,3]thiazolo[3,2-*b*][1,2,4]triazole.

6-Oxo[1,3]thiazolo[3,2-*b*][1,2,4]triazole derivatives **17a,b** were synthesized according to pathway shown on Scheme 3. Compounds **17a,b** were obtained with high yields starting from 5-aryl-2,4-dihydro-3*H*-1,2,4-triazole-3-thiones **15a,b** [44] and 4-(5-formyl-2-furyl)-benzenesulfonamide **16** [45] according to the earlier proposed procedure [46,47,48].

The structure and composition of compounds **3a,c-f, 4a-d,f, 7a,c-f,** and **8a-d,f** were confirmed by ^1^H-NMR, ^13^C-NMR spectroscopy, and elemental analysis (Supplementary Materials) Thus, the signal of the carboxyl group of compounds **3**, **4**, and **7** were found as a singlet in the range of 10.0–10.4 ppm. The signals of the 4*H*-furo[3,2-*b*]pyrrole moiety of compounds **3** and **7** were observed at 7.70–7.80 ppm, while the signals of the 4*H*- thieno[3,2-*b*]pyrrole moiety of the compounds **4** and **8** at 7.50–7.60 ppm. The sulfonamide group was observed as a singlet at 7.20–7.30 ppm. On the other hand in the case of compounds **11** and **14a,b** NH protons signals were found as two singlets at 10.91 ppm and 10.49 ppm, while the signal of the sulfonamide group was as a singlet at 7.24 ppm. The peak of the methyl group appears as a singlet at 1.73 ppm, while of the methylidene group of **17a,b** CH= protons appear as a singlet at 8.20–8.10 ppm, deshielded by adjacent C=O group, at higher chemical shift values, indicating a predominant existence of Z-configuration of this group double bond in these compounds. The expected ones for E isomer have methine proton with a lesser deshielding effect [49,50].

### 2.2. Carbonic Anhydrase Inhibition

All compounds, here reported, were evaluated for their inhibitory activity against four human CA isoforms, namely: hCA I, hCA II, hCA IX, and hCA XII. The results are shown in Table 1.

According to obtained results it is obvious that all compounds displayed inhibition against all tested hCA isoforms, but with different range of inhibition constants. Thus, the K_i_ values of compounds against hCA I ranged from 56.5 to 918.5 nM, with compound **14a** exhibiting the best activity (K_i_ = 56.5 nM). Good activity was achieved also for compound **11** with K_i_ value of 70.6 nM, followed by compound **14b** (K_i_ = 71.2 nM) compared to **AAZ** (K_i_ = 250 nM). The lowest activity against hCA I was observed for compound **17b** with K_i_ values at 918.5 nM. The order of activity of compounds against hCA I can be presented as follows: **14a > 11 > 14b > 8b > 8c > 8d > 8a > 8f > 7a > 7e > 7f > 17b > 17a > 7c.**

The structure–activity relationship studies revealed that the presence of 3-phenyl-5,6-dihydro-1,4-dioxine-2-carboxamide (**14a**) as a substituent at the para- position of benzenesulfonamide is very beneficial for inhibitory activity against hCA I. It replacement by 2-methyl-3-oxo-3,4-dihydro-2*H*-benzo[b][1,4]oxazine-2-carboxamide led to compound **11** with decreased activity. The introduction to position 4 of the benzenesulfonamide 3-phenyl-*N*-propyl-5,6-dihydro-1,4-oxathiine-2-carboxamide substituent with prolonged chain between benzenesulfonamide and 5-phenyl-2,3-dihydro-1,4-oxathiine **14b** did not affect the activity much, only decreasing it slightly. Among furo- and thieno- derivatives it is obvious that in general thieno- derivatives are more potent inhibitors against hCA I than furo-, which are the least active (**7a, 7e, 7f**, and **7c**). Among *N*-(4-sulfamoylphenyl)-4*H*-thieno[3,2-*b*]pyrrole-5-carboxamide derivatives the presence of ethyl group on position 4 of *N*-(4-sulfamoylphenyl)-4*H*-thieno[3,2-*b*]pyrrole (**8b**) appeared to be beneficial. Replacement of ethyl by benzyl (**8c**) decreased a little activity, followed by 2-fluorobenzyl substituent (**8d**). The less active among thieno derivatives were found to be compound with 4-chlorobenzyl substituent, followed by four furo derivatives with the 4-benzyl-*N*-(4-sulfamoylphenyl)-4*H*-furo[3,2-*b*]pyrrole-5-carboxamide exhibiting the lowest inhibitory activity against hCA I isoform.

It is worth noticing that in general, these compounds showed better activity toward cytosolic hCA II isoform than against almost all other isoforms with K_i_ values in the range of 6.5–68.1 nM. Thus, the order of activity against hCA II isoform can be presented as **11** > **14a** > **8b** > **14b** > **7a** > **8d** > **8a** > **8c** > **7e** > **7f** > **7c** > **8f** > **17a** > **17b.** The highest activity was achieved for compound **11** with K_i_ = 6.5 nM, followed by **14a** (K_i_ = 6.7 nM) compared to **AAZ** with a K_i_ value of 12.1 nM. It is interesting to notice that the most active compounds against hCA I isoform were also active against hCA II isoform. It should be mentioned that compound **8a** was the most selective one with a selectivity index (SI) 75.5 towards hCA I, 7.6 compared to hCA IX, and 3.5 to hCA XII isoforms. Eight compounds exhibited higher activity against the cytosolic hCA II isoform than reference compound **AAZ**.

According to structure-activity relationship studies, the presence of 2-methyl-3-oxo-3,4-dihydro-2*H*-benzo[b][1,4]oxazine-2-carboxamide substituent on position 4 of benzenesulfonamide (**11**) was positive for inhibitory activity against hCA II isoform. The replacement it by 3-phenyl-5,6-dihydro-1,4-dioxine-2-carboxamide (**14a**) decreased a little the activity followed by 4-ethyl-4*H*-thieno[3,2-*b*]pyrrole-5-carboxamide (**8b**). The less potent inhibitor was, as in the case with hCA I isoform, thieno derivative 4-(4-chlorobenzyl)-*N*-(4-sulfamoylphenyl)-4*H*-thieno [3,2-*b*]pyrrole-5-carboxamide (**8f**).

As far as hCA IX isoform is concerned, the new compounds showed moderate to week inhibitory activity with K_i_ in the range of 81.4–320.0 nM compared to 25.7 nM of acetazolamide.

In the case of the hCA XII isoform, these compounds exhibited much better activity than against hCA IX and hCA I isoforms but were less effective compared to their activity on hCA II. Their K_i_ was in the range of 4.6–37.2 nM. The activity in descending order can be presented as follows: **7c** > **8b** > **11** > **14a** > **14b > 7a** > **17a** > **8c** > **8d** > **8f** > **7e** > **8a** > **7f** > **17b.** Three compounds (**7c, 8b, 11**) exhibited activity better than that of **AAZ** with compound **7c** displaying the highest activity (K_i_ = 4.6 nM) compared to **AAZ** (K_i_ = 5.7 nM). The lowest activity was shown by furo derivative **7f** (K_i_ = 37.2 nM). It is interesting to notice that compound **7c** (4-benzyl-*N*-(4-sulfamoylphenyl)-4*H*-furo[3,2-*b*]pyrrole-5-carboxamide) demonstrated the highest activity against the hCA XII isoform, being the less active against the hCA I and hCA II isoforms.

The structure–activity relationship studies revealed that the presence of 4-benzyl-4*H*-furo[3,2-*b*]pyrrole-5-carboxamide plays a positive role in inhibitory activity against the hCA XII isoform. Replacement of furan by thieno ring and benzyl by ethyl led to compound **8b** with slightly lower activity, while the introduction to position 4 of benzenesulfonamide 2-methyl-3-oxo-3,4-dihydro-2*H*-benzo[b][1,4]oxazine-2-carboxamide substituent (**11**) decreased more the activity. On the other hand, the presence of 4-(4-chlorobenzyl)-4*H*-furo[3,2-*b*]pyrrole-5-carboxamide as a substituent on benzenesulfonamide moiety appeared to be detrimental for h CA XII inhibitory activity. Finally, compounds **11**, **14a**, **14b** were found to be active against three hCA I, hCA II, and hCA XII isoforms.

### 2.3. Molecular Docking Studies

In an attempt to predict the probable inhibition mechanism of the tested compounds, molecular docking studies were performed. As a representative of the whole set of compounds, ligands **7c**, **8a**, **8b**, **11**, and **14a** were selected for docking studies. All human CAs isoforms have similar active site architecture. Their active site contains three conserved His94, His96, and His119 residues acting as zinc ligands and another two conserved residues that act as ‘‘gatekeepers’’ Thr199 and Glu105 [51,52,53]. However, these isoforms vary in the residues generally in the middle and to the exit of the active site cavity. The results of molecular docking studies of the tested compounds on hCA I, II, IX, and XII isoforms are presented in Table 2 and revealed that all tested compounds bind the enzymes, chelating the Zn (II) ion of the active site, in a deprotonated form, as anions (negative nitrogen of the sulfonamide group) [54].

**Figure 2:**
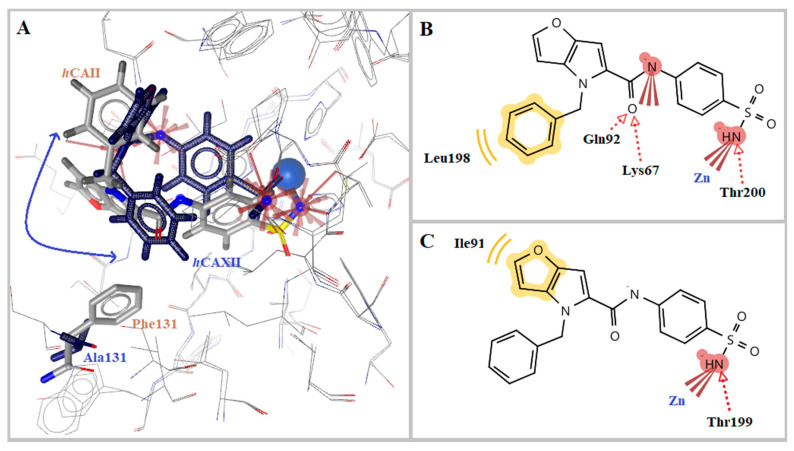
(**A**) Superposition of compound **7c** bound to hCA II (grey) in comparison to hCA XII (blue), with specific residues labeled. (**B**) 2D interaction diagram of compound **7c** docking pose interactions with the key amino acids in hCA XII and (**C**) in hCA II, Active site zinc is shown as a blue sphere, red dotted arrows indicate H-bond and yellow spheres hydrophobic interactions. Blue double-headed arrow indicates the direction of conformational change of the compound bound to hCA II in comparison to the hCA XII enzyme.

According to docking studies, the selectivity and the inhibition profile of some compounds to each isoform are depending on the differences in the active sites of the enzymes. More specifically, their inhibition profile is affected by the nature of the amino acids of the active site of the enzymes which determinate the conformation and interactions that compounds will adopt within the enzyme active site. For instance, compound **7c**, which has a K_i_ for the hCA II enzyme of 37.5 nM and a lower Ki for hCA XII of 4.6 nM, adopts different conformations when binding both hCAs. The main reason is probably the presence of the bulky hydrophobic residue Phe131 in the hCA II enzyme, unlike the smaller residue Ala131 in hCA XII. This smaller residue in the hCA XII enzyme, allows ligands to freely enter the active site of the enzyme and to adopt a conformation that favors interactions with residues in the hydrophobic pocket and increases the selectivity of the compound to this isoform (Figure 2). Moreover, the superposition of the two structures of hCAs bound to compound **7c** revealed this steric hindrance of the bulky residue Phe131 (Figure 2A). Consequently, **7c** adopts a different conformation within the active site of the hCA II enzyme with the benzene ring being removed away from the bulky Phe131, interacting less with the residues of the active site of the enzyme and forming a less stable complex ligand-enzyme. This is probably the reason that explains the higher experimental K_i_ of this compound for isoform hCA II. Furthermore, compound **7c** forms a hydrogen bond between the sulfonamide and the backbone of Thr199 of both isoforms and two more H-bonds between its carboxyl group and the backbones of residues Gln92 and Lys67 of isoform hCA XII, which further stabilize the complex ligand hCA XII and explains the high inhibition potency of the compound against this isoform (Figure 2B,C, Table 2).

**Figure 3:**
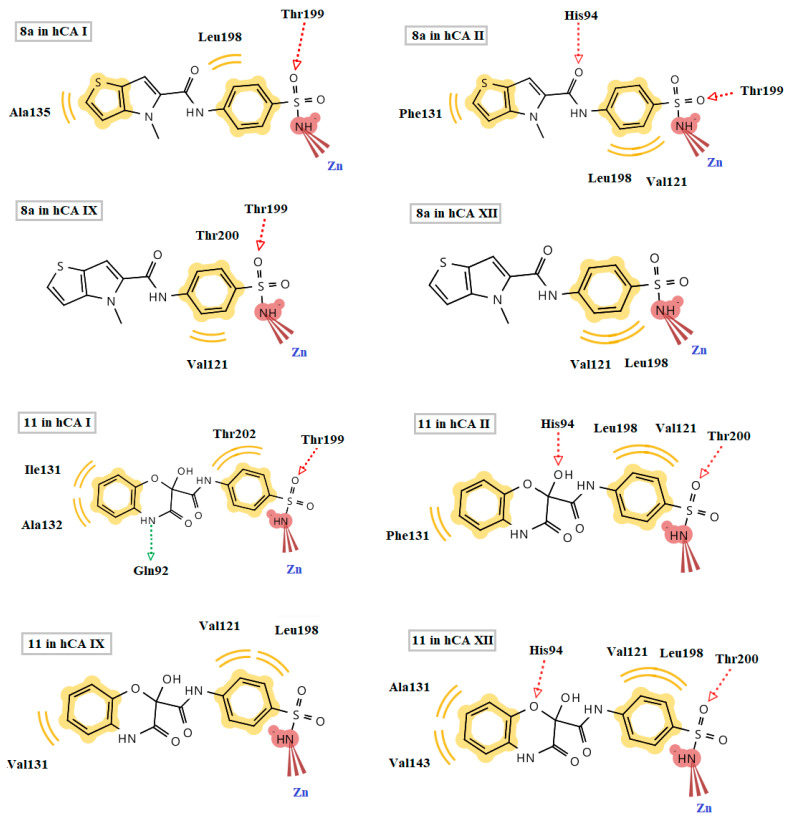
2D interaction diagrams of compounds **8a** and **11** docking poses interactions with the key amino acids in hCA I, hCA II, hCAIX, hCA XII.

**Figure 4:**
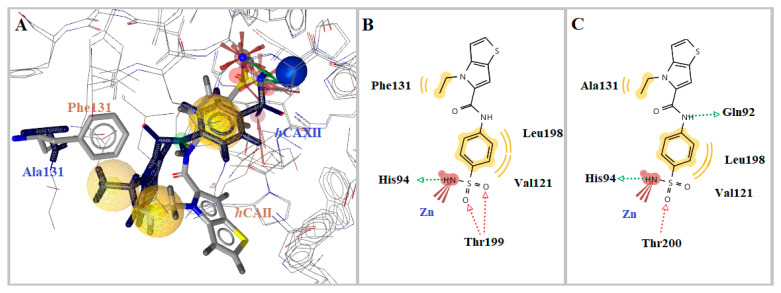
(**A**) Superposition of compound **8b** bound to hCA II (grey) in comparison to hCA XII (blue), with specific residues labeled, (**B**) 2D interaction diagram of compound **8b** docking pose interactions with the key amino acids in hCA II and (**C**) in hCA XII. Active site zinc is shown as a blue sphere, red dotted, and green arrows indicate H-bond and yellow spheres hydrophobic interactions.

On the other hand, compounds **8a** and **8b** (Figure 3) differ from compound **7c** by the presence of methyl and ethyl substituent instead of benzene (**7c**) on the indole ring. Both these substituents provide flexibility to the compounds, making them able to avoid the steric hindrance with the bulky residue Phe131 of hCA II isoform, increasing the inhibition potency. However, these compounds can also inhibit the hCA XII isoform. This is illustrated in Figure 4 where compound **8b** in both hCA II and hCA XII isoforms is adopting a conformation that favors the interactions with both active sites of the isoforms, increasing the stability of the complex and consequently the inhibition potency of the compound (Figure 4A). The negative nitrogen of the sulphonamide group coordinates to the Zn(II) ion and forms a hydrogen bond with conserved residue His94. In the hCA II isoform, both oxygen atoms of the sulphonamide group formed two hydrogen bonds interacting with the conserved residue Thr199, while in isoform hCA XII only one hydrogen bond with residue Thr200 is formed. Moreover, the ethyl substituent is interacting hydrophobically with Phe131 and Ala131 of hCA II and hCA XII isoforms respectively. Hydrophobic interactions between the benzene moiety and the residue Leu198 and Val121 in both isoforms (Figure 4B,C, Table 2) were also observed. These interactions stabilize further the complex ligand-enzyme and positively impacting into the inhibition profile of the compounds.

**Figure 5:**
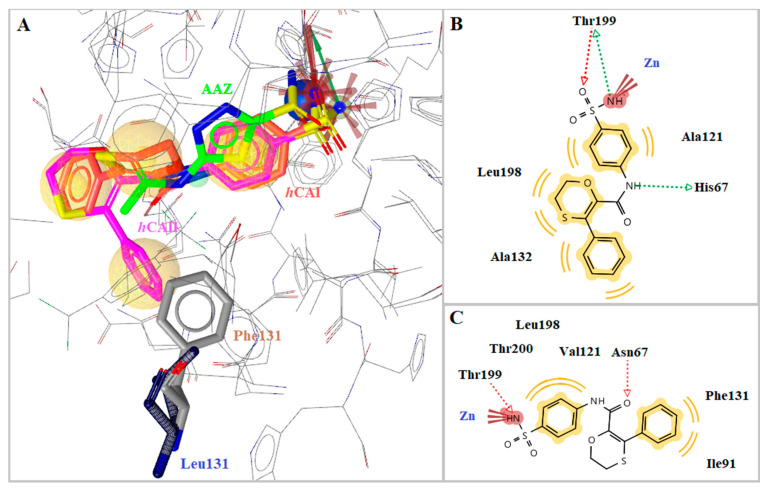
(**A**) Superposition of compound **14a** bound to hCA I (orange) in comparison to hCA II (magenta) and **AAZ** (green) in hCA I, (**B**) 2D interaction diagram of compound **14a** docking pose interactions with the key amino acids in hCA I, (**C**) in hCA II. Active site zinc is shown as a blue sphere, red dotted and green arrows indicate H-bond and yellow spheres hydrophobic interactions.

On the other hand, docking of compound **14a** into the active site of all CA isoforms revealed the probable reason for its good inhibition profile. As it is presented in Figure 5, compound **14a** binds hCA I and hCA II isoform in the same manner as **AAZ**, with the negative nitrogen of the sulphonamide group chelating the Zn(II) ion. Moreover, in the hCA II isoform the benzene moiety of the compound interacts hydrophobically with residues Phe131 and Ile91. These interactions increase the stability of the complex and probably explain its lower K_i_ than that of **AAZ** (6.7 nM vs. 12.1 nM).

### 2.4. In Silico Prediction Studies

**Figure 6:**
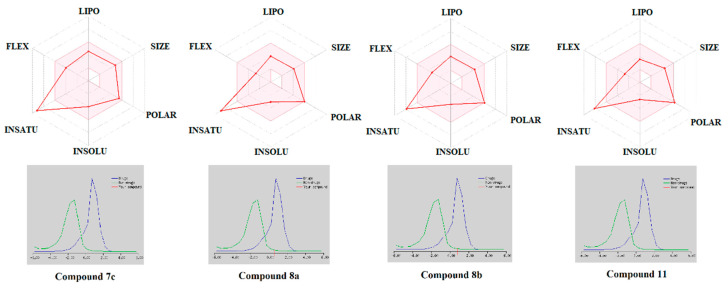
Bioavailability Radar of the tested compounds. The pink area represents the optimal range for each property for oral bioavailability, (Lipophilicity (LIPO): XLOGP3 between −0.7 and +5.0, Molecular weight (SIZE): MW between 150 and 500 g/mol, Polarity (POLAR) TPSA between 20 and 130 Å^2^, Solubility (INSOLU): log S not higher than 6, Saturation (INSATU): fraction of carbons in the sp3 hybridization not less than 0.25, and Flexibility (FLEX): no more than 9 rotatable bonds.

Drug likeness is examined as an important tool that provides the base for the molecules to be a powerful drug candidate. The number of violations to various rules viz. Lipinski, Ghose, Veber, Egan, and Muegge [55,56,57,58,59,60], along with bioavailability and Drug-likeness scores are given in Table 3. The results showed that none of the compounds violated any rule and their bioavailability score was around 0.55. All compounds exhibited moderate to good Drug-likeness scores ranged from −0.58 to 1.00. Moreover, the bioavailability radar of some of the compounds is displayed in Figure 6. The compound **8f** appeared to be the best in the in-silico predictions with a Drug-likeness score of 1.00 without any rule violation.

## 3. Materials and Methods

### 3.1. General

The solvents were purified according to the standard procedures. The ^1^H, ^13^C spectra were recorded on a Varian Unityplus-400 spectrometer (400 and 125 MHz, respectively) in a DMSO-*d*_6_ solution. Chemical shifts are reported in ppm downfield from TMS as internal standards. Mass spectra were recorded on an LC-MS instrument with chemical ionization (CI). LC-MS data were acquired on an Agilent 1200 HPLC system equipped with DAD/ELSD/LSMS-6120 diode matrix and mass-selective detector. Melting points were determined using a Fischer Johns instrument. Elemental analysis was performed at an analytical laboratory of the Institute of Bioorganic Chemistry and Petrochemistry, National Academy of Sciences of Ukraine.

### 3.2. General Procedure for the Synthesis of 4-Alkyl-4H-furo[3,2-b]pyrrole-5-carboxylic Acid 3a,c-f and 4-Alkyl-4H-thieno[3,2-b]pyrrole-5-carboxylic Acid **4a-d, f**

A solution of methyl 4*H*-furo[3,2-*b*]pyrrole-5-carboxylate **1** (20 mmol) [40] or methyl 4*H*-thieno[3,2-*b*]pyrrole-5-carboxylate **2** [41] in DMF (15 mL) was added dropwise at stirring under inert atmosphere to suspension of sodium hydride (1 g, 25 mmol, prepared from preliminarily washed with anhydrous hexanes 60% suspension of NaH in mineral oil) in DMF (15 mL). The mixture was stirred for 20 min, corresponding alkyl halide (25 mmol) was added, and the mixture was continuously stirred at 45 °C until completion (TLC control by disappearing of initial compound **1** or **2**). After cooling to ambient temperature acetic acid (1 mL) was added and the mixture was evaporated to dryness under reduced pressure. After addition water (50 mL), the formed precipitate was filtered off, dissolved in methanol (30 mL), and treated with a solution of potassium hydroxide (3.36 g, 60 mmol) in water (15 mL). The resulted mixture was stirred at 50 °C for 18 h (TLC control), cooled to ambient temperature, and acidified with formic acid. The formed precipitate was filtered off, washed with water, dried in air, and crystallized from ethanol.

4-Methyl-4*H*-furo[3,2-*b*]pyrrole-5-carboxylic acid (**3a**). Yield 82%; m.p. 158–160 °C. ^1^H-NMR (400 MHz, DMSO-*d*_6_, ppm) δ 12.29 (s, 1H, COOH), 7.78 (d, *J* = 2.3 Hz, 1H, H-2), 6.78 (d, *J* = 2.3 Hz, 1H, H-3), 6.75 (s, 1H, H-6), 3.91 (s, 3H, NCH_3_). ^13^C-NMR (125 MHz, DMSO-*d6*, ppm) δ 162.90, 148.94, 144.68, 133.21, 124.17, 98.94, 97.59, 34.60 Anal. Calcd. for C_8_H_7_NO_3_ (%): C, 58.18; H, 4.27; N, 8.48 Found (%): C, 58.11; H, 4.31; N, 8.39.

4-Benzyl-4*H*-furo[3,2-*b*]pyrrole-5-carboxylic acid (**3c**). Yield 87%; m.p. 152–154 °C (lit.129–130. °C [59]). ^1^H-NMR (400 MHz, DMSO-*d*_6_, ppm) δ 12.35 (s, 1H, COOH), 7.77 (d, *J* = 2.2 Hz, 1H, H-2), 7.35–7.11 (m, 5H, Ar), 6.86 (s, 1H, H-6), 6.68 (d, *J* = 2.2 Hz, 1H, H-3), 5.66 (s, 2H, NCH_2_). ^13^C-NMR (125 MHz, DMSO-*d*_6_, ppm) δ 162.75, 149.12, 145.00, 138.41, 132.73, 128.47, 127.30, 127.02, 123.63, 99.39, 98.51, 49.62. Anal. Calcd. for C_14_H_11_NO_3_ (%): C, 69.70; H 4.60; N 5.81 Found (%): C, 69.78; H, 4.54; N, 5.72.

4-(2-Fluorobenzyl)-4*H*-furo[3,2-*b*]pyrrole-5-carboxylic acid (**3d**). Yield 83%; m.p. 162–164 °C. ^1^H-NMR (400 MHz, DMSO-*d*_6_, ppm) δ 12.39 (s, 1H, COOH), 7.78 (d, *J* = 2.2 Hz, 1H, H-2), 7.35–7.29 (m, 1H, Ar), 7.25–7.17 (m, 1H, Ar), 7.14–7.07 (m, 1H, Ar), 6.88 (s, 1H, H-6), 6.86–6.79 (m, 1H, Ar), 6.56 (d, *J* = 2.2 Hz, 1H, H-3), 5.73 (s, 2H, NCH_2_). ^13^C-NMR (125 MHz, DMSO-*d*_6_, ppm) δ 162.63, 159.73 (d, J_CF_ = 244.9 Hz), 149.18, 144.98, 132.83, 129.46 (d, J_CF_ = 8.1 Hz), 128.60 (d, J_CF_ = 3.9 Hz), 125.33 (d, J_CF_ = 15.0 Hz), 124.62 (d, J_CF_ = 3.5 Hz), 123.85, 115,23 (d, J_CF_ = 20.9 Hz), 99.27, 98.75, 43.93 (d, J_CF_ = 4.6 Hz). Anal. Calcd. for C_14_H_10_FNO_3_ (%): C, 64.87; H, 3.89; N, 5.40 Found (%): C, 64.79; H, 3.97; N, 5.29.

4-(2-Chlorobenzyl)-4*H*-furo[3,2-*b*]pyrrole-5-carboxylic acid (**3e**). Yield 91%; m.p. 149–152 °C. ^1^H-NMR (400 MHz, DMSO-*d*_6_, ppm) δ 12.25 (s, 1H, COOH), 7.76 (d, *J* = 2.2 Hz, 1H, H-2),7.52–7.45 (m, 1H, Ar), 7.35–7.18 (m, 2H, Ar), 6.92 (s, 1H, H-6),6.57–6.49 (m, 1H, Ar), 6.47 (d, *J* = 2.2 Hz, 1H, H-3), 5.75 (s, 2H, NCH_2_). ^13^C-NMR (125 MHz, DMSO-*d*_6_, ppm) δ 162.52, 149.21, 145.06, 135.94, 132.80, 131.46, 129.24, 128.92, 127.52, 127.31, 124.08, 99.22, 98.77, 48.01. Anal. Calcd. for C_14_H_10_ClNO_3_ (%): C, 60.99; H, 3.66; Cl, 12.86; N, 5.08 Found (%): C, 61.10; H, 3.73; Cl, 12.65; N, 5,15.

4-(4-Chlorobenzyl)-4*H*-furo[3,2-*b*]pyrrole-5-carboxylic acid (**3f**). Yield 92%; m.p. 131–132 °C. ^1^H-NMR (400 MHz, DMSO-*d*_6_, ppm) δ 12.32 (s, 1H, COOH), 7.77 (d, *J* = 2.2 Hz, 1H, H-2),7.36 (d, *J* = 8.3 Hz, 2H, Ar), 7.16 (d, *J* = 8.3 Hz, 2H, Ar), 6.84 (s, 1H, H-6), 6.69 (d, *J* = 2.2 Hz, 1H, H-3), 5.65 (s, 2H, NCH_2_)). ^13^C-NMR (125 MHz, DMSO-*d*_6_, ppm) δ 162.69, 149.24, 145.00, 137.47, 132.71, 131.92, 128.79, 128.47, 123.54, 99.30, 98.67, 48.94. Anal. Calcd. for C_14_H_10_ClNO_3_ (%): C, 60.99; H, 3.66; Cl, 12.86; N, 5.08 Found (%): C, 61.13; H, 3.75; Cl, 12.71; N, 5,04.

4-Methyl-4*H*-thieno[3,2-*b*]pyrrole-5-carboxylic acid (**4a**). Yield 88%; m.p. 168–169 °C (lit.172–174 °C [60]). ^1^H-NMR (400 MHz, DMSO-*d*_6_, ppm) δ 12.45 (s, 1H, COOH), 7.54 (d, *J* = 5.4 Hz, 1H, H-2), 7.21 (d, *J* = 5.4 Hz, 1H, H-3), 7.11 (s, 1H, H-6), 3.99 (s, 3H, NCH_3_). ^13^C-NMR (125 MHz, DMSO-*d*_6_, ppm) δ 162.50, 145.45, 129.29, 127.03, 120.73, 111.20, 108.70, 34.40. Anal. Calcd. for C_8_H_7_NO_2_S (%): C, 53.03; H, 3.89; N, 7.73; S, 17.69 Found (%): C, 53.15; H, 3.92; N, 7.61; S, 17.91.

4-Ethyl-4*H*-thieno[3,2-*b*]pyrrole-5-carboxylic acid (**4b**). Yield 83%; m.p. 155–156 °C. ^1^H-NMR (400 MHz, DMSO-*d*_6_, ppm) δ 12.47 (s, 1H, COOH), 7.55 (d, *J* = 5.4 Hz, 1H, H-2), 7.24 (d, *J* = 5.4 Hz, 1H, H-3), 7.13 (s, 1H, H-6), 4.51 (q, *J* = 7.1 Hz, 2H, NCH_2_), 1.29 (t, *J* = 7.1 Hz, 3H, CH_3_). ^13^C-NMR (125 MHz, DMSO-*d*_6_, ppm) δ 162.25, 144.46, 129.39, 126.05, 120.97, 111.09, 109.03, 41.69, 16.32.Anal. Calcd. for C_9_H_9_NO_2_S (%): C, 55.37; H, 4.65; N, 7.17; S, 16.42 Found (%): C, 55.51; H. 4.72; N, 7.03; S, 16.64.

4-Benzyl-4*H*-thieno[3,2-*b*]pyrrole-5-carboxylic acid (**4c**). Yield 89%; m.p. 180–181 °C (lit.180–182 °C [61]). ^1^H-NMR (400 MHz, DMSO-*d*_6_, ppm) δ 12.36 (s, 1H, COOH), 7.53 (d, *J* = 5.4 Hz, 1H, H-2), 7.31–7.19 (m, 4H, H-6, Ar), 7.17 (d, *J* = 5.4 Hz, 1H, H-3), 7.12 (d, *J* = 7.4 Hz, 2H, Ar), 5.78 (s, 2H, NCH_2_). ^13^C-NMR (125 MHz, DMSO-*d*_6_, ppm) δ 162.45, 145.14, 138.59, 129.79, 128.49, 127.22, 126.69, 126.48, 121.45, 111.54, 109.63, 49.40. Anal. Calcd. for C_14_H_11_NO_2_S (%): C, 65.35; H, 4.31; N, 5.44; S, 12.46 Found (%): 65.50; H, 4.42; N, 5,59; S, 12.70.

4-(2-Fluorobenzyl)-4*H*-thieno[3,2-*b*]pyrrole-5-carboxylic acid (**4d**). Yield 86%; m.p. 179–181 °C. ^1^H-NMR (400 MHz, DMSO-*d*_6_, ppm) δ 12.49 (s, 1H, COOH), 7.55 (d, *J* = 5.4 Hz, 1H, H-2), 7.33–7.24 (m, 2H, H-6, Ar), 7.23–7.16 (m, 1H, Ar), 7.13 (d, *J* = 5.4 Hz, 1H, H-3), 7.09–7.04 (m, 1H, Ar), 6.69–6.63 (m, 1H, Ar), 5.84 (s, 2H, NCH_2_)). ^13^C-NMR (125 MHz, DMSO-*d*_6_, ppm) δ 162.33, 159.53 (d, J_CF_ = 244.8 Hz), 145.25, 129.87, 129.18 (d, J_CF_ = 8.1 Hz), 127.87 (d, J_CF_ = 4.1 Hz), 126.76, 125.65 (d, J_CF_ = 14.6 Hz), 124.62 (d, J_CF_ = 3.4 Hz), 121.54, 115.24 (d, J_CF_ = 21.0 Hz), 111.33, 109.80, 43.76 (d, J_CF_ = 4.8 Hz). Anal. Calcd. for C_14_H_10_FNO_2_S (%): C, 61.08; H, 3.66; N, 5.09; S, 11.65 Found (%): C, 60.87; H, 3.73; N, 5.17; S, 11.88.

4-(4-Chlorobenzyl)-4*H*-thieno[3,2-*b*]pyrrole-5-carboxylic acid (**4f**). Yield 91%; m.p. 173–174 °C. ^1^H-NMR (400 MHz, DMSO-*d*_6_, ppm) δ 10.50 (s, 1H, COOH), 7.58 (d, *J* = 5.4 Hz, 1H, H-2),7.35 (d, *J* = 8.3 Hz, 2H, Ar), 7.23 (d, *J* = 5.4 Hz, 1H, H-3), 7.22 (s, 1H, H-6), 7.11 (d, *J* = 8.3 Hz, 2H, Ar), 5.76 (s, 2H, NCH_2_). ^13^C-NMR (125 MHz, DMSO-*d*_6_, ppm) δ 162.38, 145.02, 137.59, 131.81, 129.89, 128.52, 128.46, 126.44, 121.51, 111.41, 109.71, 48.77. Anal. Calcd. for C_14_H_10_ClNO_2_S (%): C, 57.64; H, 3.45; Cl, 12.15; S, 10.99 Found (%): C, 57.49; H, 3.53; Cl, 11.97; S, 12.09.

### 3.3. General Procedure for the Synthesis of 4-Alkyl-4H-furo[3,2-*b*]pyrrole-5-carbonyl Chloride 5a,c-f and 4-Alkyl-4H-thieno[3,2-b]pyrrole-5-carbonyl Chloride **6a-d,f**

To a stirred solution of compounds **3a,c-f** or **4a-d,f** (5 mmol) in anhydrous toluene (30 mL) thionyl chloride (3 mL) was added. The mixture was heated for 1 h at 80 ^°^C, evaporated to dryness under reduced pressure, the residue was treated with 10 mL of toluene and the resulted mixture was continuously evaporated to dryness under reduced pressure. The residue was dissolved in the boiling mixture (100 mL of cyclohexane and 75 mL of n-heptane), charcoal (0.5 g) was added, and after stirring for 3 min, filtered, evaporated to dryness under reduced pressure to afford **5a,c-f** and **6a-d,f** as light-yellow crystals. These obtained acid chlorides were immediately used for amides **7** and **8** preparation. The compounds **5a,c-f** and **6a-d,f** are quite unstable (**5a,c-f** decomposed after 2 h of standing, **6a-d,f** decomposed after 8h of standing at ambient temperature).

### 3.4. General Procedure for the Synthesis of N-[4-(aminosulfonyl)phenyl]-4-alkyl-4H-furo[3,2-b]pyrrole-5-carboxamide 7a,c-f and N-[4-(aminosulfonyl)phenyl]-4-alkyl-4H-thieno[3,2-b]pyrrole-5-carboxamide **8a-d, f**

To an ice-water cooled stirred solution of 4-aminobenzenesulfonamide (0.17 g (1 mmol) and of triethylamine (0.11 g, 1.1 mmol) in acetonitrile (5 mL) the solution of compounds **5a,c-f** or **6a-d,f** (1 mmol) in acetonitrile (10 mL) was added. The mixture was stirred for 1 h at ambient temperature and evaporated to dryness under reduced pressure. After the addition of water (15 mL), the formed precipitate was filtered off, dried in air, and crystallized from the DMF:ethanol mixture.

*N*-[4-(aminosulfonyl)phenyl]-4-methyl-4*H*-furo[3,2-*b*]pyrrole-5-carboxamide (**7a**). Yield 92%; m.p. 273–275 °C. ^1^H-NMR (400 MHz, DMSO-*d*_6_, ppm) δ 10.06 (s, 1H, NH), 7.96–7.73 (m, 5H, Ar, H-2), 7.25 (c, 2H, NH_2_), 7.12 (c, 1H, H-6, 6.82 (d, *J* = 2.2 Hz,, 1H, H-3), 3.95 (c, 3H, NCH_3_). ^13^C-NMR (125 MHz, DMSO-*d*_6_, ppm) 161.03, 149.19, 145.05, 142.85, 138.50, 133.26, 126.98, 126.93, 119.84, 99.48, 96.66, 35.37. MS (APCI): *m/z* = 320.0 [M + H]^+^; *m/z* = 317.8 [M−H]^−^.Anal. Calcd. for C_14_H_13_N_3_O_4_S (%): C, 52.66; H, 4.10; N, 13.16; S, 10.04 Found (%): C, 52.48; H, 4.18; N13.04; S, 10.26.

*N*-[4-(aminosulfonyl)phenyl]-4-benzyl-4*H*-furo[3,2-*b*]pyrrole-5-carboxamide (**7c**). Yield 87%; m.p. 240–241 °C. ^1^H-NMR (400 MHz, DMSO-*d*_6_, ppm) δ 10.12 (s, 1H, NH), 7.89 (d, *J* = 8.6 Hz, 2H, Ar), 7.83–7.73 (m, 3H, H-2, Ar), 7.34–7.16 (m, 8H, H-6, Ar, NH_2_), 6.69 (d, *J* = 2.2 Hz, 1H, H-3), 5.72 (s, 2H, NCH_2_). ^13^C-NMR (125 MHz, DMSO-*d*_6_, ppm) δ 160.98, 149.30, 145.47, 142.70, 138.96, 138.59, 132.79, 128.92, 127.76, 127.55, 126.96, 126.57, 119.90, 99.91, 96.68, 50.30. MS (APCI): *m/z* = 396.0 [M + H]^+^; *m/z* = 394.0 [M−H]^−^. Anal. Calcd. for C_20_H_17_N_3_O_4_S (%): C, 60.75; H, 4.33; N, 10.63; S, 8.11 Found (%): C, 60.67; H, 4.29; N, 10.49; S, 8.34.

*N*-[4-(aminosulfonyl)phenyl]-4-(2-fluorobenzyl)-4*H*-furo[3,2-*b*]pyrrole-5-carboxamide **(7d**). Yield 89%; m.p. 240–242 °C. ^1^H-NMR (400 MHz, DMSO-*d*_6_, ppm) δ 10.15 (s, 1H, NH), 7.86 (d, *J* = 8.7 Hz, 2H, Ar), 7.80–7.74 (m, 3H, H-2, Ar), 7.36–7.17 (m, 5H, H-6, NH_2_, Ar), 7.13–7.07 (m, 1H, Ar), 6.95–6.89 (m, 1H, Ar), 6.60 (d, *J* = 2.3 Hz, 1H, H-3), 5.79 (s, 2H, NCH_2_). ^13^C-NMR (125 MHz, DMSO-*d*_6_, ppm) δ 160.4, 159.76 (d, *J*_CF_ = 245 Hz), 148.87, 144.99, 142.19, 138.19, 132.39, 129.46 (d, *J*_CF_ = 8.1 Hz), 128.81 (d, *J*_CF_ = 4.0 Hz), 126.50, 126.34, 125.39 (d, *J*_CF_ = 14.8 Hz), 124.59 (d, *J*_CF_ = 3.4 Hz), 119.45, 115.25 (d, *J*_CF_ = 20.9 Hz), 99.34, 96.42, 44.14 (d, *J*_CF_ = 4.4 Hz). MS (APCI): *m/z* = 414.0 [M + H]^+^; *m/z* = 412.0 [M-H]. Anal. Calcd. for C_20_H_16_FN_3_O_4_S (%): C, 58.10; H, 3.90; N, 10.16; S, 7.76 Found (%): C, 58.18; H, 3.96; N, 9.97; S, 7.89.

*N*-[4-(aminosulfonyl)phenyl]-4-(2-chlorobenzyl)-4*H*-furo[3,2-*b*]pyrrole-5-carboxamide (**7e**). Yield 93%; m.p. 239–241 °C. ^1^H-NMR (400 MHz, DMSO-*d*_6_, ppm) δ 10.14 (s, 1H, NH), 7.83 (d, *J* = 8.6 Hz, 2H, Ar), 7.78–7.71 (m, 3H, H-2, Ar), 7.51–7.45 (m, 1H, Ar), 7.38–7.19 (m, 5H, H-6, Ar, NH_2_), 6.62–6.57 (m, 1H, Ar), 6.53 (d, *J* = 2.2 Hz,, 1H, H-3), 5.80 (s, 2H, NCH_2_). ^13^C-NMR (125 MHz, DMSO-*d*_6_, ppm) δ 160.75, 149.42, 145.51, 142.58, 138.62, 136.48, 132.88, 131.87, 129.71, 129.37, 127.93, 127.89, 126.93, 126.86, 119.86, 99.77, 96.85, 48.73. MS (APCI): *m/z* = 432.0([M(^37^Cl)+H]^+^; 30); *m/z* = 430.0([M(^35^Cl)+H]^+^, 100); *m/z* = 430.0 ([M(^37^Cl)-H]^−^, 30); *m/z* = 428.0 ([M(^37^Cl)-H]^−^, 100). Anal. Calcd. for C_20_H_16_ClN_3_O_4_S (%): C, 55.88; H, 3.75; N, 9.77; S, 7.46 Found (%): C, 55.75; H, 3.77; N, 9.92; S, 7.62.

*N*-[4-(aminosulfonyl)phenyl]-4-(4-chlorobenzyl)-4*H*-furo[3,2-*b*]pyrrole-5-carboxamide (**7f**). Yield 94%; m.p. 237–238 °C. ^1^H-NMR (400 MHz, DMSO-*d*_6_, ppm) δ 10.13 (s, 1H, NH), 7.87 (d, *J* = 8.7 Hz, 2H, Ar), 7.81–7.74 (m, 3H, H-2, Ar), 7.37 (d, *J* = 8.5 Hz, 2H, Ar), 7.26 (s, 2H, NH_2_), 7.24–7.19 (m, 3H, H-6, Ar), 6.76 (d, *J* = 2.2 Hz, 1H, H-3), 5.70 (s, 2H, NCH_2_). ^13^C-NMR (125 MHz, DMSO-*d*_6_, ppm) δ 160.45, 148.98, 145.04, 142.18, 138.19, 137.57, 132.33, 131.92, 128.93, 128.47, 126.50, 126.00, 119.49, 99.38, 99.36, 49.21. MS (APCI): *m/z* = 432.0 ([M(^37^Cl)+H]^+^; 30); *m/z* = 430.0 ([M(^35^Cl)+H]^+^, 100); *m/z* = 429.8 ([M(^37^Cl)-H]^−^, 30); *m/z* = 427.8 ([M(^37^Cl)-H]^−^, 100). Anal. Calcd. for C_20_H_16_ClN_3_O_4_ (%): C, 55.88; H, 3.75; N, 9.77; S, 7.46 Found (%): C, 55.83; H, 3.71; N, 9.89; S, 7.59.

*N*-[4-(aminosulfonyl)phenyl]-4-methyl-4*H*-thieno[3,2-*b*]pyrrole-5-carboxamide (**8a**).Yield 95%; m.p. 276–278 °C. ^1^H-NMR (400 MHz, DMSO-*d*_6_, ppm) δ 10.29 (s, 1H, NH), 7.93 (d, *J* = 8.8 Hz, 2H, Ar), 7.79 (d, *J* = 8.8 Hz, 2H, Ar), 7,55 (d, *J* = 5.4 Hz, 1H, H-2), 7.40 (s, 1H, H-6), 7.31–7.23 (m, 3H, H-3, NH_2_), 4.03 (s, 3H, NCH_3_). ^13^C-NMR (125 MHz, DMSO-*d*_6_, ppm) δ 160.25, 145.13, 142.30, 138.25, 129.65, 128.77, 126.55, 120.53, 119.47, 111.22, 105.87, 34.55. MS (APCI): *m/z* = 336.1 [M + H]^+^; *m/z* = 334.0 [M−H]^−^. Anal. Calcd. for C_14_H_13_N_3_O_3_S_2_ (%): C, 50.14; H, 3.91; N, 12.53; S, 19.12 Found (%): C, 50.03; H, 3.97; N, 12.32; S, 19.27.

*N*-[4-(aminosulfonyl)phenyl]-4-ethyl-4*H*-thieno[3,2-*b*]pyrrole-5-carboxamide (**8b**). Yield 88%; m.p. 248–249 °C. ^1^H-NMR (400 MHz, DMSO-*d*_6_, ppm) δ 10.28 (s, 1H, NH), 7.92 (d, *J* = 8.8 Hz, 2H, Ar), 7.79 (d, *J* = 8.8 Hz, 2H, Ar), 7,55 (d, *J* = 5.4 Hz, 1H, H-2), 7.40 (s, 1H, H-6), 7.30–7.22 (m, 3H, H-3, NH_2_), 4.54 (q, *J* = 7.1 Hz, 2H, NCH_2_), 1.34 (t, *J* = 7.1 Hz, 3H CH_3_). Anal. Calcd. for C_15_H_15_N_3_O_3_S_2_ (%): C, 51.56; H, 4.33; N, 12.03; S, 18.35 Found (%): C, 51.64; H, 4.39; N, 11.89; S, 18.57.

*N*-[4-(aminosulfonyl)phenyl]-4-benzyl-4*H*-thieno[3,2-*b*]pyrrole-5-carboxamide (**8c**). Yield 87%; m.p. 255–256 °C. ^1^H-NMR (400 MHz, DMSO-*d*_6_, ppm) δ 10.29 (s, 1H, NH), 7.89 (d, *J* = 8.5 Hz, 2H, Ar), 7.79 (d, *J* = 8.5 Hz, 2H, Ar), 7,53 (d, *J* = 5.3 Hz, 1H, H-2), 7.46 (s, 1H, H-6), 7.31–7.13 (m, 8H, H-3, Ar, NH_2_), 5.82 (s, 2H, NCH_2_). ^13^C-NMR (125 MHz, DMSO-*d*_6_, ppm) δ 160.25, 144.77, 142.15, 138.62, 138.33, 129.21, 129.18, 128.46, 127.22, 126.80, 126.53, 121.21, 119.49, 111.56, 106.82, 49.54. MS (APCI): *m/z* = 412.0 [M + H]^+^; *m/z* = 409.8 [M−H]^−^. Anal. Calcd. for C_20_H_17_N_3_O_3_S_2_ (%): C, 58.38; H, 4.16; N, 10.21; S, 15.58 Found (%): C, 58.25; H, 4.22; N, 10.32; S, 15.76.

*N*-[4-(aminosulfonyl)phenyl]-4-(2-fluorobenzyl)-4*H*-thieno[3,2-*b*]pyrrole-5-carboxamide (**8d**). Yield 91%; m.p. 235–237 °C. ^1^H-NMR (400 MHz, DMSO-*d*_6_, ppm) δ 10.36 (s, 1H, NH), 7.86 (d, *J* = 8.7 Hz, 2H, Ar), 7.76 (d, *J* = 8.7 Hz, 2H, Ar), 7,55 (d, *J* = 5.4 Hz, 1H, H-2), 7.48 (s, 1H, H-6), 7.30–7.28 (m, 5H, H-3, Ar, NH_2_), 7.08–7.02 (m, 1H, Ar), 6.79–6.72 (m, 1H, Ar), 5.88 (s, 2H, NCH_2_). MS (APCI): *m/z* = 430.0 [M + H]^+^; *m/z* = 428.0 [M−H]^−^. Anal. Calcd. for C_20_H_16_FN_3_O_3_S_2_ (%): C, 55.93; H, 3.75; N, 9.78; S, 14.93 Found (%): C, 55.79; H, 3.81; N, 9.96; S, 15.14.

*N*-[4-(aminosulfonyl)phenyl]-4-(4-chlorobenzyl)-4*H*-thieno[3,2-*b*]pyrrole-5-carboxamide (**8f**). Yield 90%; m.p. 219–220 °C. ^1^H-NMR (400 MHz, DMSO-*d*_6_, ppm) δ 10.33 (s, 1H, NH), 7.88 (d, *J* = 8.6 Hz, 2H, Ar), 7.78 (d, *J* = 8.6 Hz, 2H, Ar), 7,56 (d, *J* = 5.3 Hz, 1H, H-2), 7.48 (s, 1H, H-6), 7.35 (d, *J* = 8.4 Hz, 2H, Ar), 7.26–7.20 (m, 3H, H-3, NH_2_), 7.18 (d, *J* = 8.4 Hz, 2H, Ar), 5.80 (s, 2H, NCH_2_). MS (APCI): *m/z* = 447.8 ([M(^37^Cl)+H]^+^; 30); *m/z* = 446.0 ([M(^35^Cl)+H]^+^, 100); *m/z* = 448.8 ([M(^37^Cl)-H]^−^, 30); *m/z* = 443.8 ([M(^37^Cl)-H]^−^, 100). Anal. Calcd. for C_20_H_16_ClN_3_O_3_S_2_ (%): C, 53.87; H, 3.62; N, 9.42; S, 14.38 Found (%): C, 54.01; H, 3.59; N, 9.57; S, 14.60.

### 3.5. Synthesis of N-[4-(aminosulfonyl)phenyl]-2-methyl-3-oxo-3,4-dihydro-2H-1,4-benzoxazine-2-carboxamide (**11**)

To a stirred solution of **9** (0.21 g, 1 mmol) [42] in of anhydrous toluene (10 mL) thionyl chloride (1 mL) was added. The mixture was heated for 1 h at 80 °C and then evaporated to dryness under reduced pressure, the residue was treated with toluene (5mL) and the resulted mixture was continuously evaporated to dryness under reduced pressure. The residue was dissolved in acetonitrile (10 mL) and then was added to an ice-water cooled solution of 4-aminobenzenesulfonamide (0.17 g, 1 mmol) and of triethylamine (0.11 g, 1.1 mmol) in acetonitrile. (5 mL). The mixture was stirred for 1 h, evaporated to dryness under reduced pressure. The residue was treated with water (15 mL), the formed precipitate was filtered off, dried in air, and crystallized from the DMF-ethanol mixture (1:3 *v/v*) to afford **11**. Yield 91%; m.p. 269–270 °C. ^1^H-NMR (400 MHz, DMSO-*d*_6_, ppm) δ 10.91 (s, 1H, NH), 10.49 (s, 1H, NH), 7.78–7.68 (m, 4H, Ar), 7.24 (s, 2H, NH_2_), 7.18–7.12 (m, 1H, Ar), 7.02–6.88 (m, 3H, Ar), 1.73 (s, 3H, CH_3_). MS (APCI): *m/z* = 362.0 [M + H]^+^; *m/z* = 360.0 [M−H]^−^. Anal. Calcd. for C_16_H_15_N_3_O_5_S (%): C, 53.18; H, 4.18; N, 11.63; S, 8.87 Found (%): C, 53.29; H, 4.15; N, 11.81; S, 9.03.

### 3.6. Synthesis of N-[4-(aminosulfonyl)phenyl]-3-phenyl-5,6-dihydro-1,4-oxathiine-2-carboxamide (**14a**)

To a stirred solution of compound **12** (0.22 g, 1 mmol) [43] in anhydrous benzene (10 mL) thionyl chloride (1 mL) was added. The mixture was stirred at 48–50 C for 3 h, evaporated to dryness under reduced pressure, the residue was dissolved in acetonitrile (10 mL) and this solution was added to an ice-water cooled solution of 4-aminobenzenesulfonamide (0.17 g, 1 mmol) and triethylamine (0.11 g, 1.1 mmol) in acetonitrile (5 mL). The mixture was stirred for 1 h at ambient temperature and evaporated to dryness under reduced pressure, the residue was treated with water (15 mL), the formed precipitate was filtered off and crystallized from ethanol to afford **14a**. Yield 93%; m.p. 233–235 °C. ^1^H-NMR (400 MHz, DMSO-*d*_6_, ppm) δ 10.08 (s, 1H, NH), 7.75–7.67 (m, 4H, Ar), 7.35–7.26 (m, 5H, Ar), 7.23 (s, 2H, NH_2_), 4.40–4.35 (m. 2H, OCH_2_), 3.32–3.24 (m, 2H, SCH_2_). ^13^C-NMR (125 MHz, DMSO-*d*_6_, ppm) δ 160.67, 141.98, 139.46, 138.96, 137.69, 129.14, 128.47, 128.25, 126.89, 119.75, 119.07, 64.93, 27.87. MS (APCI): *m/z* = 377.1 [M + H]^+^; *m/z* = 375.0 [M−H]^−^. Anal. Calcd. for C_17_H_16_N_2_O_4_S_2_ (%): C, 54.24; H, 4.28; N, 7.44; S, 17.03 Found (%): C, 54.31; H, 4.34; N, 7.56; S, 17.29.

*N*-{2-[4-(aminosulfonyl)phenyl]ethyl}-3-phenyl-5,6-dihydro-1,4-oxathiine-2-carboxamide (**14b**), was obtained similarly to **14a**. Yield 87%; m.p. 208–209 °C. ^1^H-NMR (400 MHz, DMSO-*d*_6_, ppm) δ 7.98 (t, *J* = 5.9 Hz, 1H, NH), 7.73 (d, *J* = 8.3 Hz, 2H, Ar), 7.33–7.15 (m, 9H, Ar, NH_2_), 4.32–4.24 (m, 2H, OCH_2_), 3.27–3.15 (m, 4H, NHCH_2_, OCH_2_), 2.73–2.65 (m, 2H, CH_2_Ar). ^13^C-NMR (125 MHz, DMSO-*d*_6_, ppm) δ 161.85, 144.00, 142.44, 139.94, 138.07, 129.42, 129.23, 128.22, 127.88, 126.1, 116.24, 64.79, 35.00, 27.68. MS (APCI): *m/z* = 405.1 [M + H]^+^; *m/z* = 403.0 [M−H]^−^. Anal. Calcd. for C_19_H_20_N_2_O_4_S_2_ (%): C, 56.42; H, 4.98; N, 6.93: S, 15.85 Found (%): C, 56.30; H, 5.07; N, 6.85; S, 16.03.

### 3.7. Synthesis of 4-(5-{(Z)-[2-(3-chlorophenyl)-6-oxo[1,3]thiazolo[3,2-b][1,2,4]triazol-5(6H)-ylidene]methyl}-2-furyl)enzenesulfonamide (**17a**)

A solution of compound **15a** (0.42 g, 2 mmol) [44], chloroacetic acid (0.2 g, 2 mmol), 4-(5-formyl-2-furyl) benzenesulfonamide (0.5 g, 2 mmol) **16** [45], and of sodium acetate (0.49 g, 6 mmol) in the mixture of acetic acid (10 mL) and of acetic anhydride (4 mL) was refluxed for 4 h. The reaction mixture was kept overnight, the formed precipitate was filtered off, washed with cool water, dried and crystallized from the DMF:ethanol mixture (1:1 *v/v*) to afford **17a**.Yield 78%; m.p. 312–315 °C. ^1^H-NMR (400 MHz, DMSO-*d*_6_, ppm) δ 8.14 (s, 1H, CH=), 8.11–7.92 (m, 6H, Ar), 7.58–7.51 (m, 2H, Ar), 7.44–7.14 (m, 2H, Ar), 7.35 (s, 2H, NH_2_). Anal. Calcd. for C_21_H_13_ClN_4_O_4_S_2_ (%): C, 52.01; H, 2.70; N, 11.55; S, 13.22 Found (%): C, 51.83; H, 2.74; N, 11.72; S, 13.39.

4-(5-{(Z)-[2-(3,4-Dimethoxyphenyl)-6-oxo[1,3]thiazolo[3,2 *b*][1,2,4]triazol-5(6H)-ylidene]methyl} -2-furyl)benzenesulfonamide (**17b**) was obtained similarly to **17a** starting from **15b** [44]. Yield 71%; m.p. 294–295 °C. ^1^H-NMR (400 MHz, DMSO-*d*_6_, ppm) δ 8.10 (s, 1H, CH=), 8.02 (d, *J* = 8.5 Hz, 2H, Ar), 7.95 (d, *J* = 8.5 Hz, 2H, Ar), 7.67 (d, *J* = 8.4 Hz, 1H, Ar), 7.57 (s, 1H, Ar), 7.44–7.37 (m, 4H, NH_2_, Ar), 7.03 (d, *J* = 8.4 Hz, 1H, Ar), 3.86 (s, 3H, OCH_3_), 3.84 (s, 3H, OCH_3_). ^13^C-NMR (125 MHz, DMSO-*d*_6_, ppm) δ 168.36, 160.39, 156.50, 155.71, 151.18, 149.38, 148.78, 144.09, 131.06, 126.68, 124.89, 123.67, 121.34, 120.19, 112.02, 111.65, 109.27, 55.52, 55.37. MS (APCI): *m/z* = 511.0 [M + H]^+^; *m/z* = 509.0 [M−H]^−^. Anal. Calcd. for C_23_H_18_N_4_O_6_S_2_ (%): C, 54.11; H, 3,55; N, 10.97; S, 12.56 Found (%): C, 53.96; H, 3.62; N, 10.79; S, 12.79.

### 3.8. Carbonic Anhydrase Inhibition

An Applied Photophysics stopped-flow instrument was used for assaying the CA catalyzed CO_2_ hydration activity [61]. Phenol red (at a concentration of 0.2 mM) was used as an indicator, working at the absorbance maximum of 557 nm, with 20 mM Hepes (pH 7.4) as a buffer, and 10 mM Na_2_SO_4_ (for maintaining constant ionic strength), following the initial rates of the CA-catalyzed CO_2_ hydration reaction for a period of 10–100 s. The CO_2_ concentrations ranged from 1.7 to 17 mM for the determination of the kinetic parameters and inhibition constants [10]. Enzyme concentrations ranged between 5–12 nM. For each inhibitor, at least six traces of the initial 5–10% of the reaction were used for determining the initial velocity. The uncatalyzed rates were determined in the same manner and subtracted from the total observed rates. Stock solutions of the inhibitor (0.1 mM) were prepared in distilled–deionized water and dilutions up to 0.01 nM were done thereafter with the assay buffer. Inhibitor and enzyme solutions were preincubated together for 15 min at room temperature prior to the assay, allowing the formation of the E–I complex. The inhibition constants were obtained by non-linear least-squares methods using PRISM 3 and the Cheng–Prusoff equation as reported earlier, which represent the mean from at least three different determinations. All CA isoforms were recombinant proteins obtained in-house, as reported earlier [62,63,64,65].

### 3.9. Molecular Modeling Studies

AutoDock 4.2 software was used to perform molecular modeling studies [66]. The crystal structures of the cytosolic isoforms hCA I (PDB code 3W6H) and hCA II (PDB code 3HS4), as well as the transmembrane tumor-associated ones hCA IX (PDB code 3IAI) and hCA XII (PDB code 1JD0) were obtained from the Protein Data Bank [67]. The procedure was carried out as mentioned in our previous work [68]. For the preparation of enzymes, all water molecules were removed, polar hydrogens were added and the co-crystallized ligands were removed from each enzyme’s active site. Charges were added and the rotatable bonds determined for preparation of the tested compounds. The autogrid algorithm was used for the calculation of grid maps. A set of grids of 60 Å × 50 Å × 50 Å with 0.375 Å spacing was calculated considering the docking area for all the ligands atom types employing AutoGrid4. Three-dimensional structures of all compounds were constructed using Chem3Dultra 12.0 software (Chemical Structure Drawing Standard; Perkin Elmer Informatics, Waltham, MA, USA). For the present system, the Lamarckian genetic algorithm was applied for minimization using default parameters. The pitch was 1.0 Å, while the quaternion and pivot angle was set to 5.0 degrees. For each compound, 200 configurations were produced. The results from the Autodock calculations were grouped using a root mean standard deviation (RMSD) value of 1.5 Å, while the lowest-energy configuration of the largest population group was chosen as the most likely tethering configuration. The LigandScout software program was used to display the results and process the configurations with the highest tie rating [68]. Finally, the docking protocol was verified by re-docking of the co-crystallized ligand acetazolamide (**AAZ**) in the vicinity of the active sites of each enzyme with RMSD values 0.885, 0.966, 1.034, and 1.176 Å for hCA I, II, IX, and XII, respectively.

### 3.10. In-Silico Predictive Studies

The targeted molecules were appraised for predicting the Drug-likeness based on 5 separate filters namely. Lipinski, Ghose, Veber, Egan, and Muegge [55,56,57,58,59,60] rules accompanying bioavailability and Drug-likeness scores using the Molsoft software and SwissADME program (http://swissadme.ch (accessed on 8 July 2021)) using the ChemAxon’s Marvin JS structure drawing tool.

## 4. Conclusions

In summary, we designed and synthesized fifteen sulfanilamide derivatives bearing different heterocyclic rings (**7**–**17**) adopting different synthetic pathways to increase the potency and selectivity. All the synthesized compounds were screened against four CA isoforms, I, II, IX, and XII showing selectivity toward the isoforms hCA II and hCA XII. In addition, in silico studies are performed on the best derivatives (**7c**, **8a**, **8b**, **11**, and **14**), clarifying the mode of interaction within the hCA II and XII binding sites, illustrating the possible interaction with the active site to justify the selective inhibition activity and prospectively guide the future design of more active and isoform-selective CAIs.

## Figures and Tables

**Table 1:** Inhibition data of human CA isoforms I, II, IX, and XII with compounds **7a-f**, **8a-f, 11,14a-b, 17a-b**, and **AAZ** by a stopped-flow CO_2_ hydrase assay [51].

K_I_ (nM) *				
Cmp	hCA I	hCA II	hCA IX	hCA XII
**7a**	717.9	8.4	103.2	6.9
**7c**	3563	37.5	275.6	4.6
**7e**	858.3	23.8	239.5	19.6
**7f**	878.1	34.3	187.2	37.2
**8a**	664.6	8.8	66.6	30.7
**8b**	269.7	6.8	250.8	5.1
**8c**	399.5	8.9	140.5	7.8
**8d**	656.3	8.7	171.3	8.2
**8f**	699.2	68.1	81.4	8.9
**11**	70.6	6.5	259.8	5.3
**14a**	56.5	6.7	280.5	6.1
**14b**	71.2	7.4	155.2	6.6
**17a**	2925	44.7	320.0	7.6
**17b**	918.5	59.4	252.9	40.2
**AAZ**	250	12.1	25.7	5.7

* Mean from 3 different assays, by a stopped-flow technique (errors were in the range of ±5–10% of the reported values).

**Table 2:** Molecular docking free binding energies (kcal/mol) and interactions of tested compounds on hCA I, II, IX, and XII isoforms.

No	hCA Isoform	Estimated Free Binding Energy (Kcal/mol)	Chelating the Zn (II) Ion	Residues Involved in H-Bond Interactions	Residues Involved in Hydrophobic Interactions
**7c**	hCA I	−5.47	No	-	Ala121, Ala135
	hCA II	−7.11	Yes	Thr199	Ile91
	hCA IX	−6.80	Yes	Thr199	Val121, Thr200
	hCA XII	−11.32	Yes	His67, Gln92, Thr200	Leu198
**8a**	hCA I	−6.88	Yes	Thr199	Ala135, Leu198
	hCA II	−8.95	Yes	His94, Thr199	Ile91, Phe131, Thr200
	hCA IX	−7.91	Yes	Thr199	Val121, Thr200
	hCA XII	−6.42	Yes	-	Val121, Leu198
**8b**	hCA I	−8.17	Yes	Gln92	Ala121, Leu198
	hCA II	−9.24	Yes	His94, Thr199	Val121, Phe131, Leu198
	hCA IX	−6.54	Yes	Thr199	Val121, Leu198
	hCA XII	−9.80	Yes	Gln92, His94, Thr200	Val121, Ala131, Leu198
**11**	hCA I	−9.91	Yes	Gln92, Thr199	Leu131, Ala132, Thr202
	hCA II	−9.24	Yes	His94, Thr200	Val121, Phe131, Leu198
	hCA IX	−6.20	Yes	-	Val121, Val131, Leu198
	hCA XII	−9.78	Yes	His94, Thr200	Val121, Val143, Ala131, Leu198
**14a**	hCA I	−11.02	Yes	His67, Thr199	Ala121, Ala132, Leu198
	hCA II	−9.15	Yes	Asn67, Thr199	Ile191, Val121, Phe131, Leu198, Thr200
	hCA IX	−6.51	Yes	Thr199	Val121, Thr200
	hCA XII	−9.42	Yes	Thr200	Val121, Leu198
**AAZ**	hCA I	−8.28	Yes	Gln92	Leu198, Thr199, His200, Pro201, Trp209
	hCA II	−8.87	Yes	Thr199, Thr200	Val121, Phe131, Leu198, Trp209
	hCA IX	−9.02	Yes	Thr199, Thr200	Val121, Val143, Val131, Leu198, Trp209
	hCA XII	−9.14	Yes	Thr199, Thr200	Val121, Val143, Leu198, Trp209

**Table 3:** Drug likeness predictions and Physicochemical-Pharmacokinetic/ADME properties of tested compounds.

No	MW	Number of HBA ^a^	Number of HBD ^b^	Log *P*_o/w_ (iLOGP) ^c^	Log S ^d^	TPSA ^e^	BBB permeant ^f^	Lipinski, Ghose, Veber, Egan, and Muegge Violations	Bioavailability Score	Drug-Likeness Model Score
**7a**	319.3	5	2	1.30	Moderately soluble	115.71	No	0	0.55	0.57
**7c**	395.4	6	2	1.79	Poorly soluble	115.71	No	0	0.55	0.44
**7e**	429.9	5	2	2.05	Poorly soluble	115.71	No	0	0.55	0.36
**7f**	429.9	5	2	2.26	Poorly soluble	115.71	No	0	0.55	0.97
**8a**	335.4	4	2	1.72	Moderately soluble	130.81	No	0	0.55	0.59
**8b**	349.4	4	2	2.02	Moderately soluble	130.81	No	0	0.55	0.80
**8c**	411.5	4	2	2.17	Poorly soluble	130.81	No	0	0.55	0.47
**8d**	429,5	5	2	1.84	Poorly soluble	130.81	No	0	0.55	0.21
**8f**	445.9	4	2	2.49	Poorly soluble	130.81	No	0	0.55	1.00
**11**	484.9	7	1	2.90	Poorly soluble	157.18	No	0	0.55	-0.58
**14a**	510.6	9	1	3.10	Poorly soluble	175.64	No	1 (MW > 500)	0.55	-0.53
**14b**	36.,4	6	3	1.19	Moderately soluble	135.97	No	0	0.55	0.14
**17a**	376.5	5	2	1.74	Moderately soluble	132.17	No	0	0.55	0.45
**17b**	404.5	5	2	2.50	Poorly soluble	132.17	No	0	0.55	0.37

^a^ number of hydrogen bond acceptors; ^b^ number of hydrogen bond donors; ^c^ lipophilicity; ^d^ Water solubility (SILICOS-IT [S = Soluble]); ^e^ topological polar surface area (Å^2^); ^f^ Blood-Brain Barrier permeant.

## Data Availability

Data is contained within the article.

## Supplementary Materials

The following are available online at https://www.mdpi.com/article/10.3390/ph14080828/s1.
