# Supplementary material for: New Sulfanilamide Derivatives Incorporating Heterocyclic Carboxamide Moieties as Carbonic Anhydrase Inhibitors
Source: Pharmaceuticals (Basel). 2021 Aug 23;14(8):828. doi: 10.3390/ph14080828 (PMC8398262; doi:10.3390/ph14080828)
Supplement: Supplementary file 1 [file pharmaceuticals-14-00828-s001.zip › pharmaceuticals-1326087-supplementary.pdf]

Supplementary Material

Table of Contents

I. ^{1}H, ^{13}C NMR and LCMS Spectra of Products………………………….....2

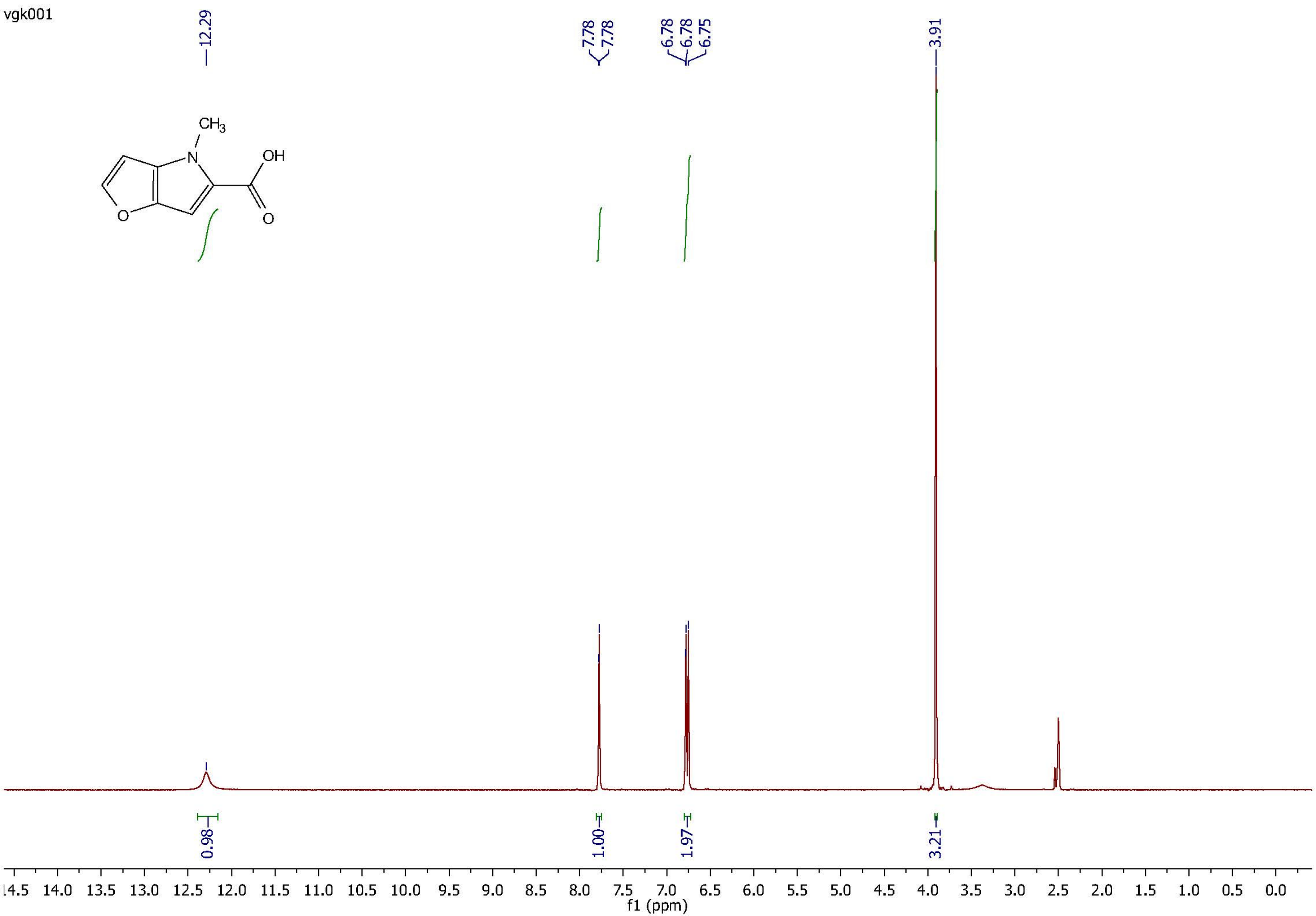

Figure S1. ^{1}H NMR spectrum of *4-Methyl-4H-furo[3,2-b]pyrrole-5-carboxylic acid* (**3a**).

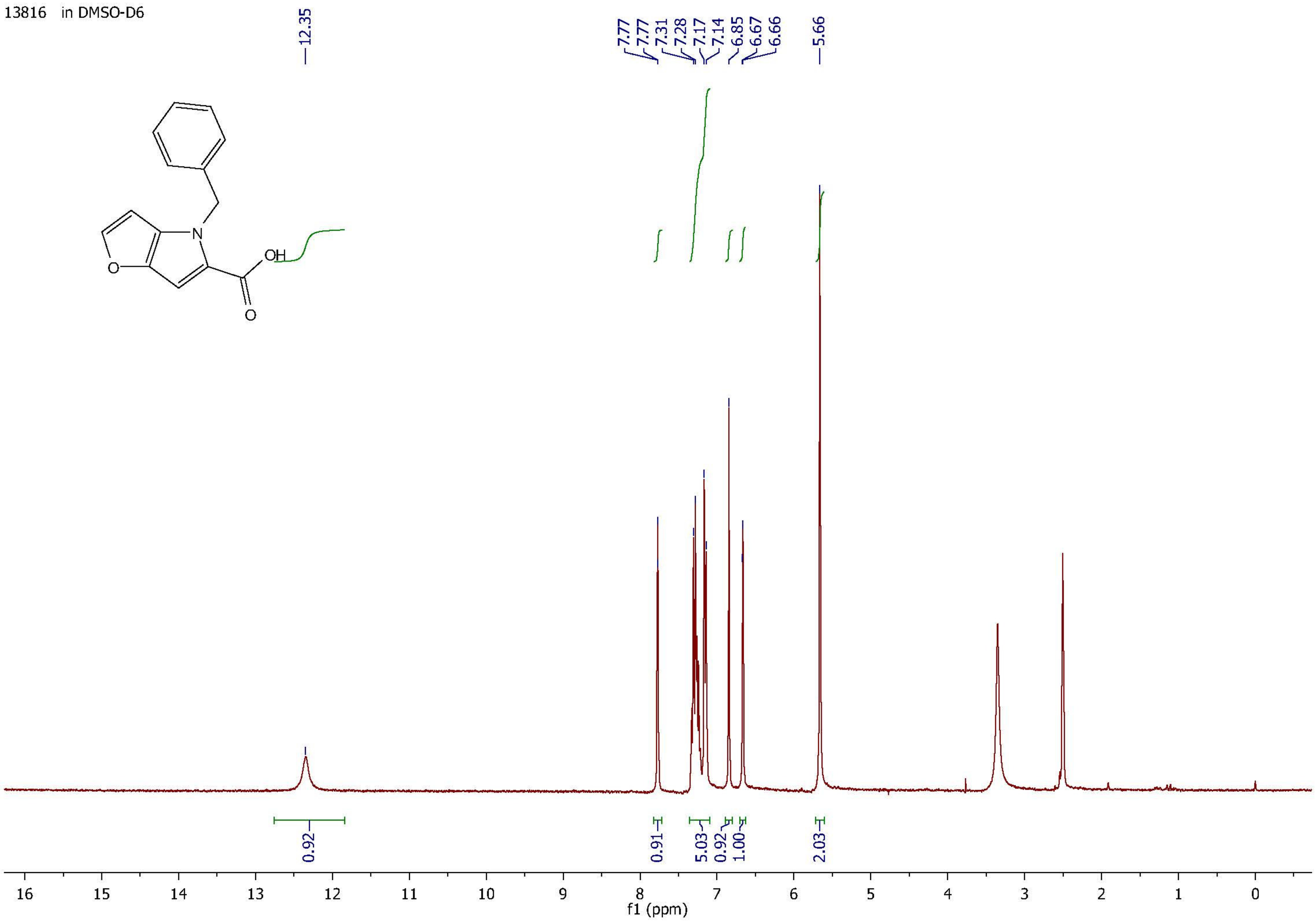

Figure S2. ^{1}H NMR spectrum of *4-Benzyl-4H-furo[3,2-b]pyrrole-5-carboxylic acid* (**3c**).

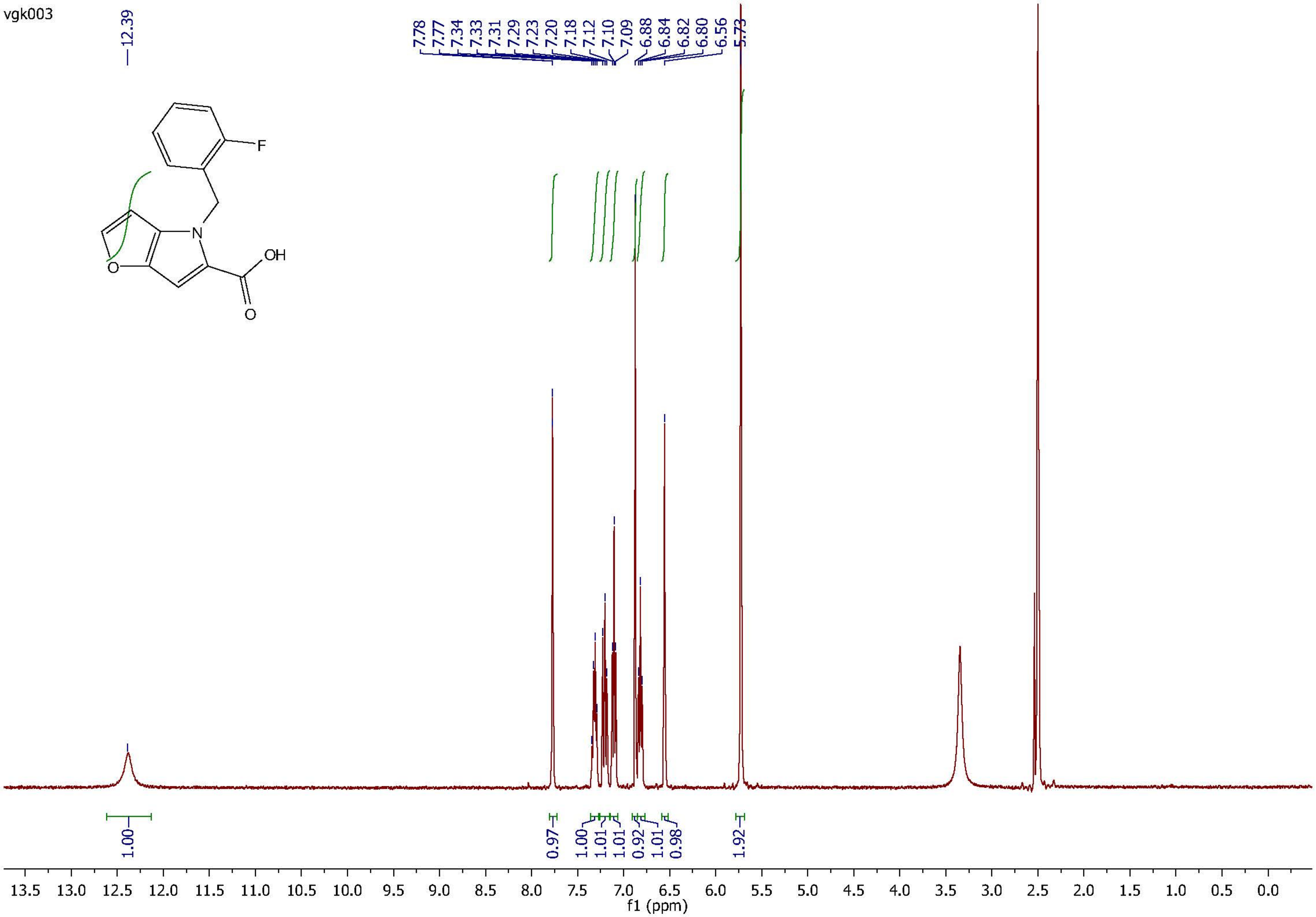

Figure S3. ^{1}H NMR spectrum of *4-(2-Fluorobenzyl)-4H-furo[3,2-b]pyrrole-5-carboxylic acid* (**3d**).

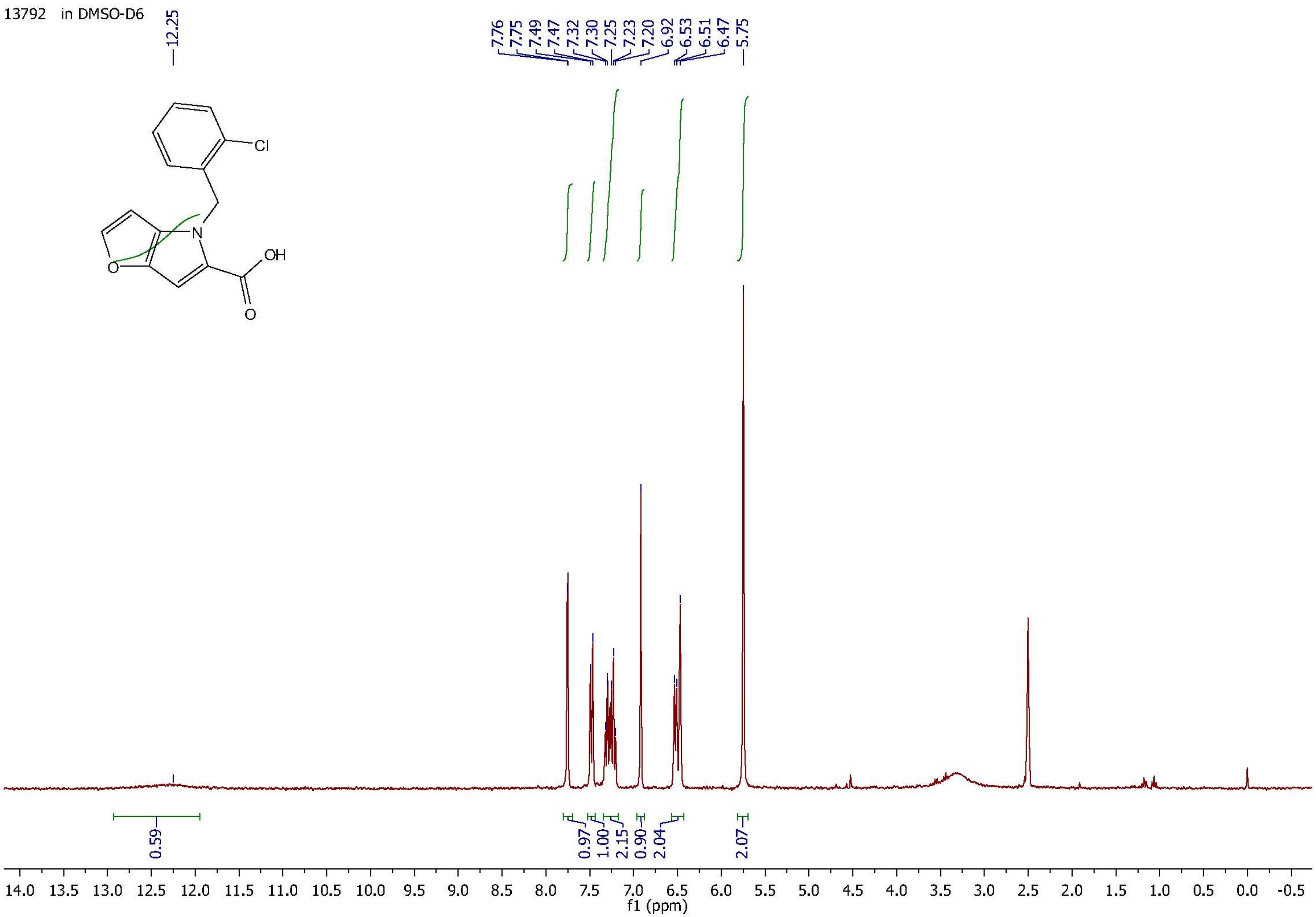

Figure S4. ^{1}H NMR spectrum of *4-(2-Chlorobenzyl)-4H-furo[3,2-b]pyrrole-5-carboxylic acid* (**3e**).

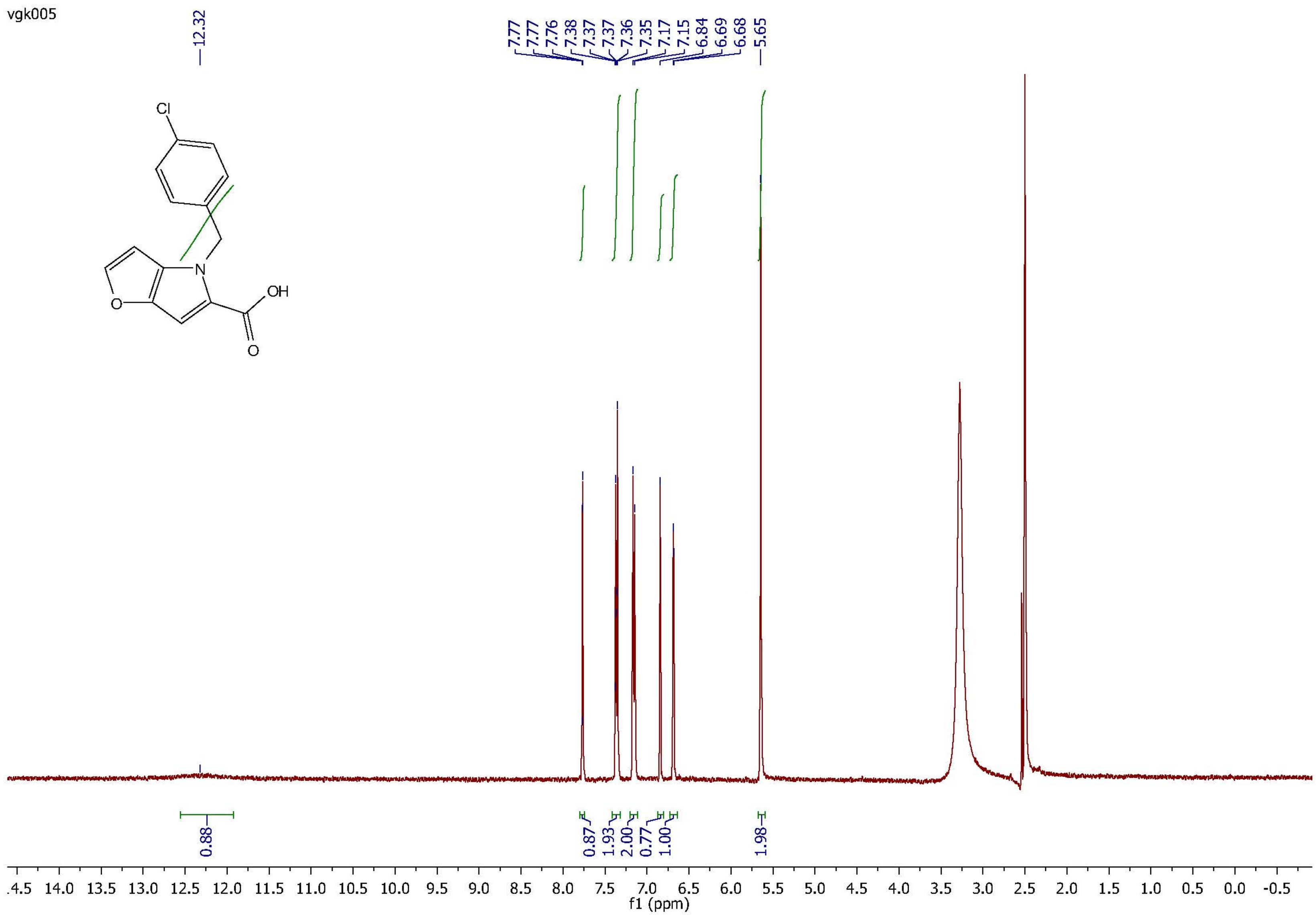

Figure S5. ^{1}H NMR spectrum of *4-(4-Chlorobenzyl)-4H-furo[3,2-b]pyrrole-5-carboxylic acid* (**3f**).

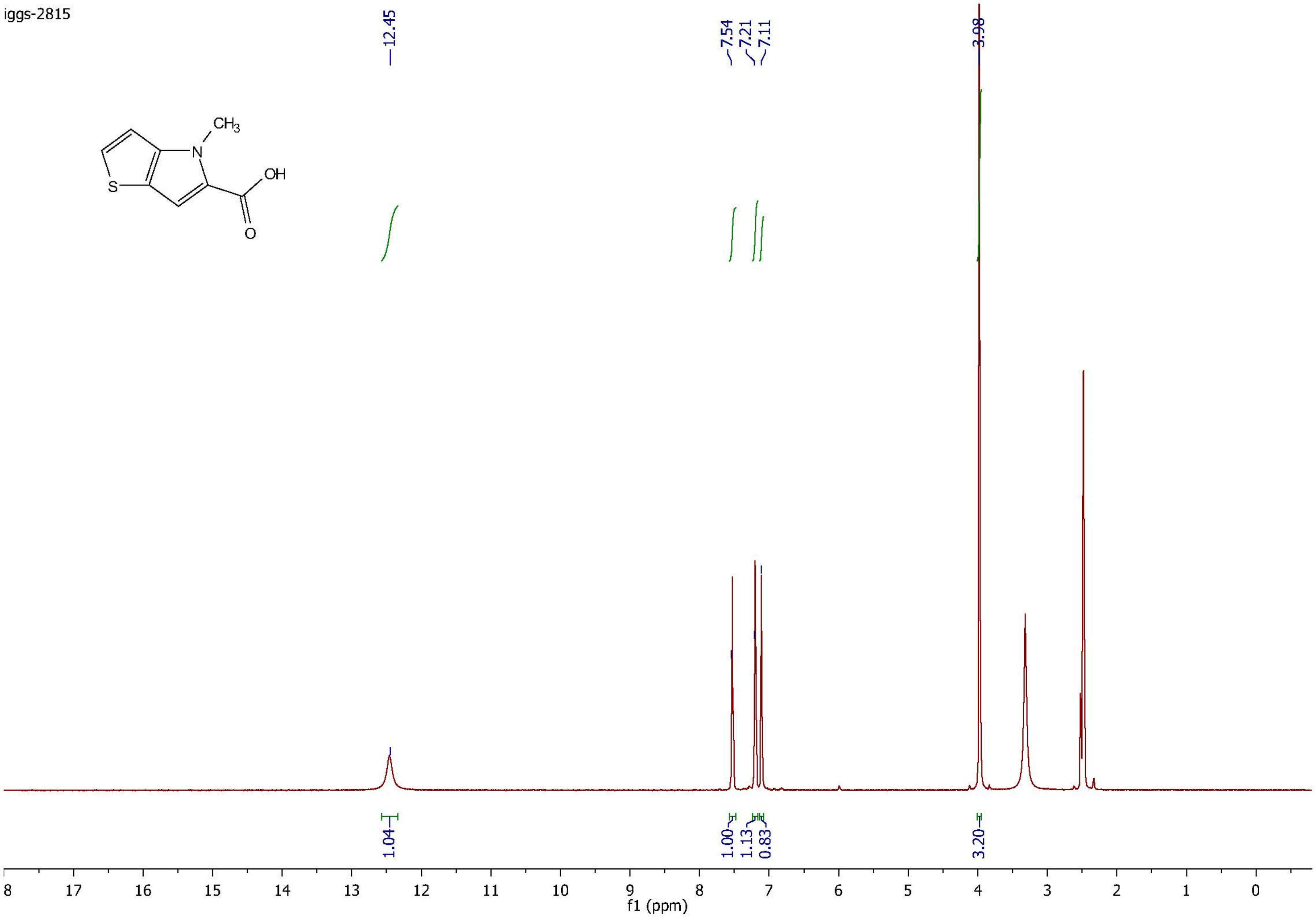

Figure S6. ¹H NMR spectrum of *4-Methyl-4H-thieno[3,2-b]pyrrole-5-carboxylic acid* (**4a**).

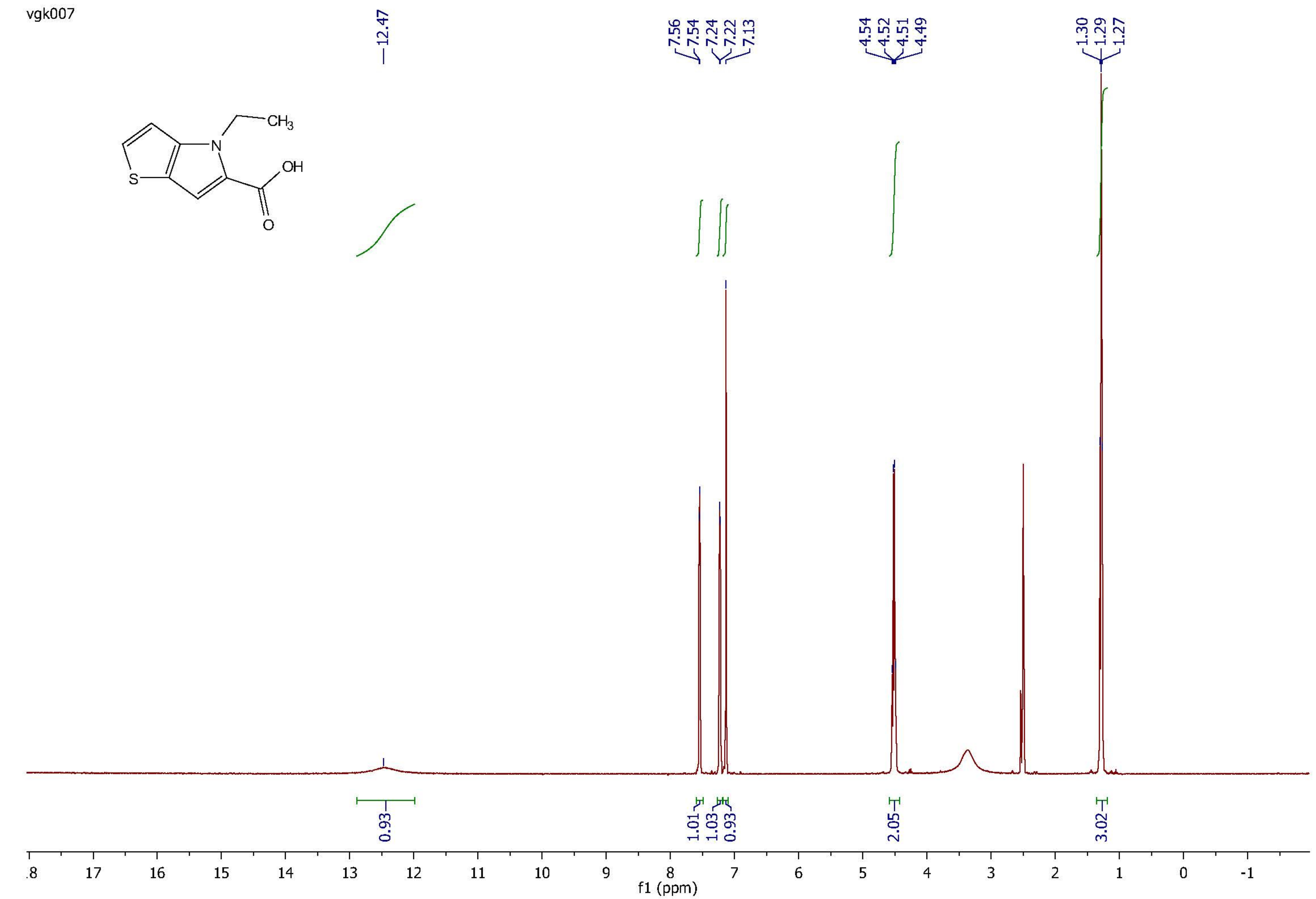

Figure S7. ^{1}H NMR spectrum of *4-Ethyl-4H-thieno[3,2-b]pyrrole-5-carboxylic acid* (**4b**).

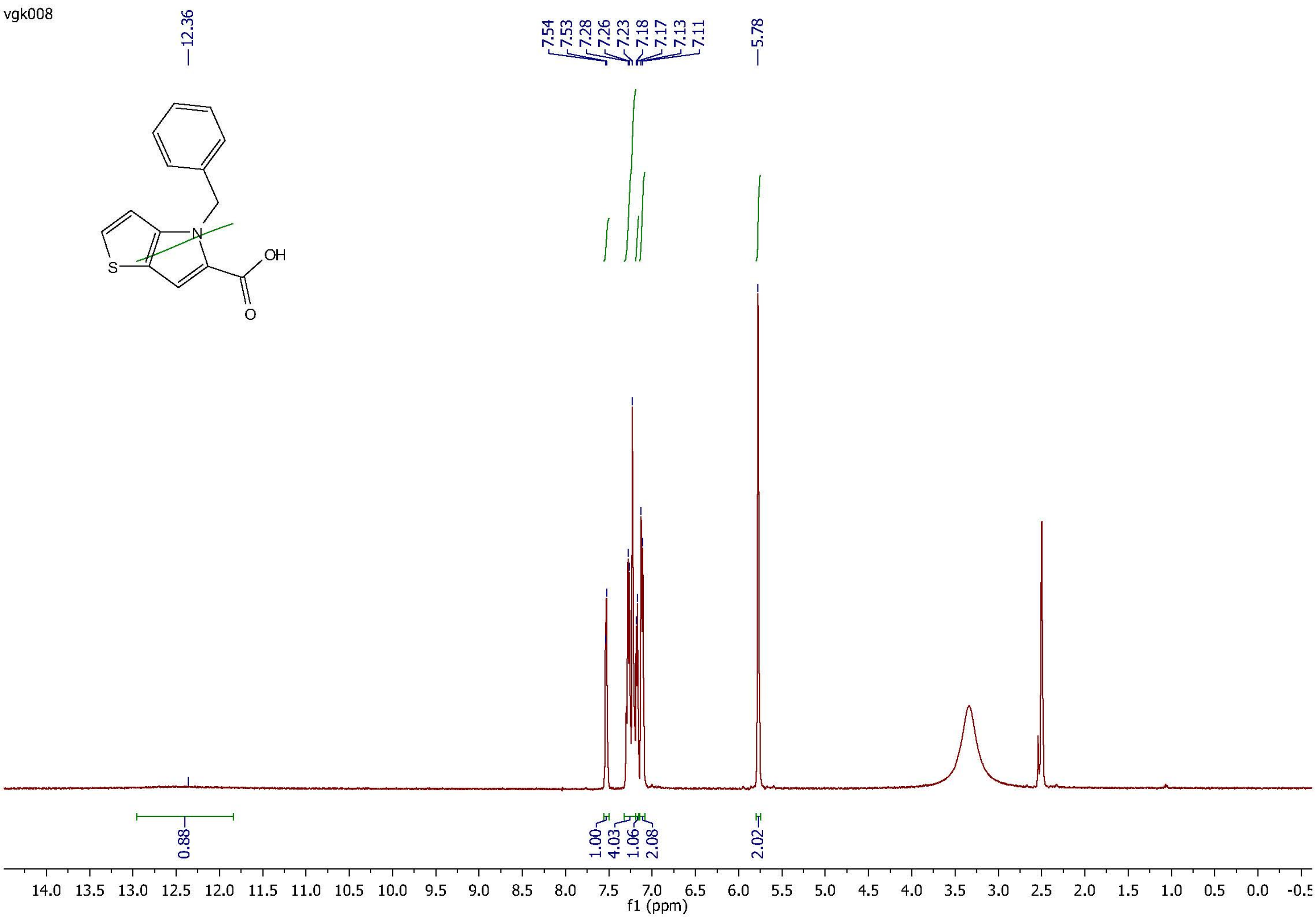

Figure S8. ^{1}H NMR spectrum of *4-Benzyl-4H-thieno[3,2-b]pyrrole-5-carboxylic acid* (**4c**).

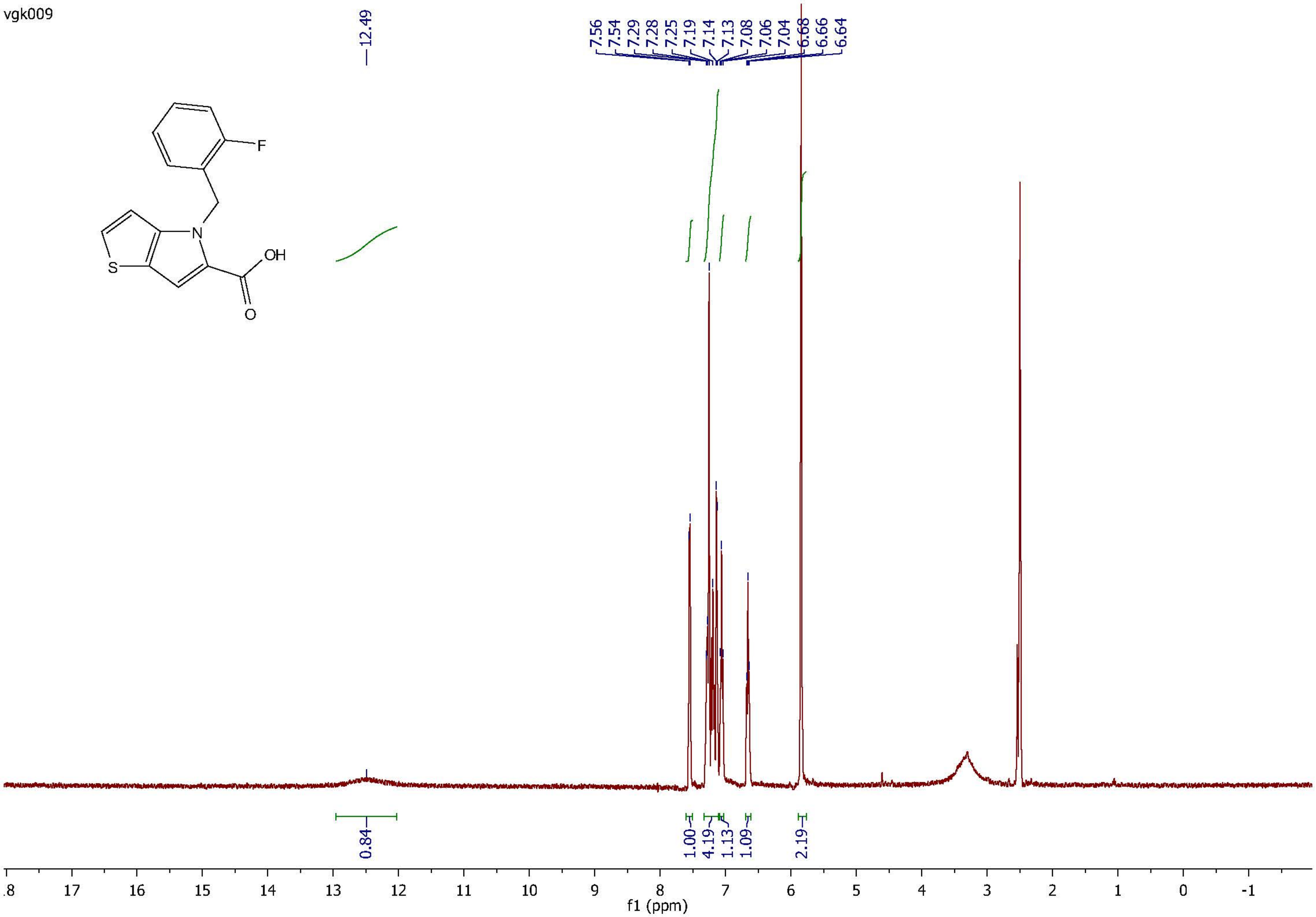

Figure S9. ^{1}H NMR spectrum of *4-(2-Fluorobenzyl)-4H-thieno[3,2-b]pyrrole-5-carboxylic acid* (**4d**).

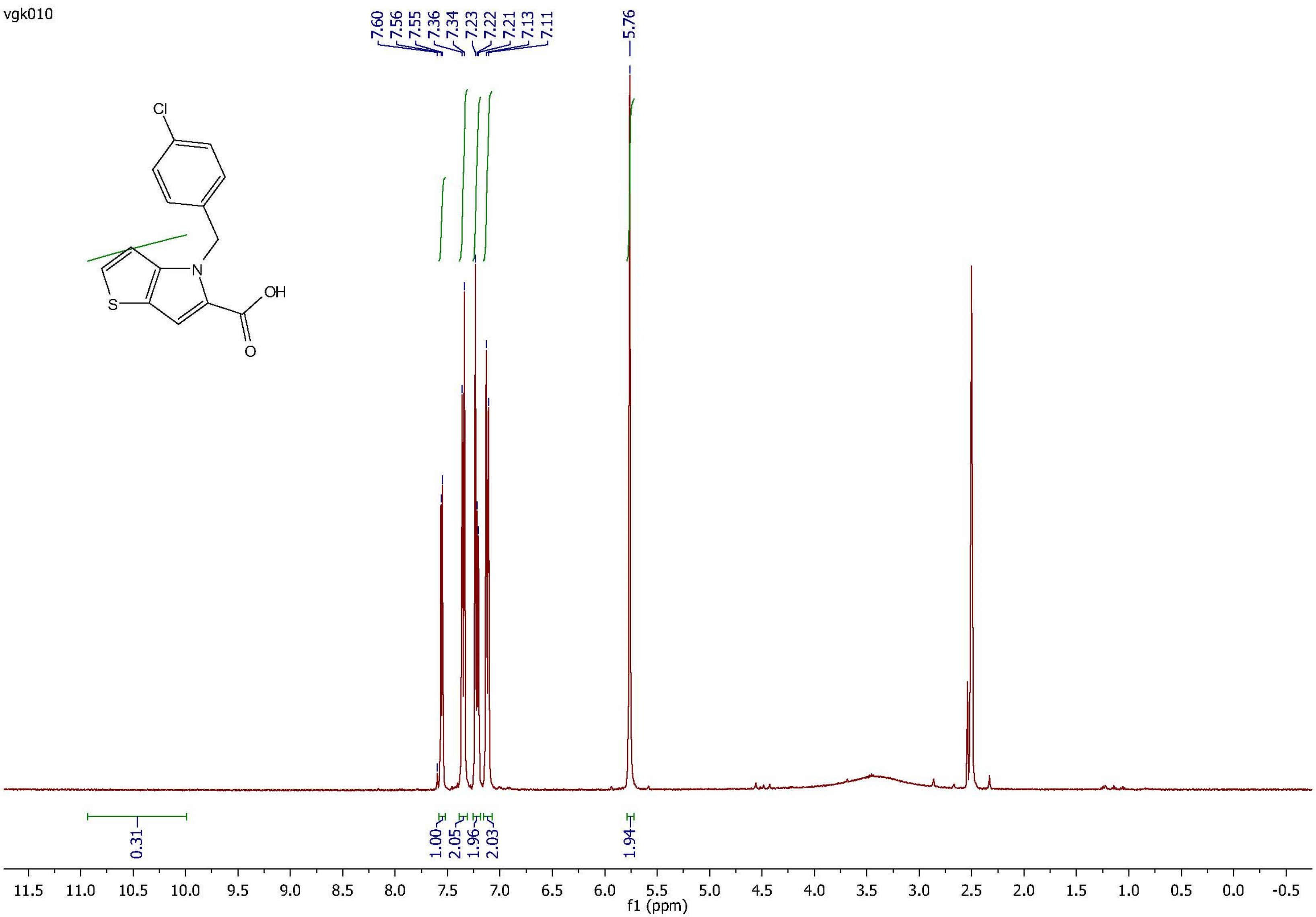

Figure S10. ^{1}H NMR spectrum of *4-(4-Chlorobenzyl)-4H-thieno[3,2-b]pyrrole-5-carboxylic acid* (**4f**).

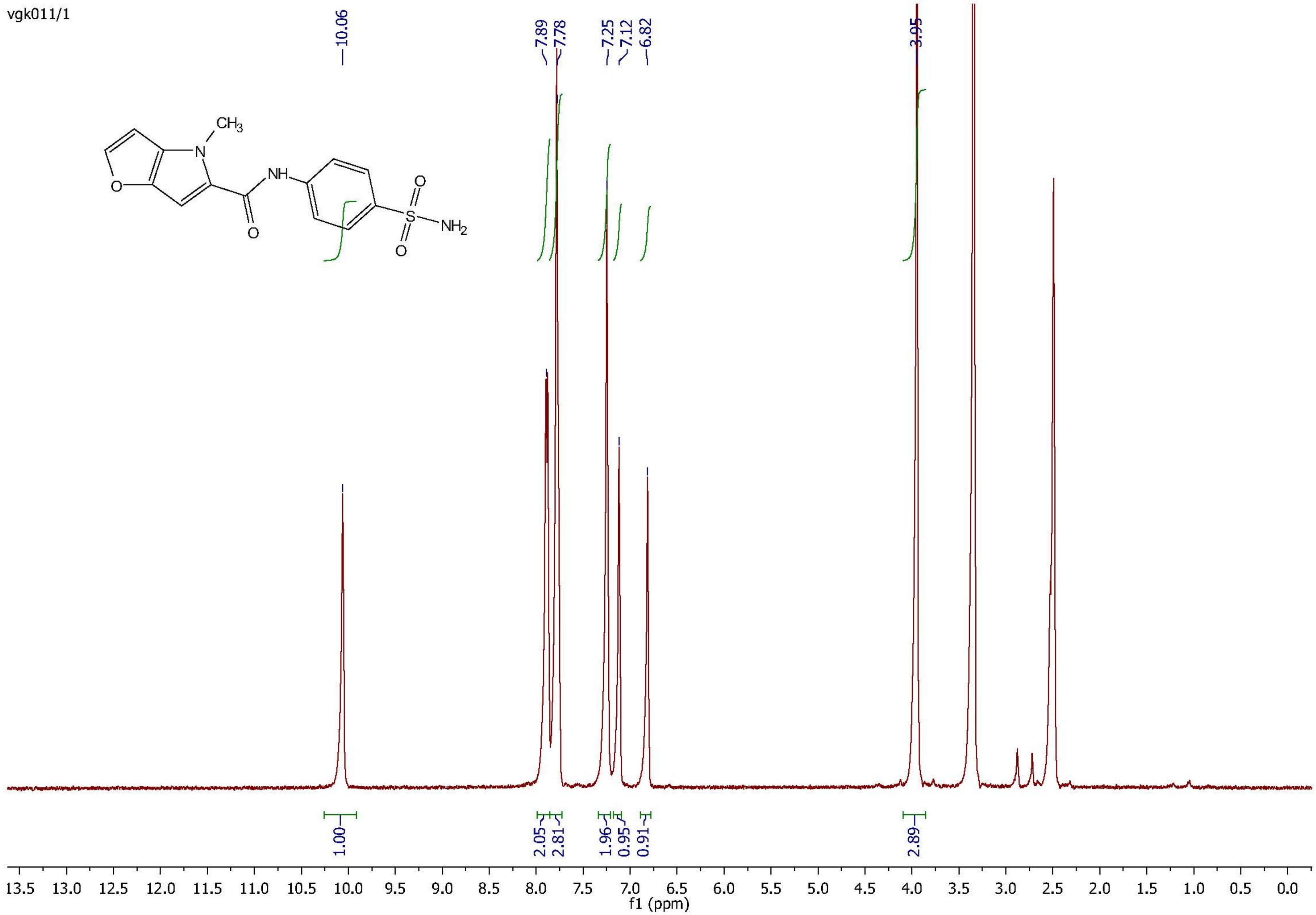

Figure S11. ^{1}H NMR spectrum of *N-[4-(Aminosulfonyl)phenyl]-4-methyl-4H-furo[3,2-b]pyrrole-5-carboxamide* (**7a**).

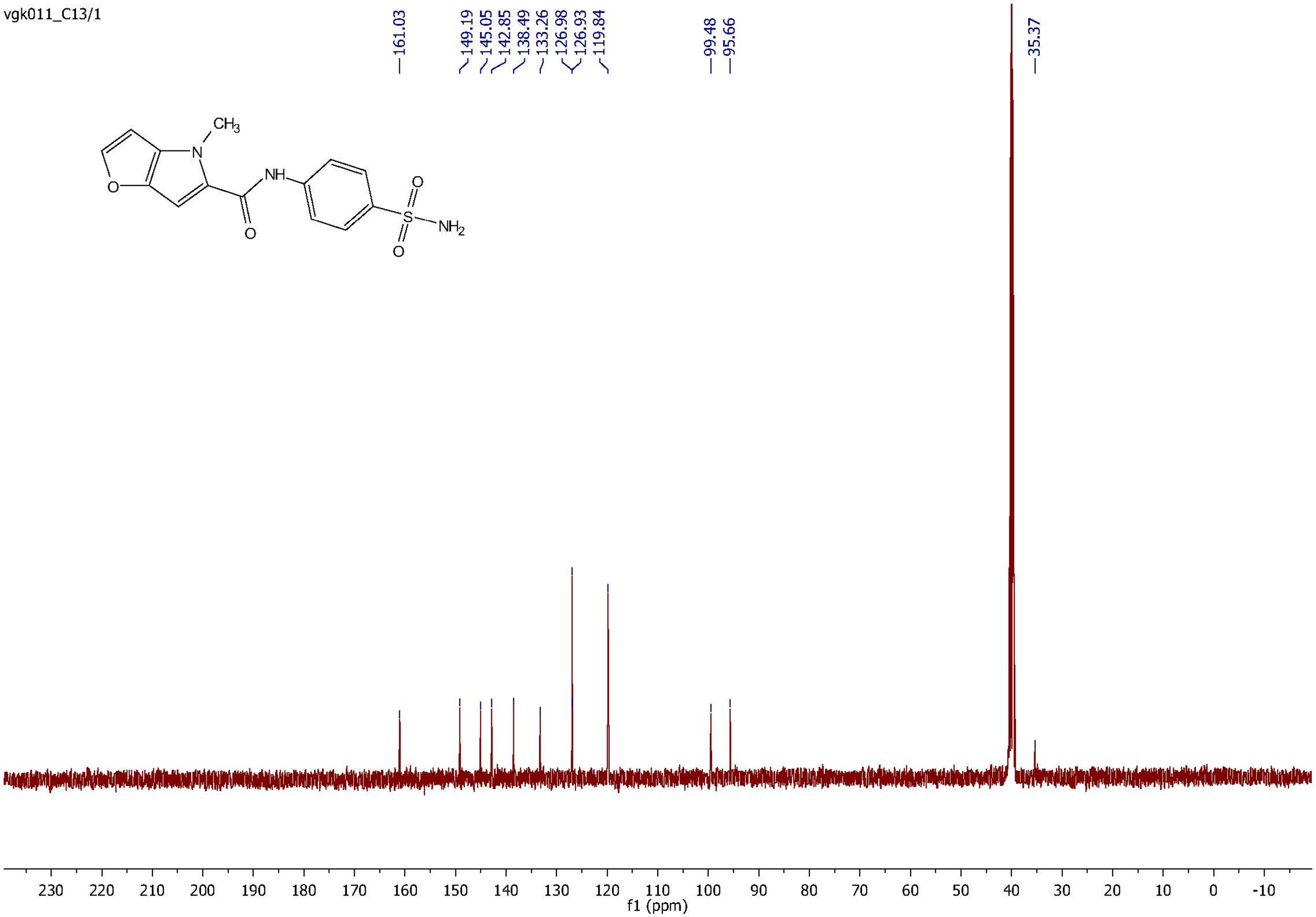

Figure S12. ^{13}C NMR spectrum of *N-[4-(Aminosulfonyl)phenyl]-4-methyl-4H-furo[3,2-b]pyrrole-5-carboxamide* (**7a**).

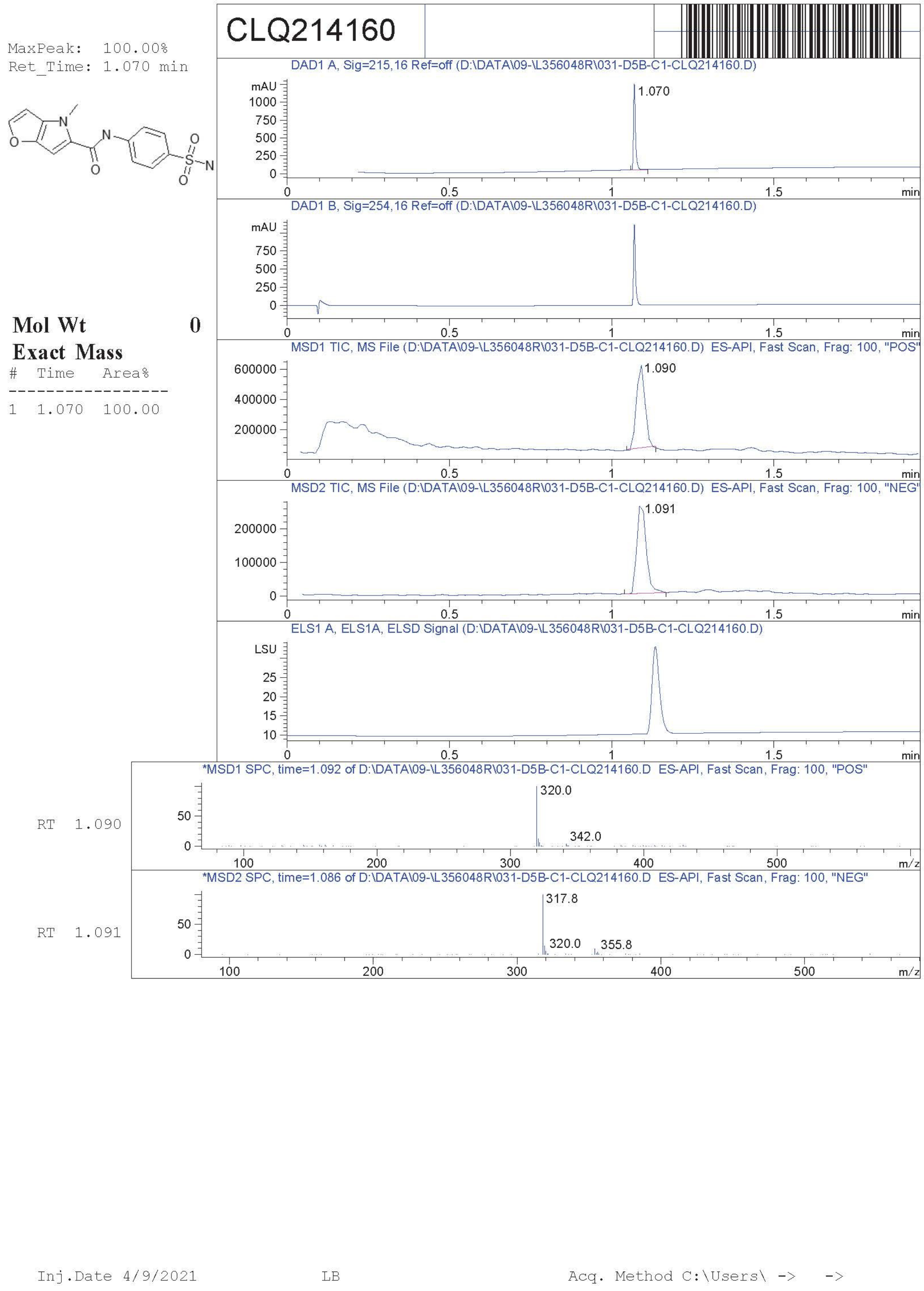

Figure S13. LCMS spectrum of *N-[4-(Aminosulfonyl)phenyl]-4-methyl-4H-furo[3,2-b]pyrrole-5-carboxamide* (**7a**).

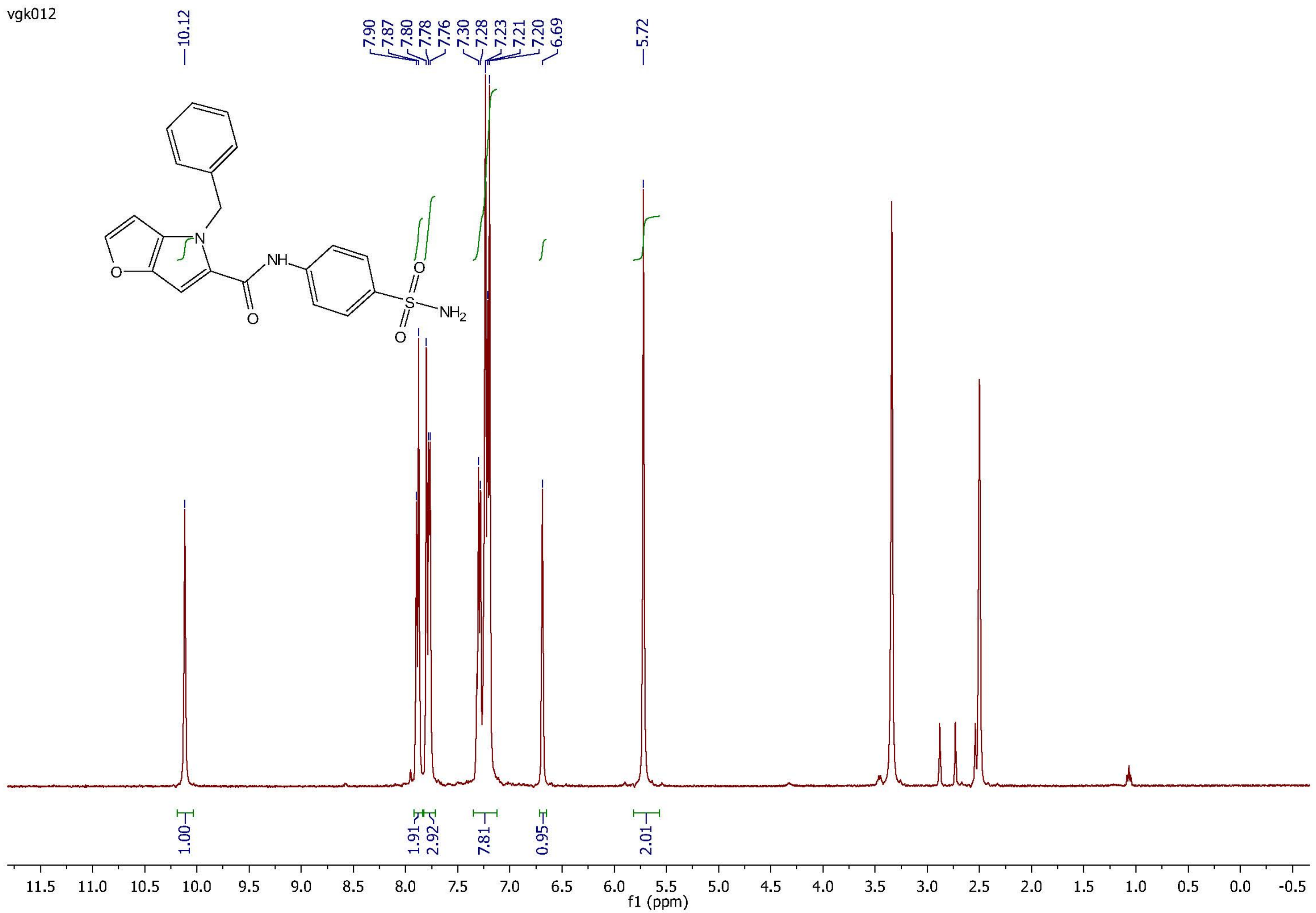

Figure S14. ^{1}H NMR spectrum of *N-[4-(Aminosulfonyl)phenyl]-4-benzyl-4H-furo[3,2-b]pyrrole-5-carboxamide* (**7c**).

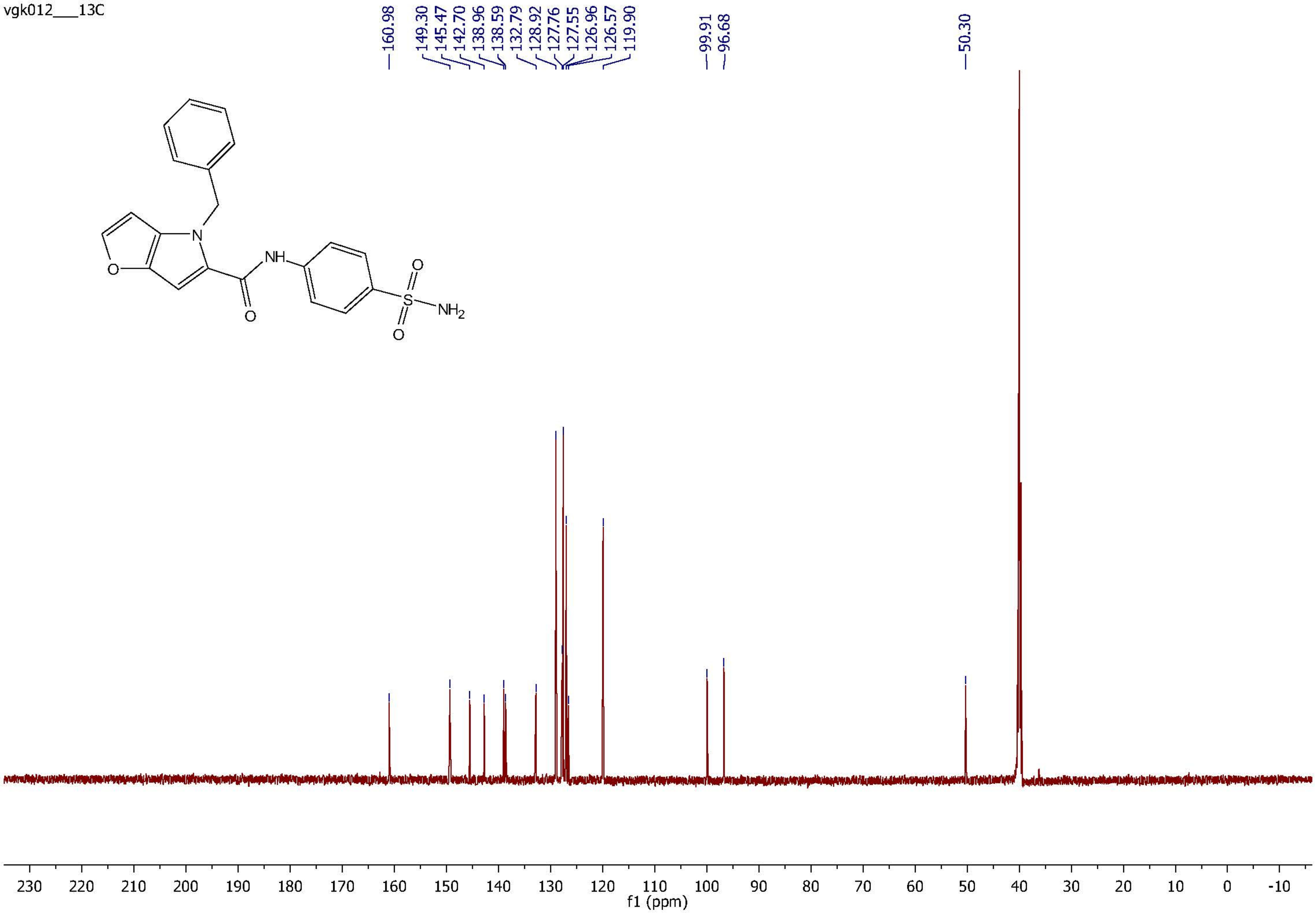

Figure S15. ^{13}C NMR spectrum of *N-[4-(Aminosulfonyl)phenyl]-4-benzyl-4H-furo[3,2-b]pyrrole-5-carboxamide* (**7c**).

MaxPeak: 93.29%
Ret_Time: 1.308 min

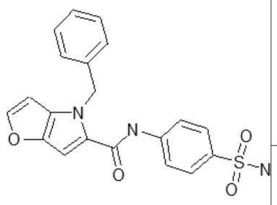

Mol Wt 0
Exact Mass

#	Time	Area%
1	1.240	6.71
2	1.308	93.29

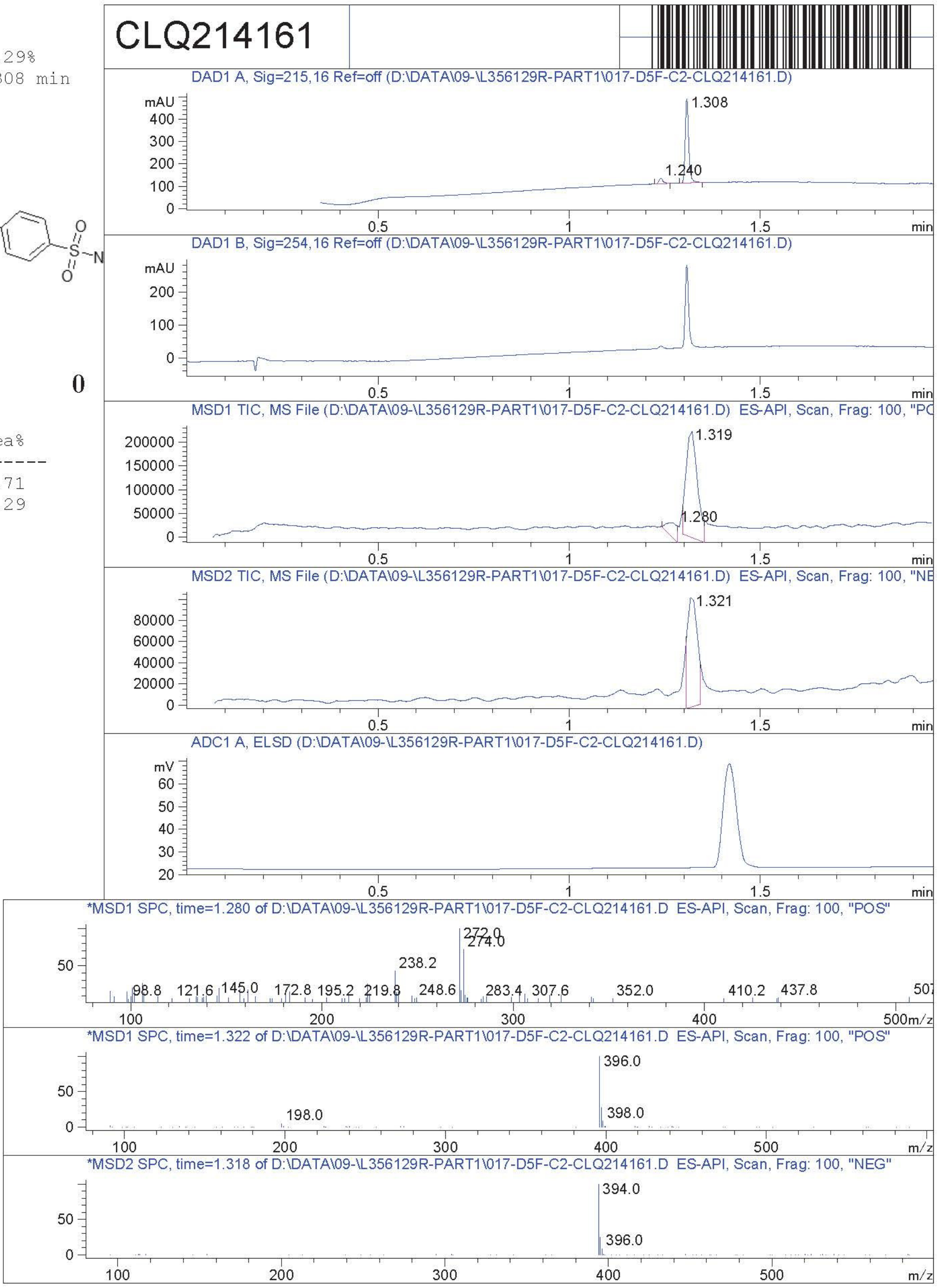

RT 1.280
RT 1.319
RT 1.321

Inj.Date 4/9/2021 LB -12- Acq. Method C:\Chem32\-> ->

Figure S16. LCMS spectrum of *N-[4-(Aminosulfonyl)phenyl]-4-benzyl-4H-furo[3,2-b]pyrrole-5-carboxamide* (**7c**).

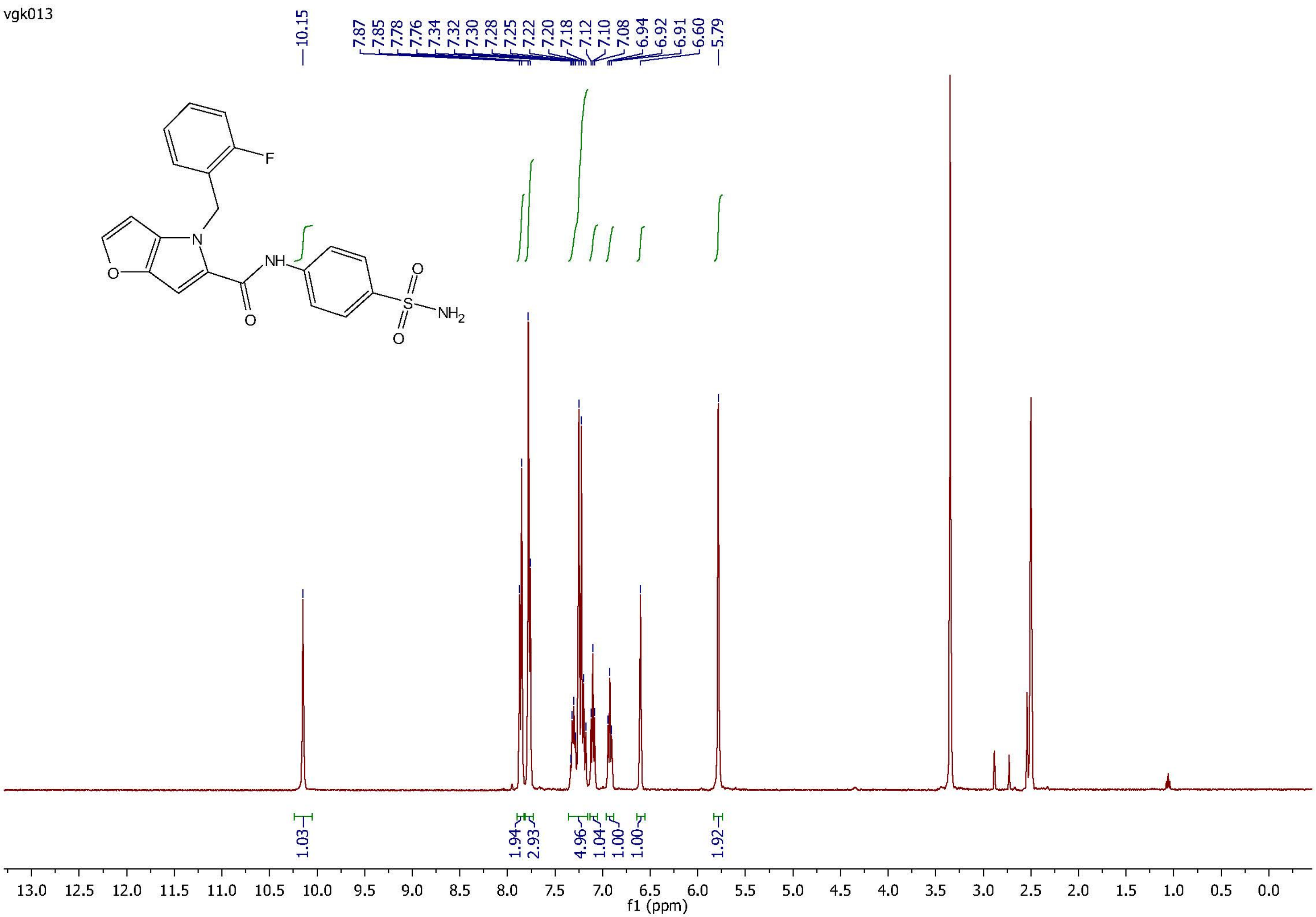

Figure S17. ^{1}H NMR spectrum of *N-[4-(Aminosulfonyl)phenyl]-4-(2-fluorobenzyl)-4H-furo[3,2-b]pyrrole-5-carboxamide* (**7d**).

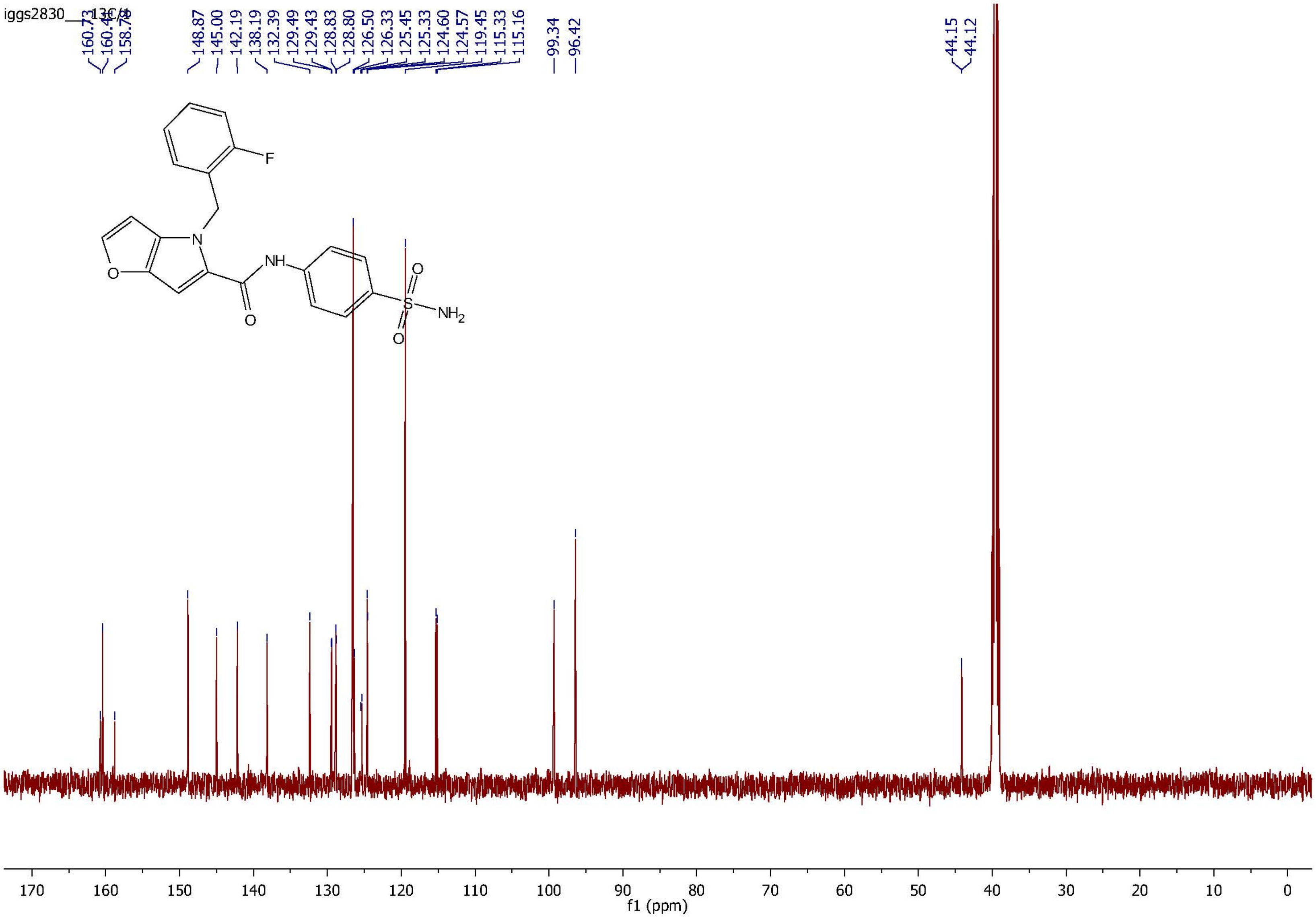

Figure S18. ^{13}C NMR spectrum of *N-[4-(Aminosulfonyl)phenyl]-4-(2-fluorobenzyl)-4H-furo[3,2-b]pyrrole-5-carboxamide* (**7d**).

MaxPeak: 100.00%
Ret_Time: 1.319 min

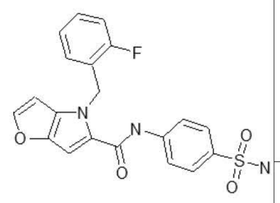

Mol Wt 0

Exact Mass

#	Time	Area%
1	1.319	100.00

RT 1.340

RT 1.341

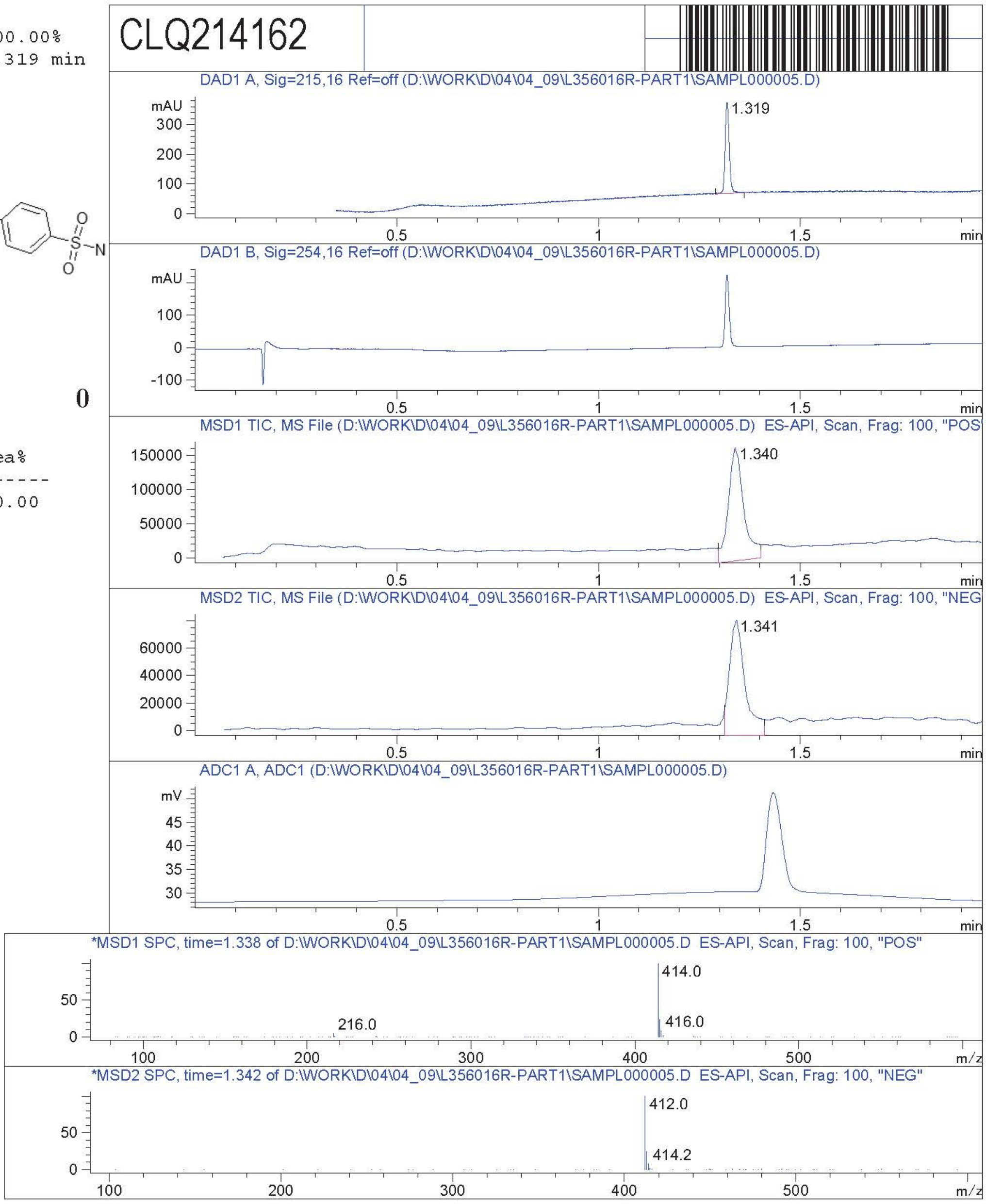

Inj.Date 4/9/2021 K - 4 - Acq. Method C:\CHEM32\-> ->

Figure S19. LCMS spectrum of *N-[4-(Aminosulfonyl)phenyl]-4-(2-fluorobenzyl)-4H-furo[3,2-b]pyrrole-5-carboxamide* (**7d**).

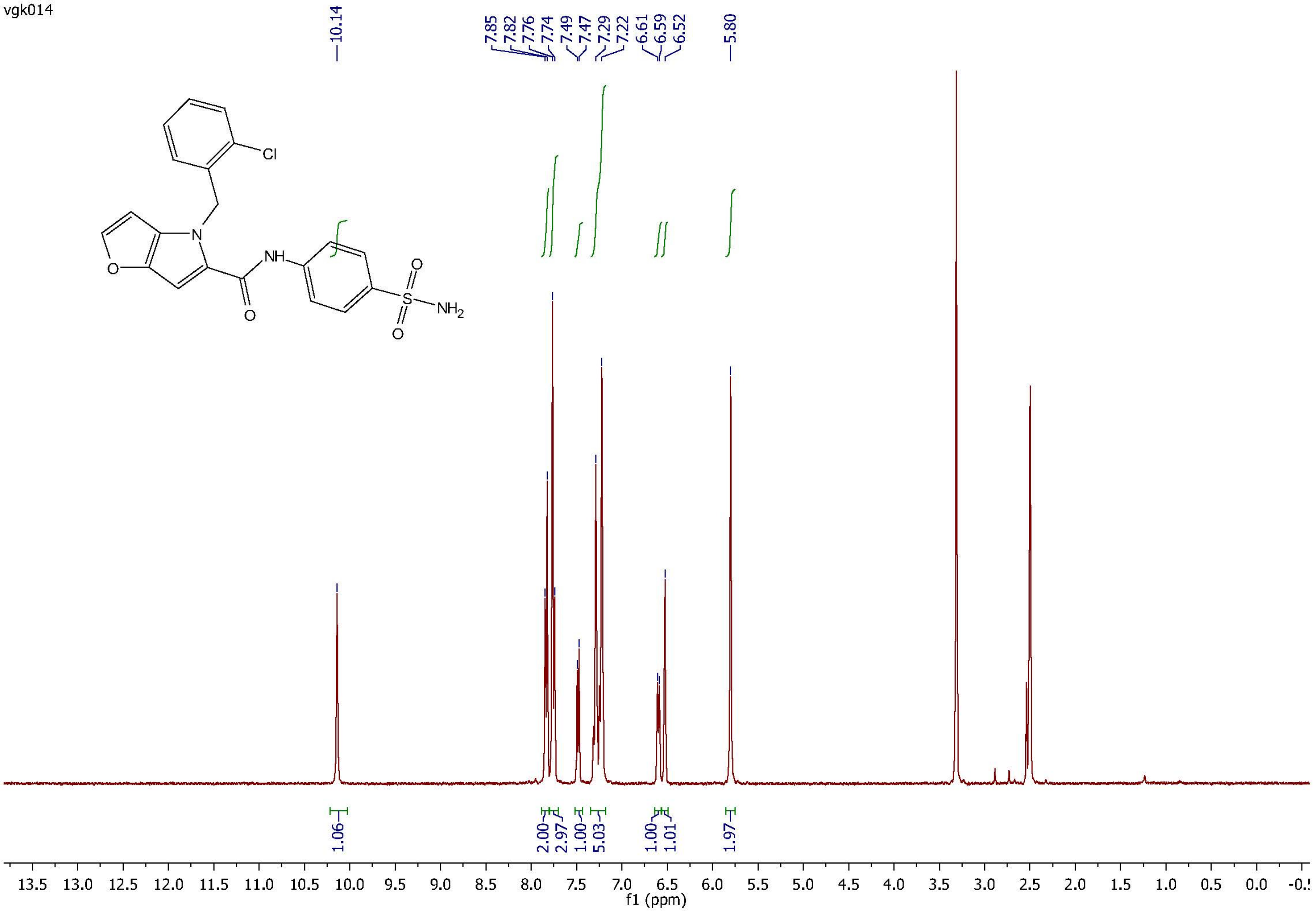

Figure S20. ^{1}H NMR spectrum of *N-[4-(Aminosulfonyl)phenyl]-4-(2-chlorobenzyl)-4H-furo[3,2-b]pyrrole-5-carboxamide* (**7e**).

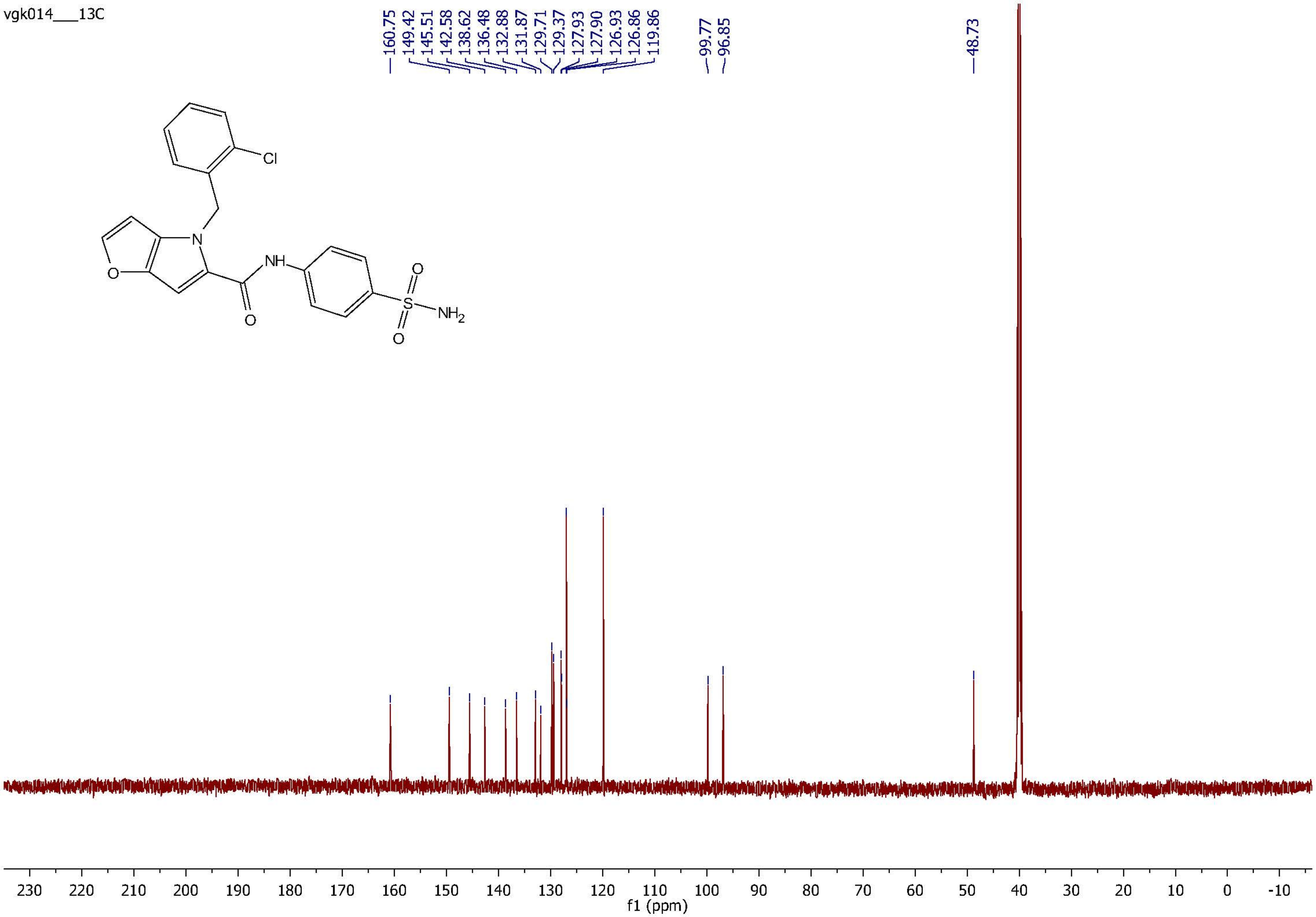

Figure S21. ^{13}C NMR spectrum of *N-[4-(Aminosulfonyl)phenyl]-4-(2-chlorobenzyl)-4H-furo[3,2-b]pyrrole-5-carboxamide* (**7e**).

MaxPeak: 100.00%
Ret_Time: 1.363 min

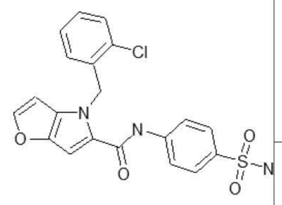

Mol Wt 0
Exact Mass

#	Time	Area%
1	1.363	100.00

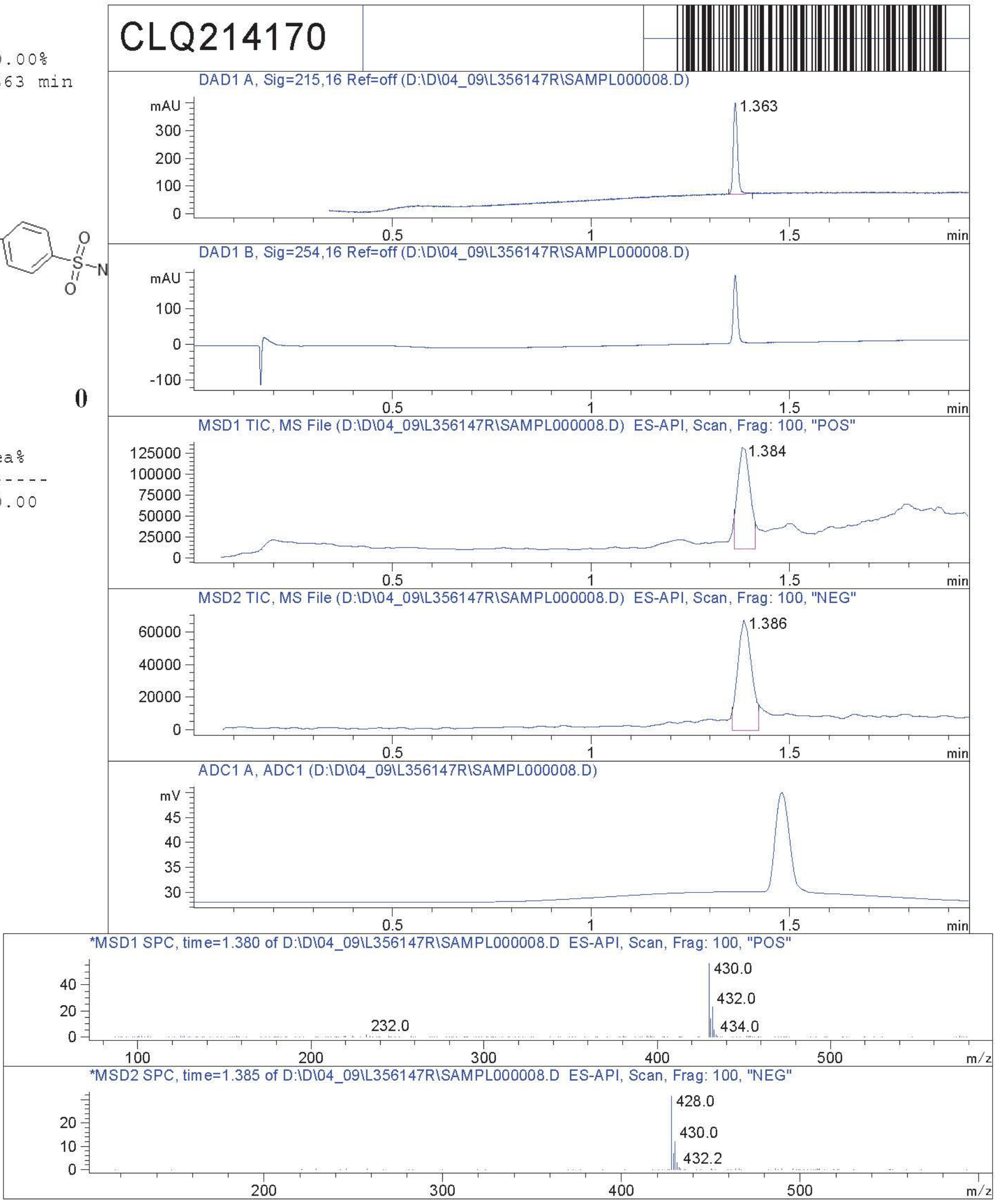

RT 1.384

RT 1.386

Inj.Date 4/9/2021 N - 4 - Acq. Method C:\CHEM32\-> ->

Figure S22. LCMS spectrum of *N-[4-(Aminosulfonyl)phenyl]-4-(2-chlorobenzyl)-4H-furo[3,2-b]pyrrole-5-carboxamide* (**7e**).

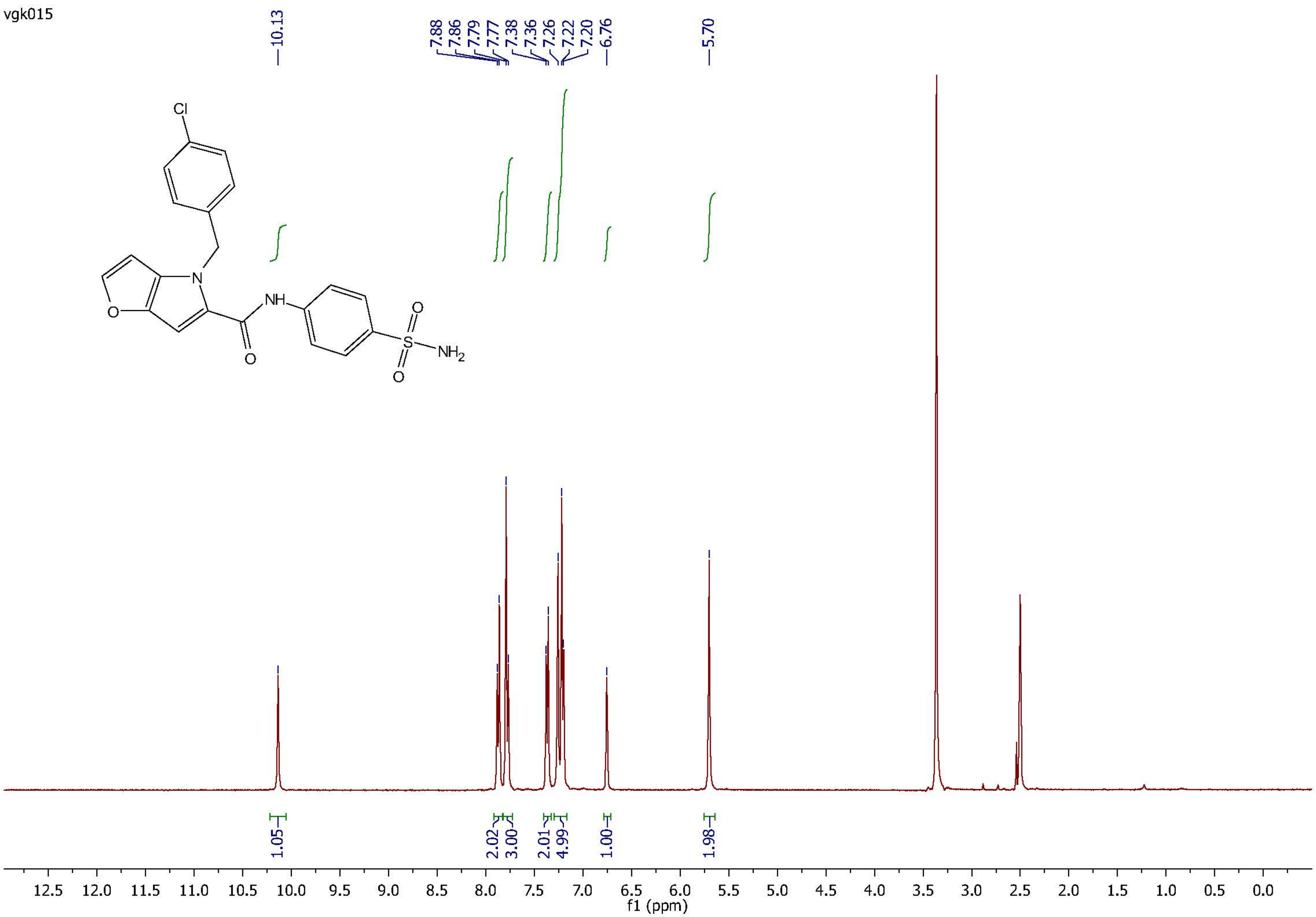

Figure S23. ^{1}H NMR spectrum of *N-[4-(Aminosulfonyl)phenyl]-4-(4-chlorobenzyl)-4H-furo[3,2-b]pyrrole-5-carboxamide* (**7f**).

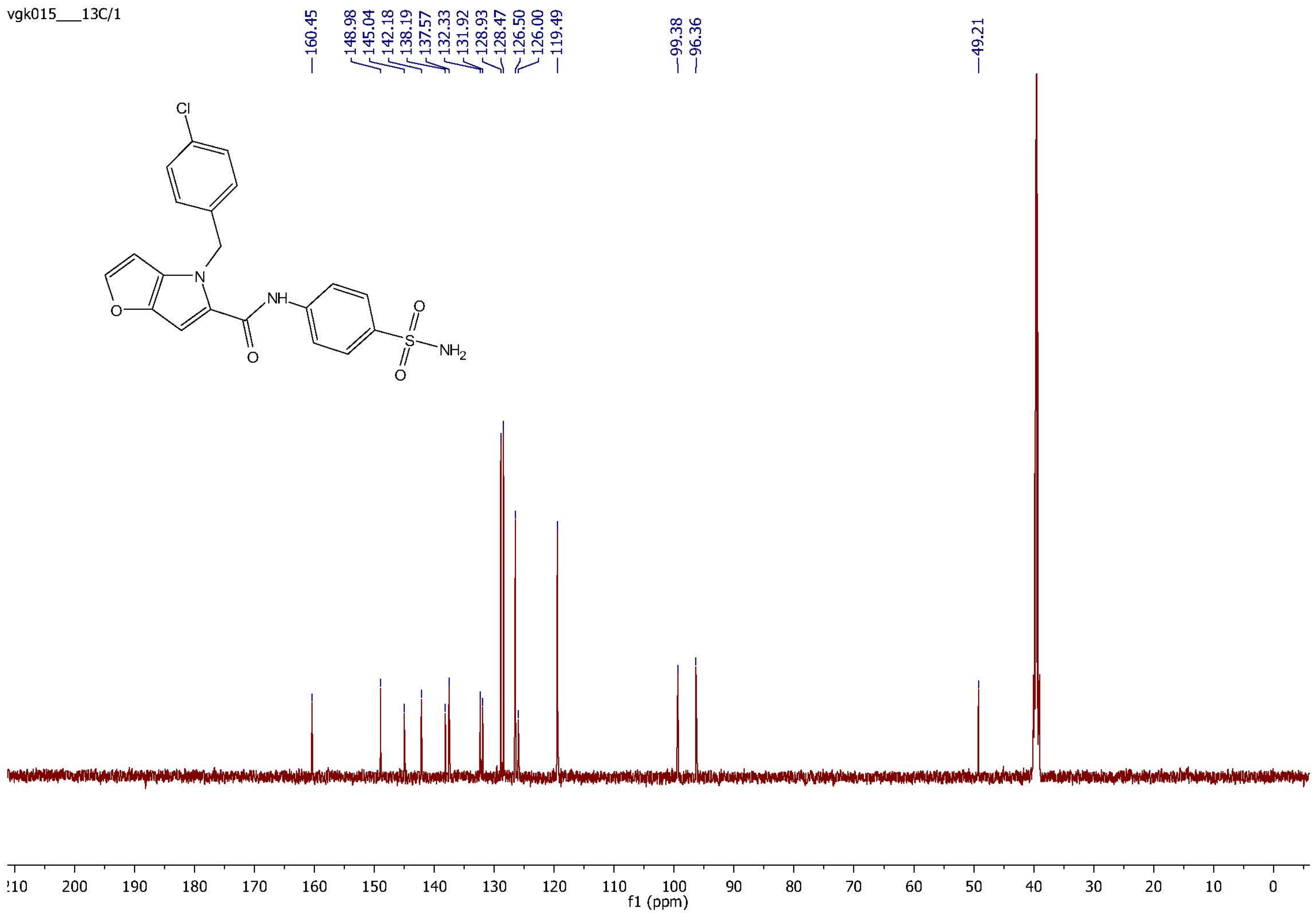

Figure S24. ^{13}C NMR spectrum of *N-[4-(Aminosulfonyl)phenyl]-4-(4-chlorobenzyl)-4H-furo[3,2-b]pyrrole-5-carboxamide* (**7f**).

MaxPeak: 100.00%
Ret_Time: 1.363 min

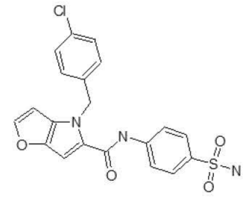

Mol Wt 0
Exact Mass

#	Time	Area%
1	1.363	100.00

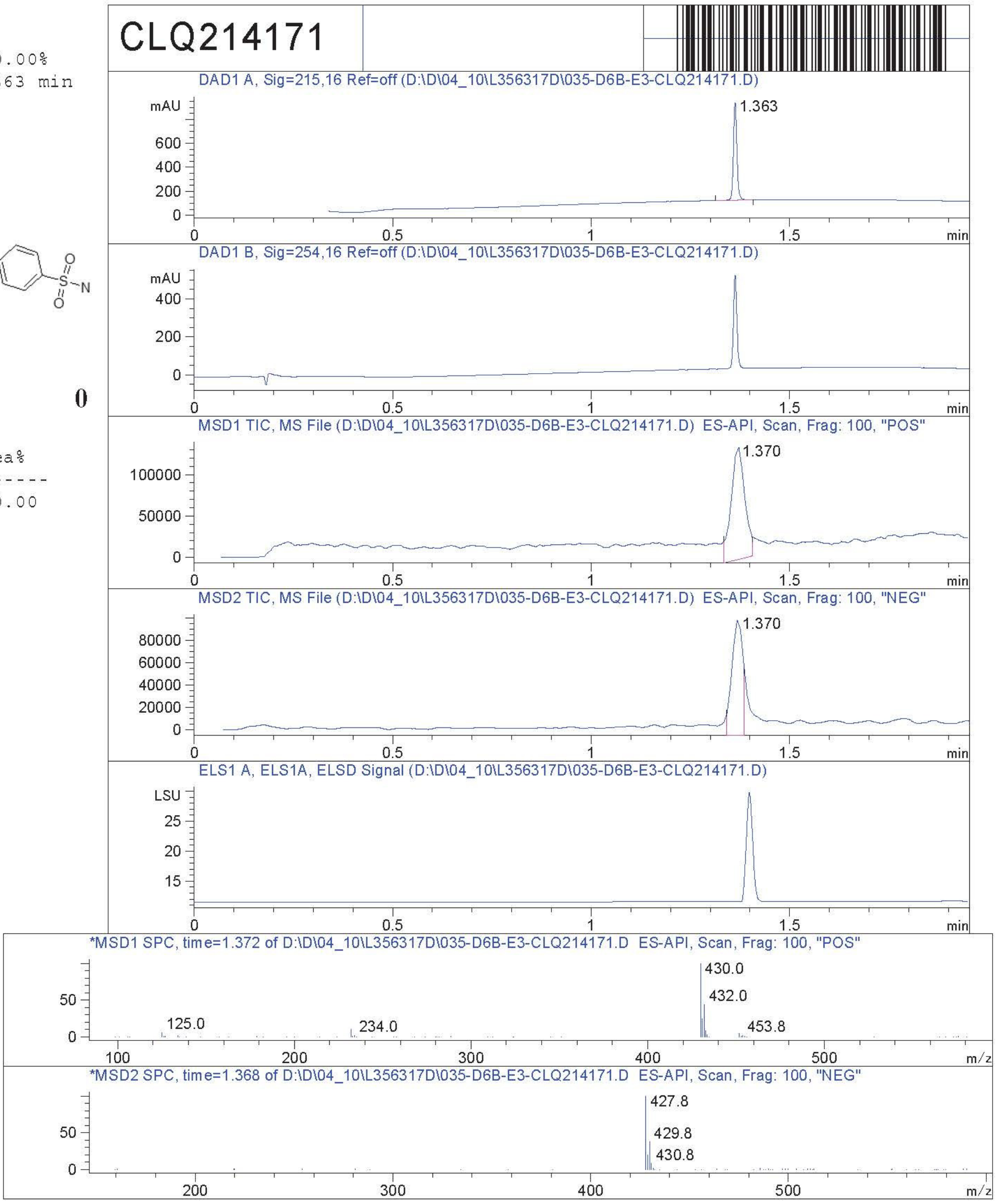

RT 1.370

RT 1.370

Figure S25. LCMS spectrum of *N-[4-(Aminosulfonyl)phenyl]-4-(4-chlorobenzyl)-4H-furo[3,2-b]pyrrole-5-carboxamide* (**7f**).

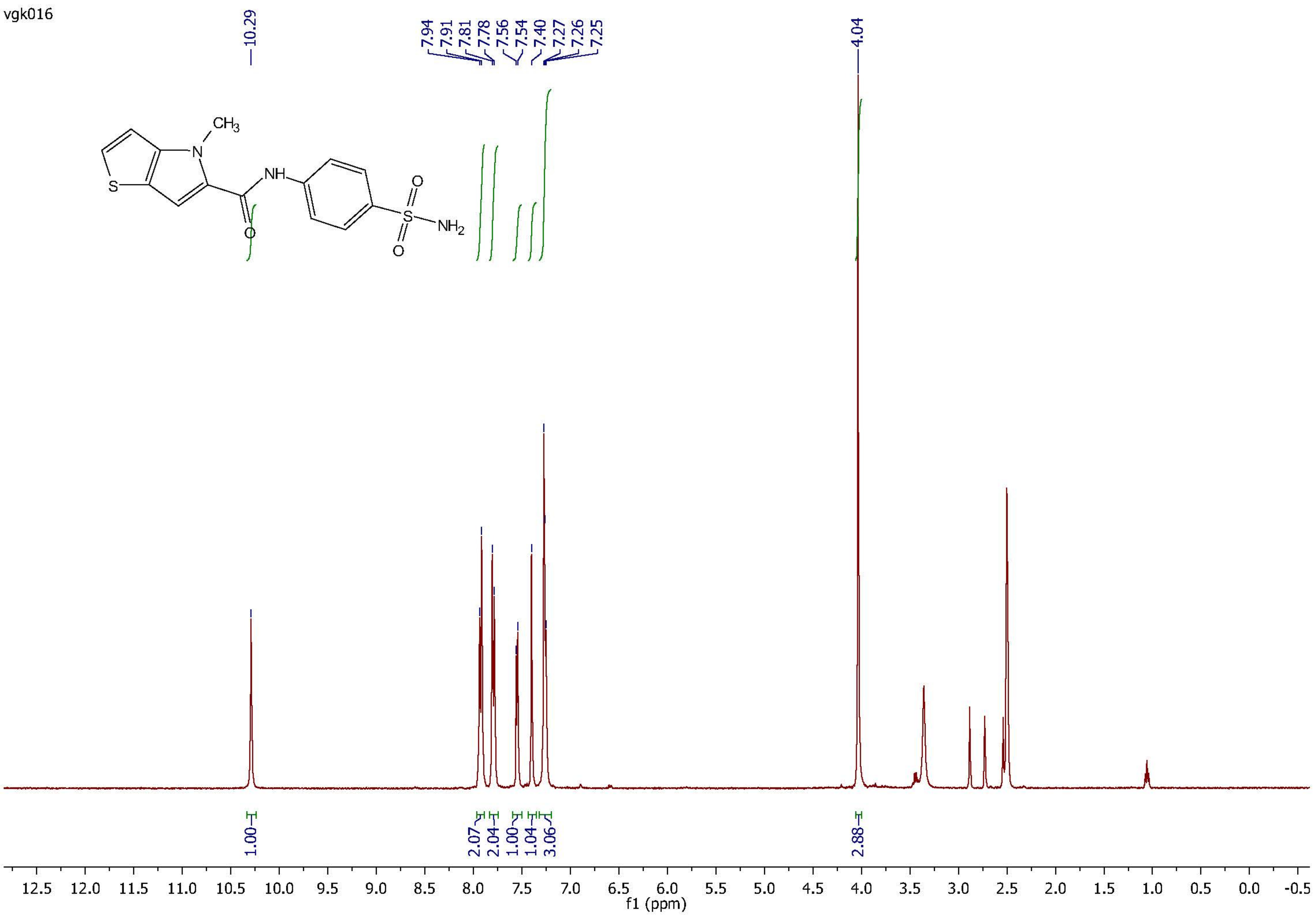

Figure S26. ^{1}H NMR spectrum of *N-[4-(Aminosulfonyl)phenyl]-4-methyl-4H-thieno[3,2-b]pyrrole-5-carboxamide* (**8a**).

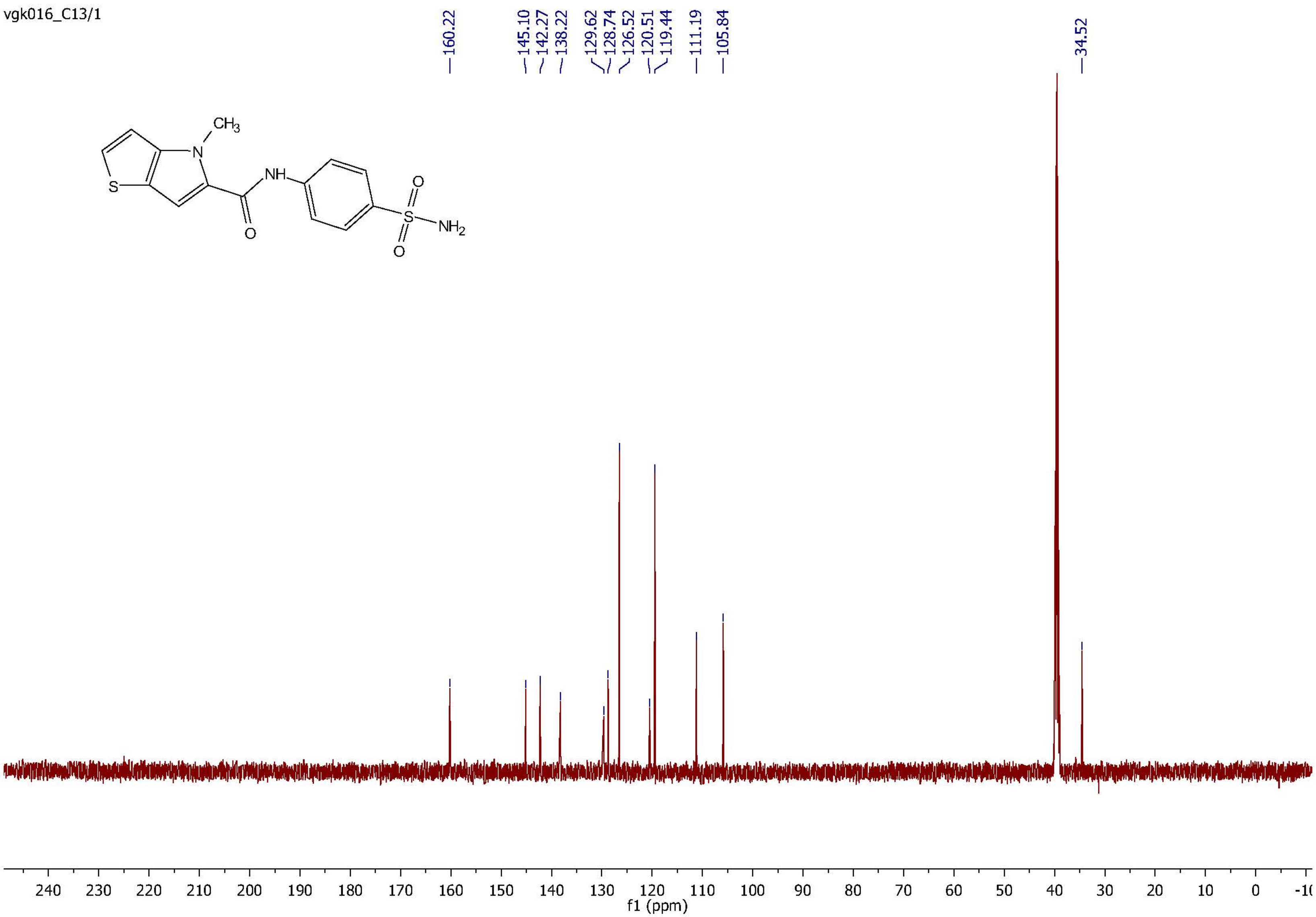

Figure S27. ^{13}C NMR spectrum of *N-[4-(Aminosulfonyl)phenyl]-4-methyl-4H-thieno[3,2-b]pyrrole-5-carboxamide* (**8a**).

MaxPeak: 100.00%
Ret_Time: 1.091 min

Mol Wt 0
Exact Mass

#	Time	Area%
1	1.091	100.00

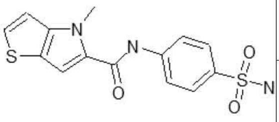

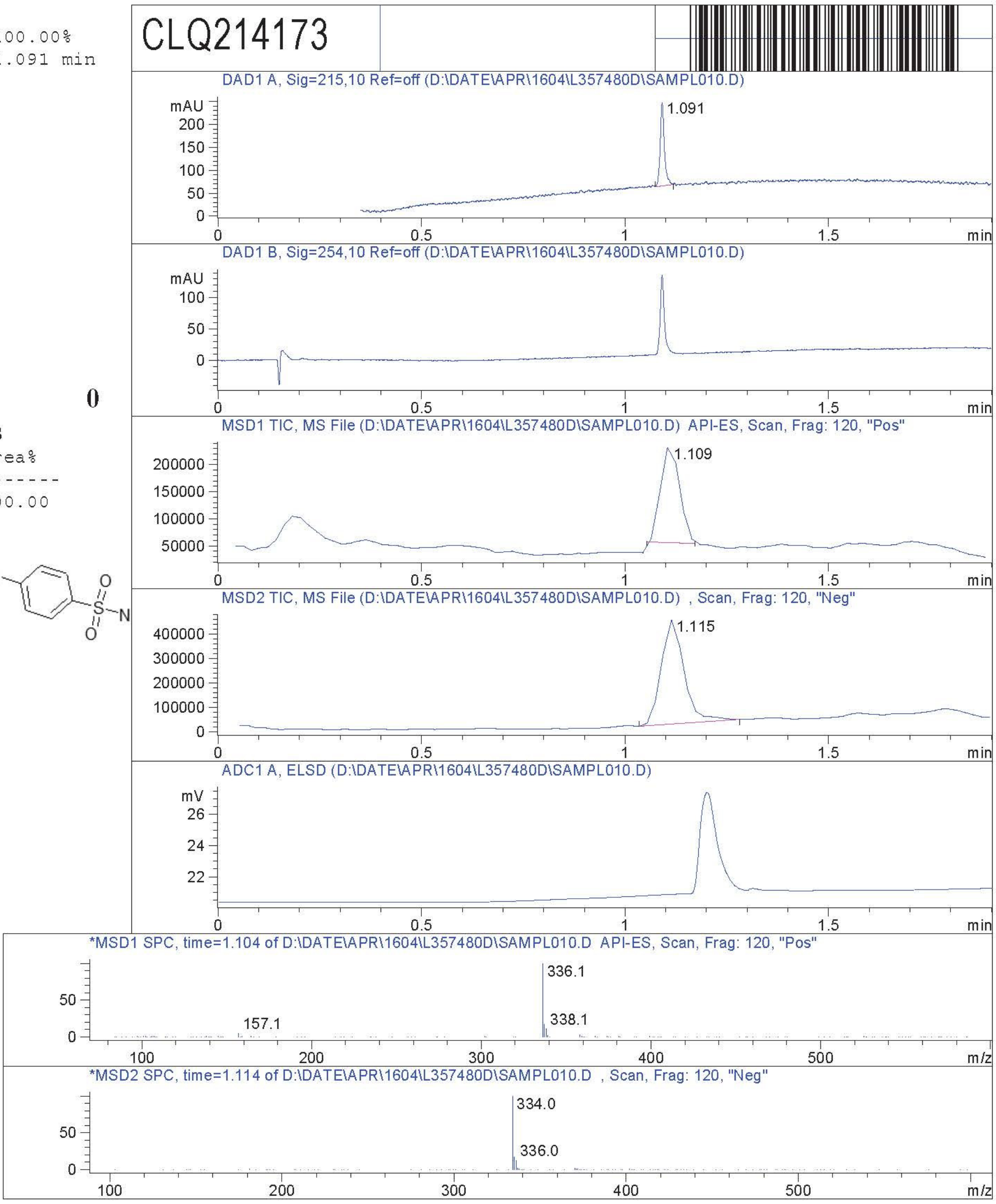

RT 1.109

RT 1.115

Inj.Date 4/15/2021 Y -SL- Acq. Method C:\HPCHEM\-> ->

Figure S28. LCMS spectrum of *N-[4-(Aminosulfonyl)phenyl]-4-methyl-4H-thieno[3,2-b]pyrrole-5-carboxamide* (**8a**).

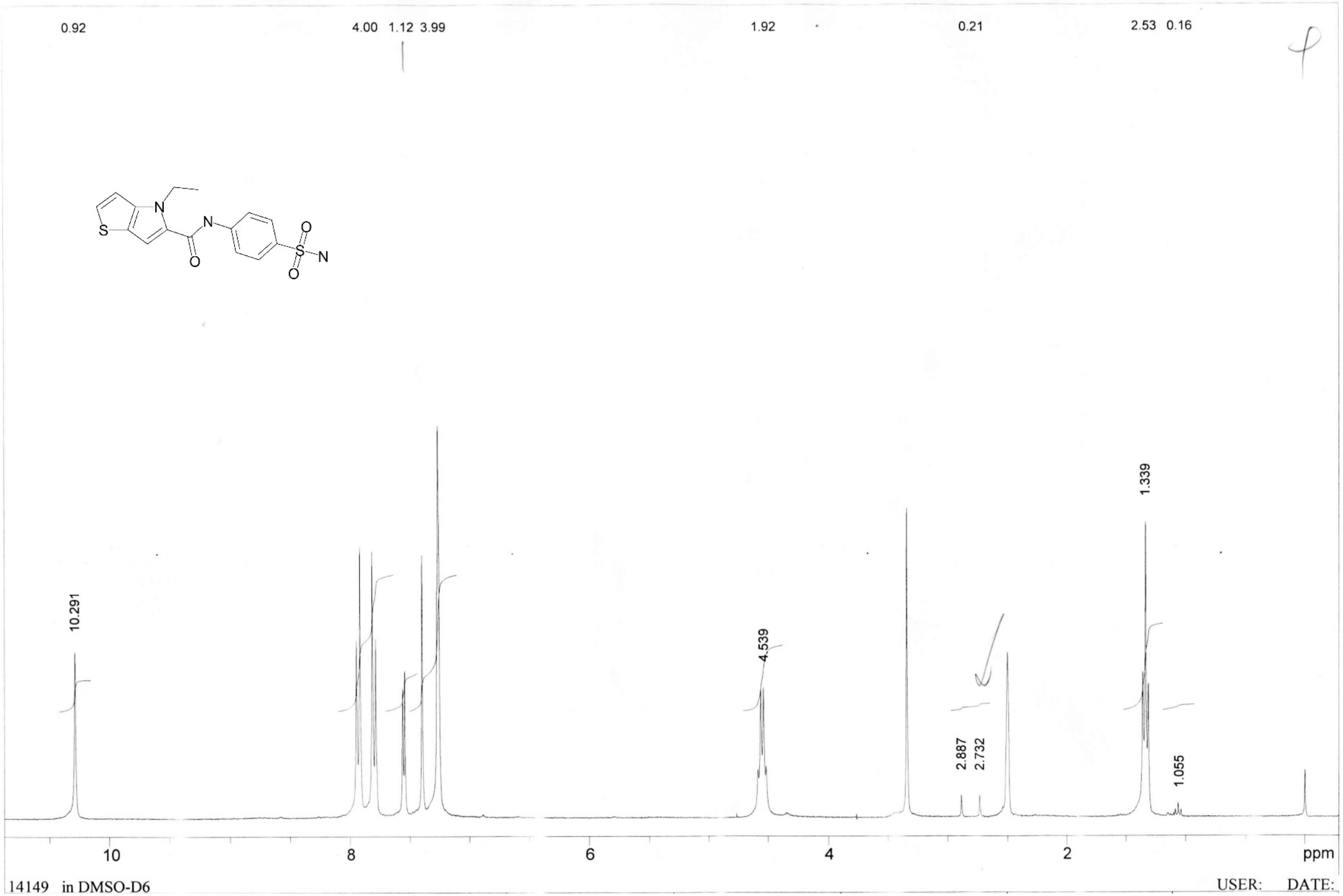

Figure S29. ^{1}H NMR spectrum of *N-[4-(Aminosulfonyl)phenyl]-4-ethyl-4H-thieno[3,2-b]pyrrole-5-carboxamide* (**8b**).

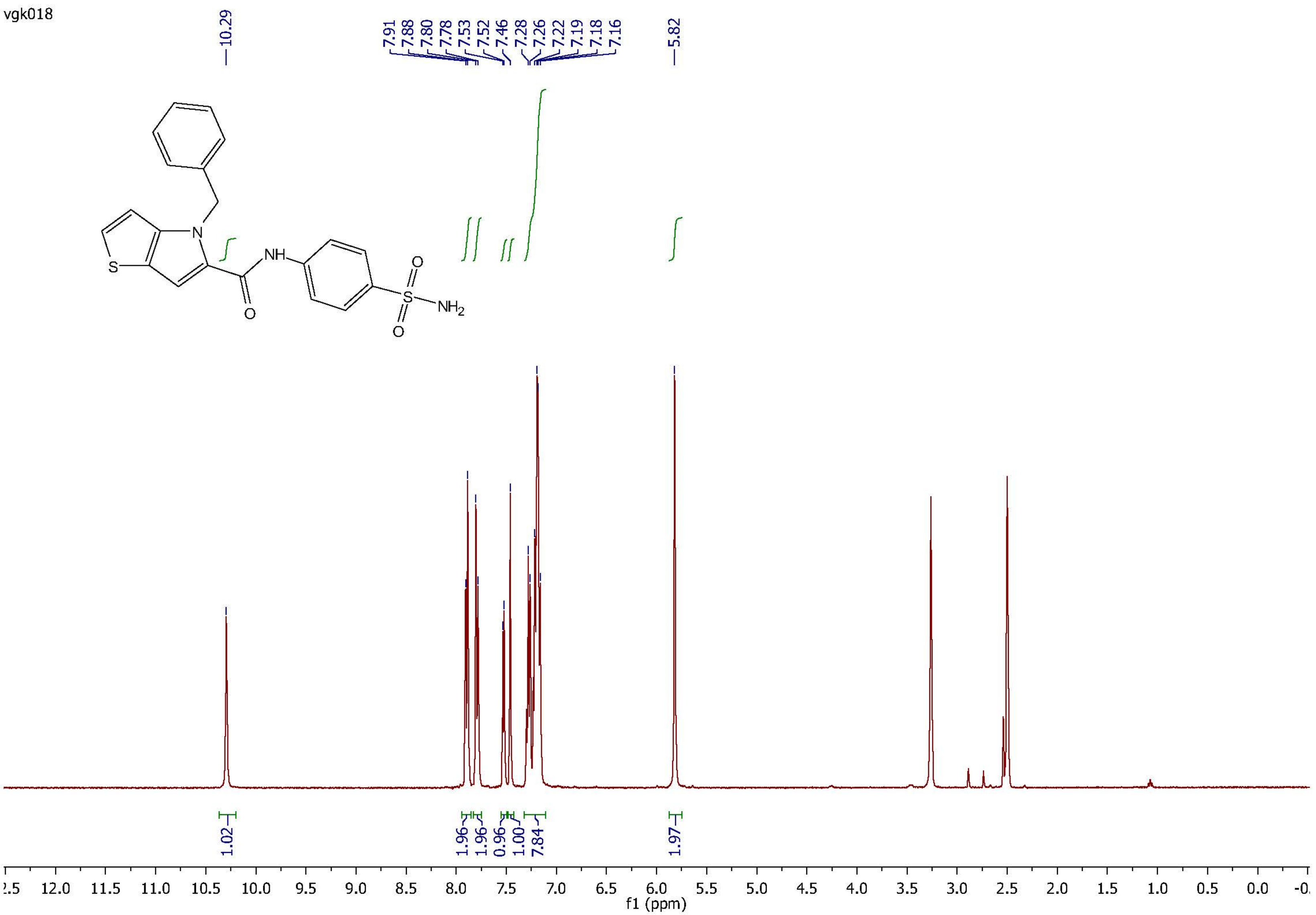

Figure S30. ^{1}H NMR spectrum of *N-[4-(Aminosulfonyl)phenyl]-4-benzyl-4H-thieno[3,2-b]pyrrole-5-carboxamide* (**8c**).

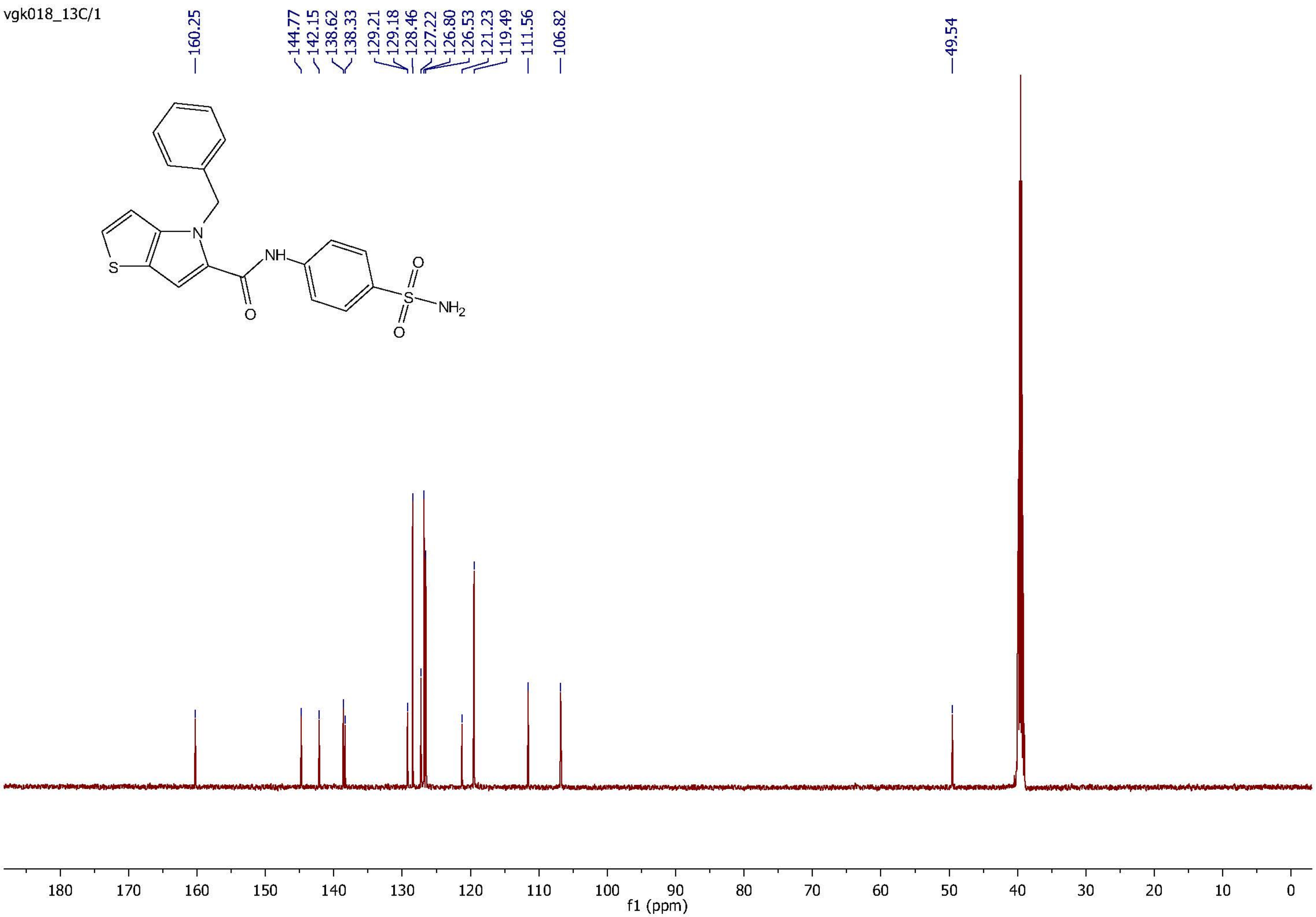

Figure S31. ^{13}C NMR spectrum of *N-[4-(Aminosulfonyl)phenyl]-4-benzyl-4H-thieno[3,2-b]pyrrole-5-carboxamide* (**8c**).

MaxPeak: 100.00%
Ret_Time: 1.339 min

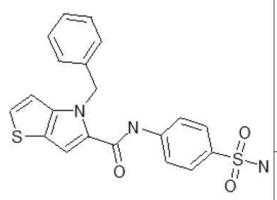

Mol Wt 0

Exact Mass

#	Time	Area%
1	1.339	100.00

RT 1.347

RT 1.347

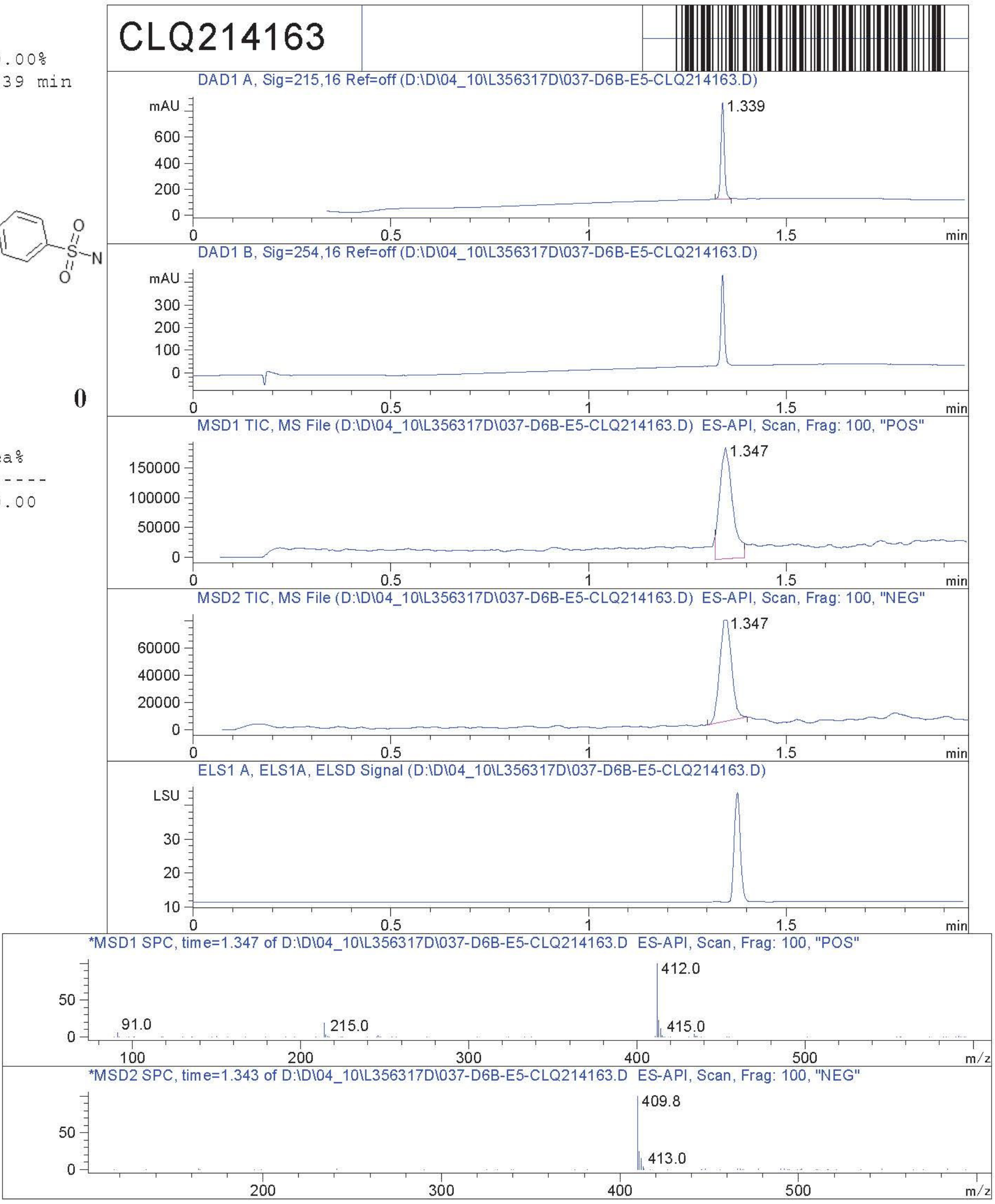

Inj.Date 4/9/2021 N Acq. Method C:\Chem32\-> ->

Figure S32. LCMS spectrum of *N-[4-(Aminosulfonyl)phenyl]-4-benzyl-4H-thieno[3,2-b]pyrrole-5-carboxamide* (**8c**).

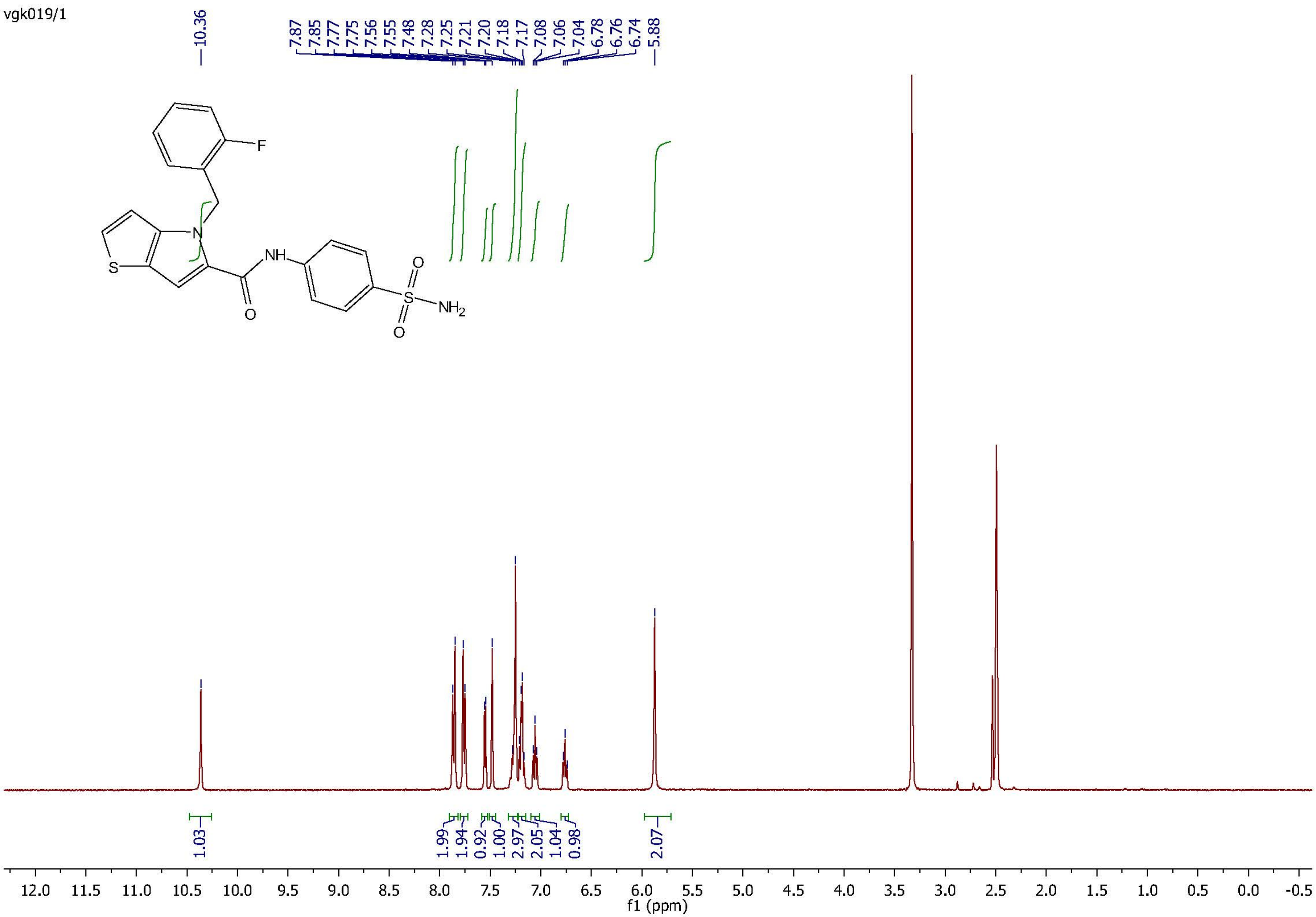

Figure S33. ^{1}H NMR spectrum of *N-[4-(Aminosulfonyl)phenyl]-4-(2-fluorobenzyl)-4H-thieno[3,2-b]pyrrole-5-carboxamide* (**8d**).

MaxPeak: 100.00%
Ret_Time: 1.294 min

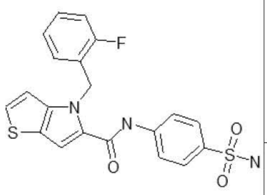

Mol Wt 0
Exact Mass

#	Time	Area%
1	1.294	100.00

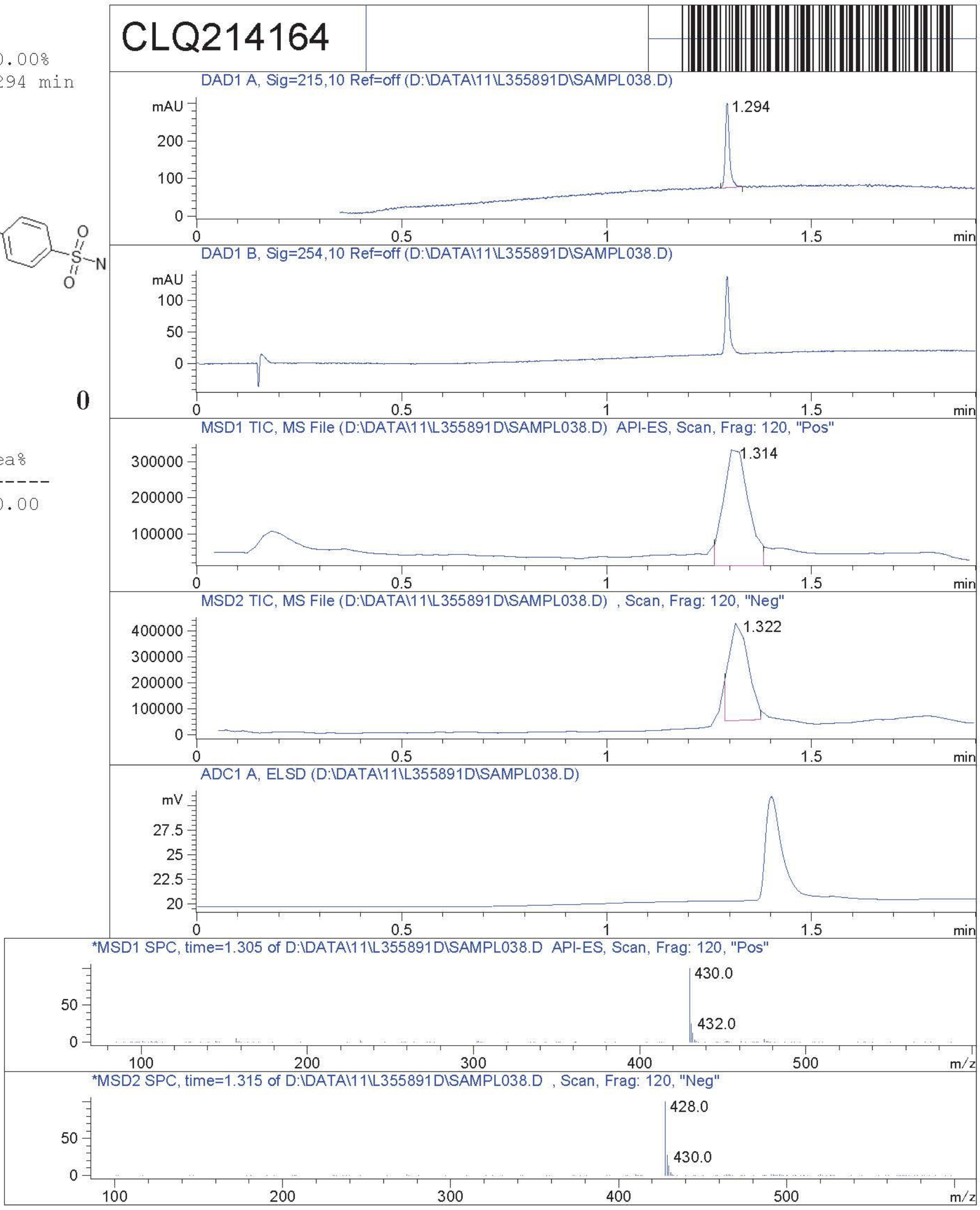

RT 1.314

RT 1.322

Inj.Date 4/10/2021 LB -SL- Acq. Method C:\HPCHEM\-> ->

Figure S34. LCMS spectrum of *N-[4-(Aminosulfonyl)phenyl]-4-(2-fluorobenzyl)-4H-thieno[3,2-b]pyrrole-5-carboxamide* (**8d**).

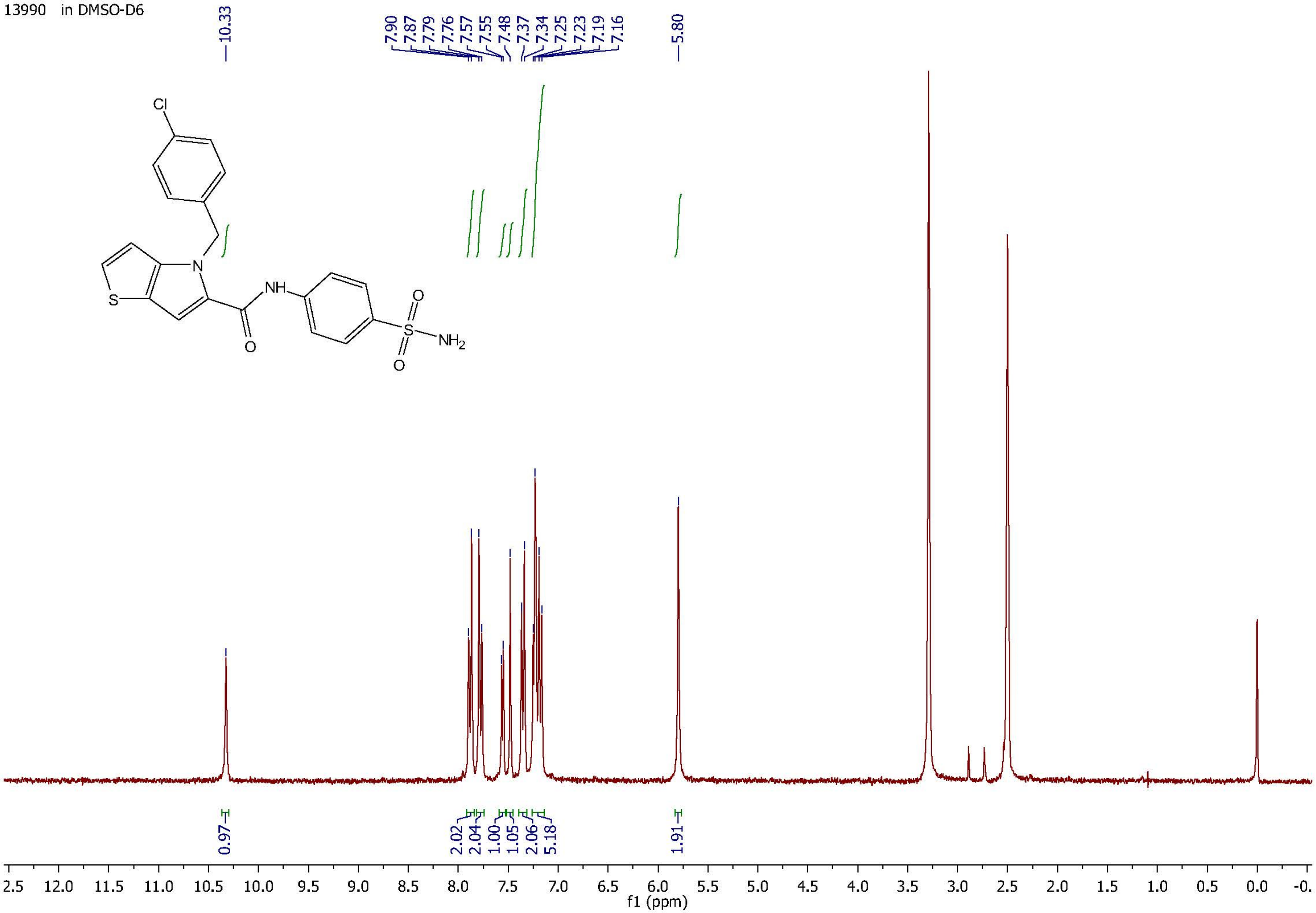

Figure S35. ^{1}H NMR spectrum of *N-[4-(Aminosulfonyl)phenyl]-4-(4-chlorobenzyl)-4H-thieno[3,2-b]pyrrole-5-carboxamide* (**8f**).

MaxPeak: 100.00%
Ret_Time: 1.406 min

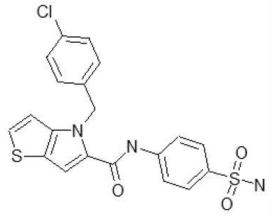

Mol Wt 0
Exact Mass

#	Time	Area%
1	1.406	100.00

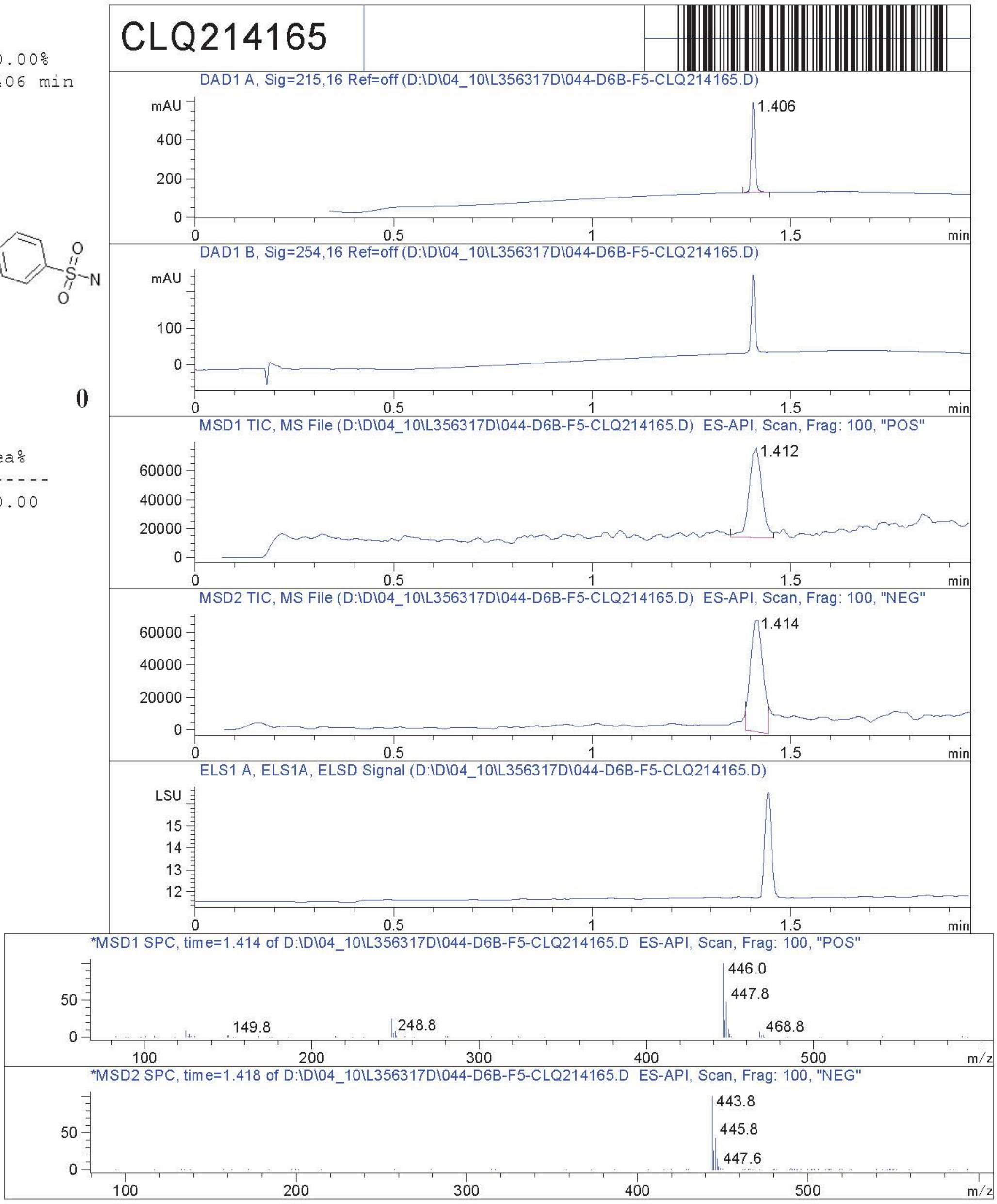

RT 1.412

RT 1.414

Inj.Date 4/9/2021 N -15- Acq. Method C:\Chem32\-> ->

Figure S36. LCMS spectrum of *N-[4-(Aminosulfonyl)phenyl]-4-(4-chlorobenzyl)-4H-thieno[3,2-b]pyrrole-5-carboxamide* (**8f**).

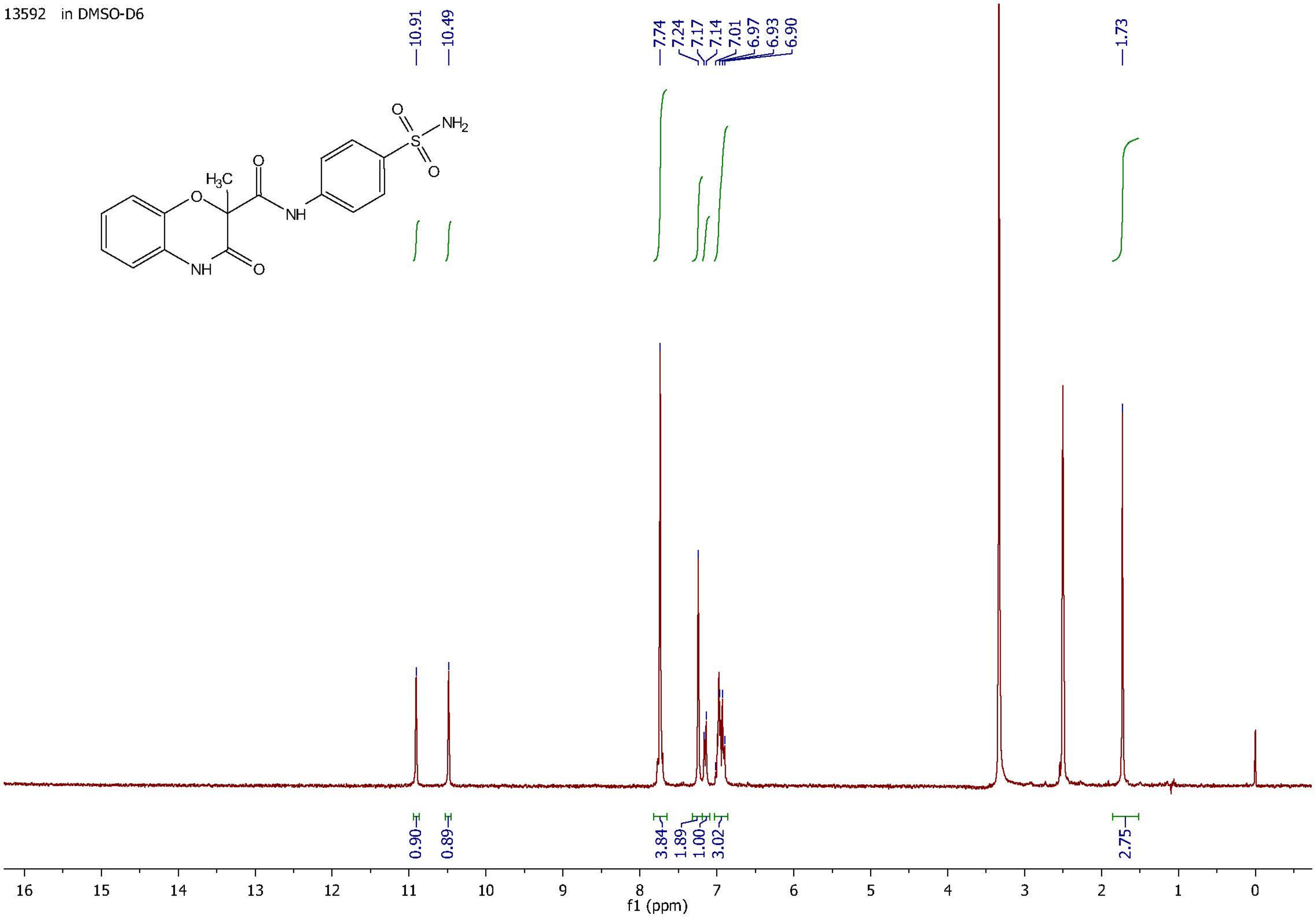

Figure S41. ^{1}H NMR spectrum of *N-[4-(Aminosulfonyl)phenyl]-2-methyl-3-oxo-3,4-dihydro-2H-1,4-benzoxazine-2-carboxamide* (**11**).

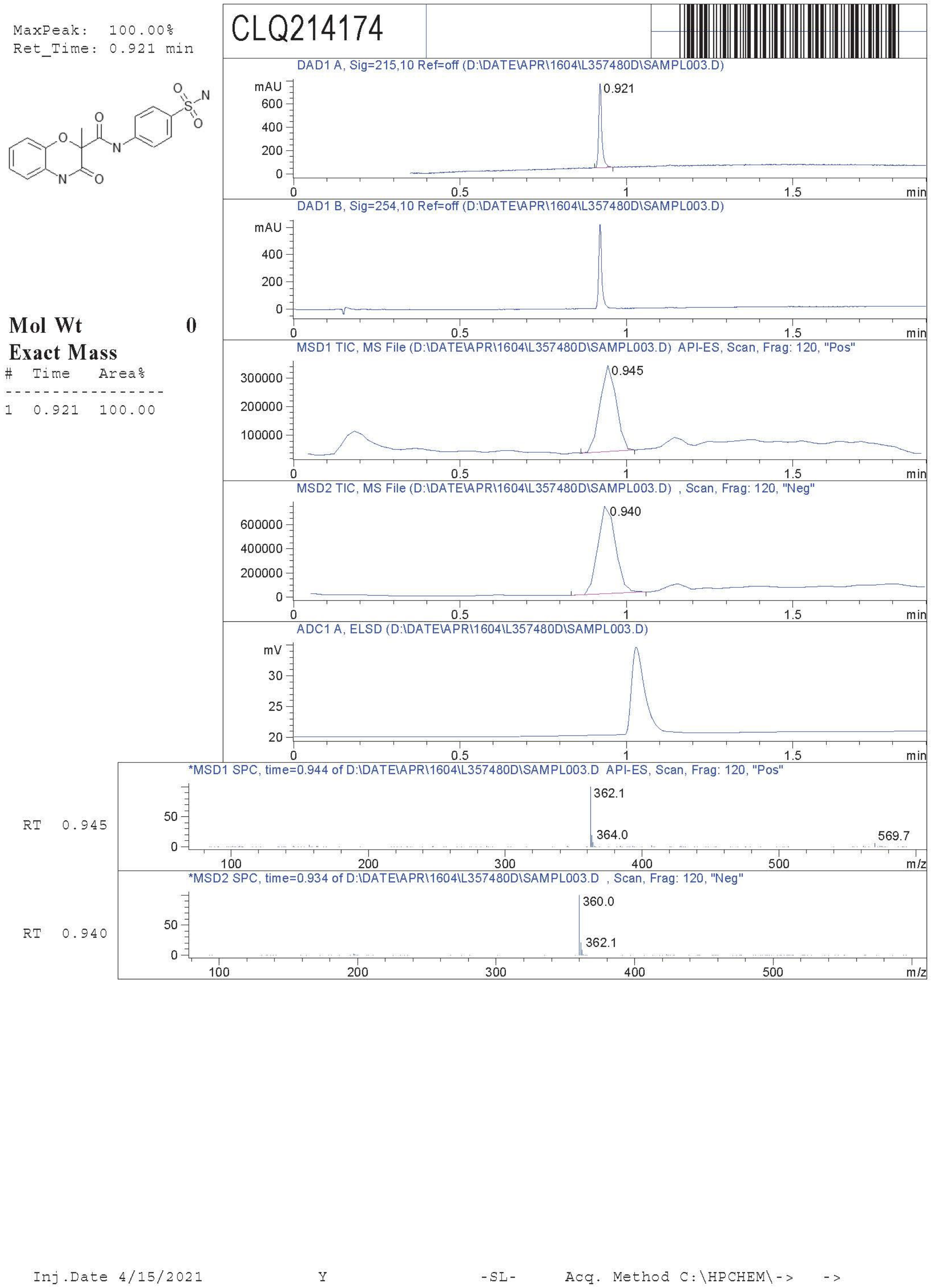

Figure S42. LCMS spectrum of *N-[4-(Aminosulfonyl)phenyl]-2-methyl-3-oxo-3,4-dihydro-2H-1,4-benzoxazine-2-carboxamide* (**11**).

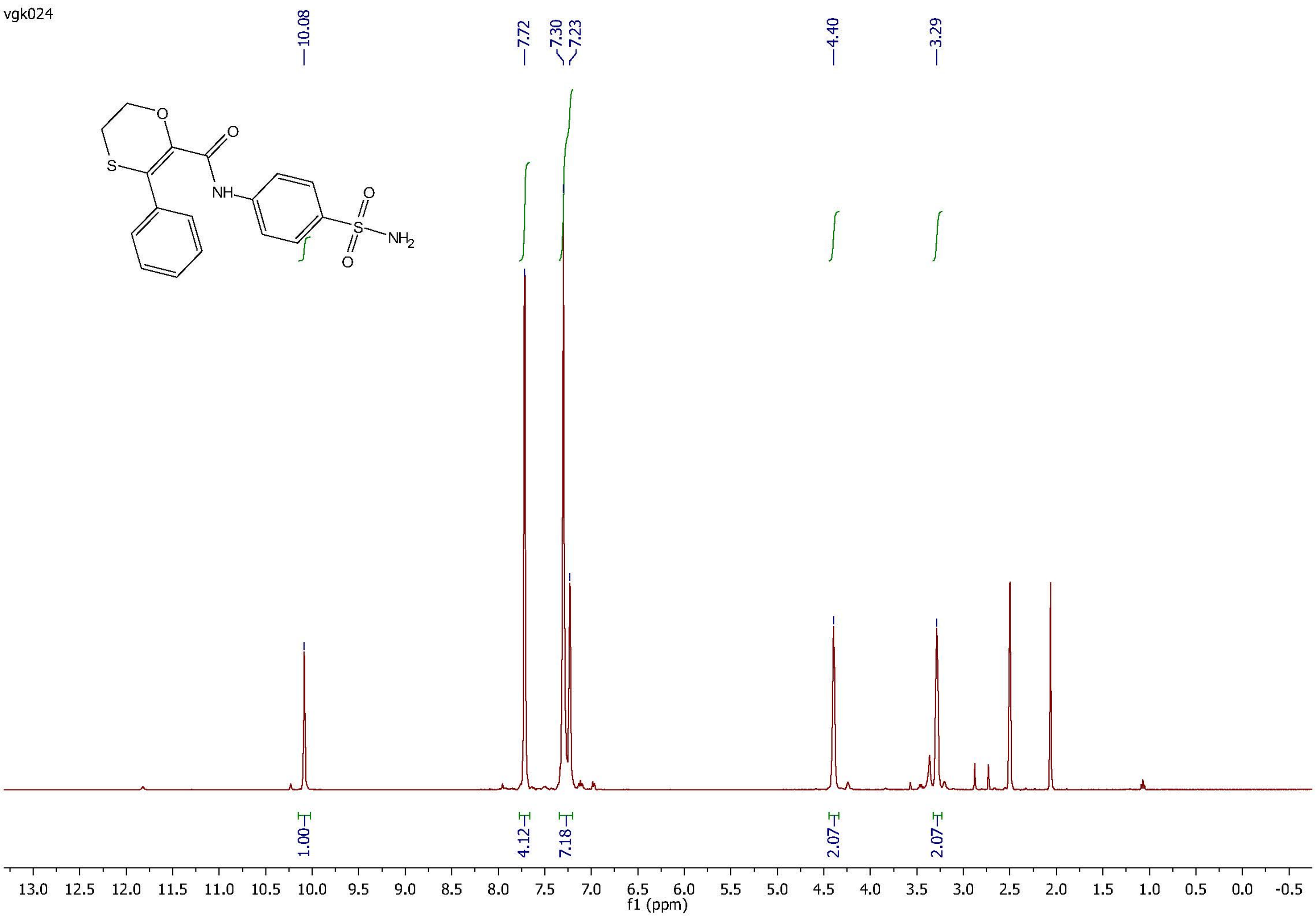

Figure S43. ^{1}H NMR spectrum of *N-[4-(Aminosulfonyl)phenyl]-3-phenyl-5,6-dihydro-1,4-oxathiine-2-carboxamide* (**14a**).

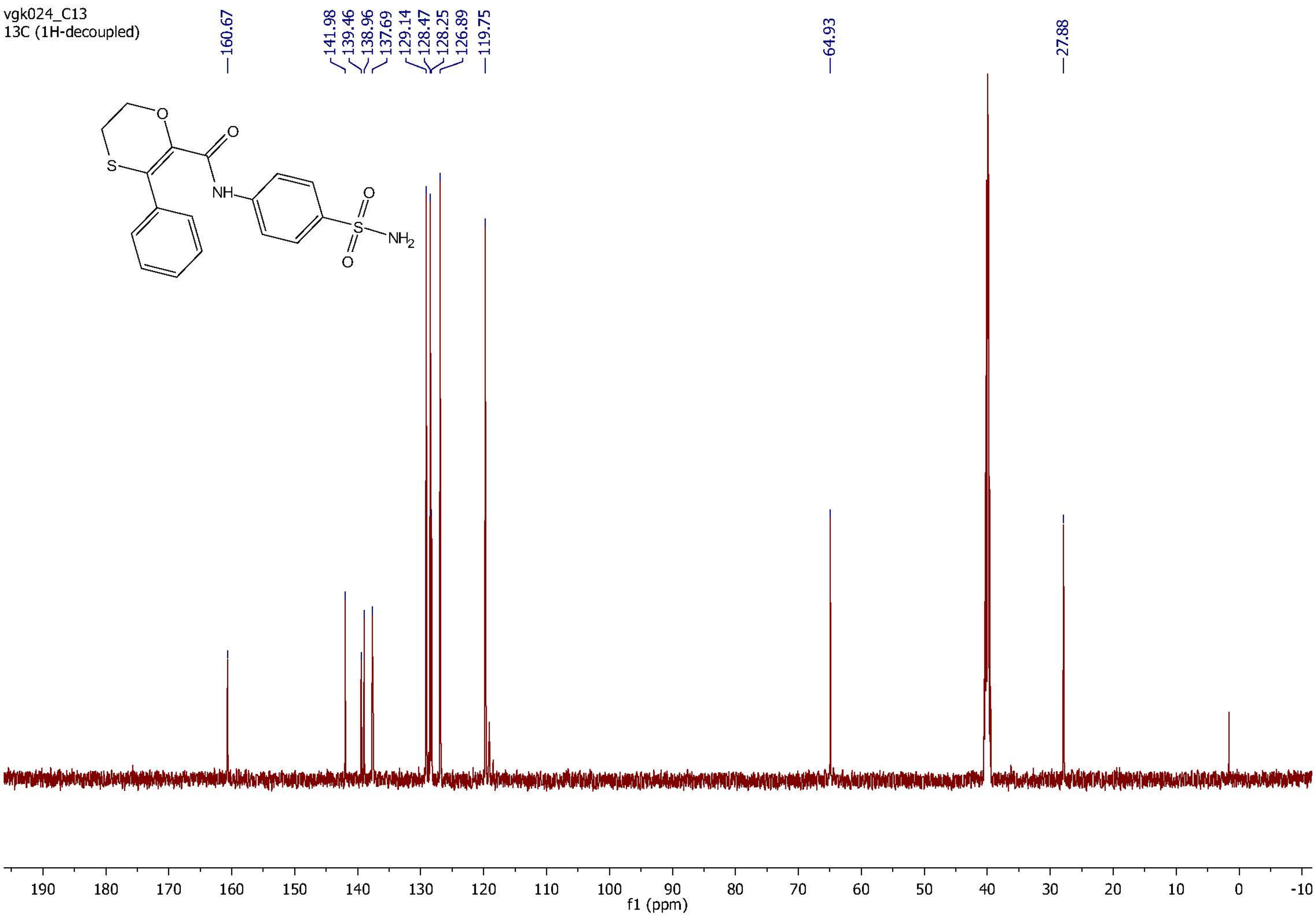

Figure S44. ^{13}C NMR spectrum of *N-[4-(Aminosulfonyl)phenyl]-3-phenyl-5,6-dihydro-1,4-oxathiine-2-carboxamide* (**14a**).

MaxPeak: 93.92%
Ret_Time: 1.120 min

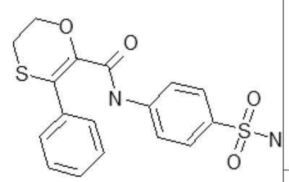

Mol Wt 0
Exact Mass

#	Time	Area%
1	1.120	93.92
2	1.413	6.08

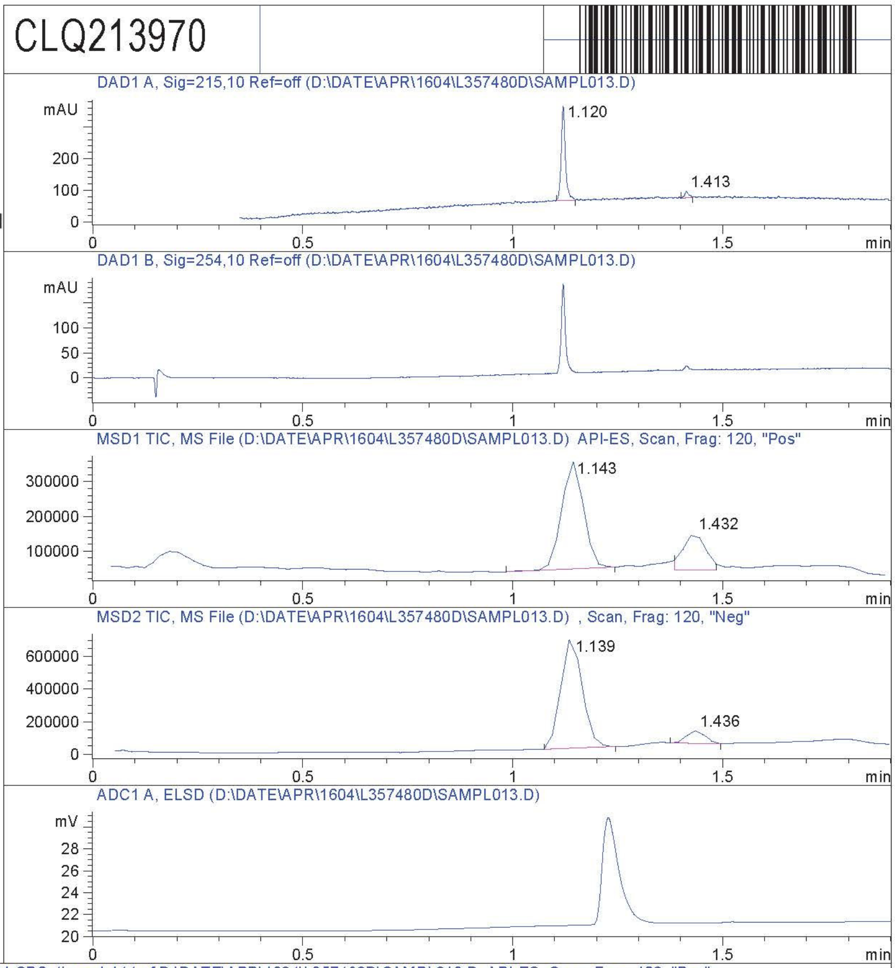

RT 1.143

RT 1.432

RT 1.139

RT 1.436

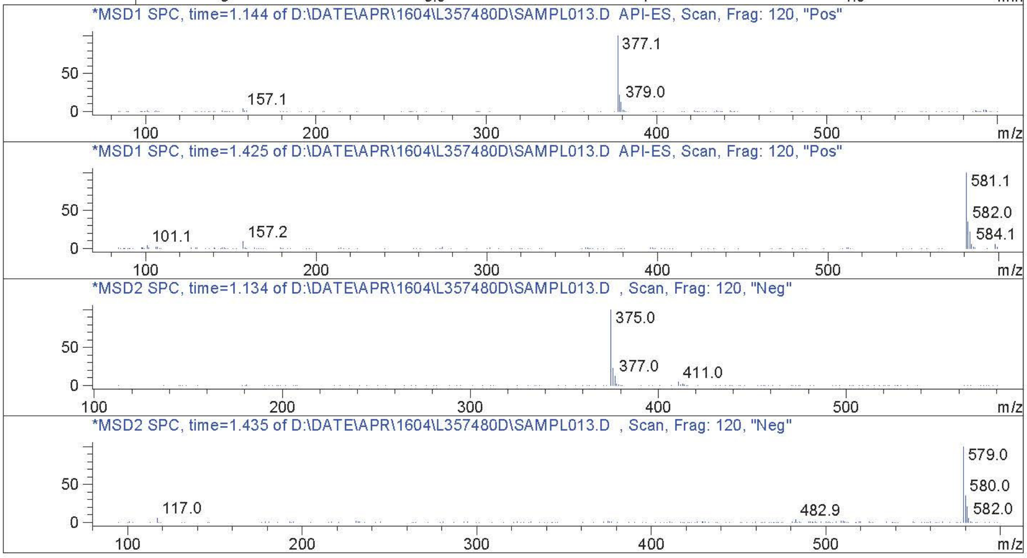

Inj.Date 4/15/2021 Y -SL- Acq. Method C:\HPCHEM\-> ->

Figure S45. LCMS spectrum of *N-[4-(Aminosulfonyl)phenyl]-3-phenyl-5,6-dihydro-1,4-oxathiine-2-carboxamide* (**14a**).

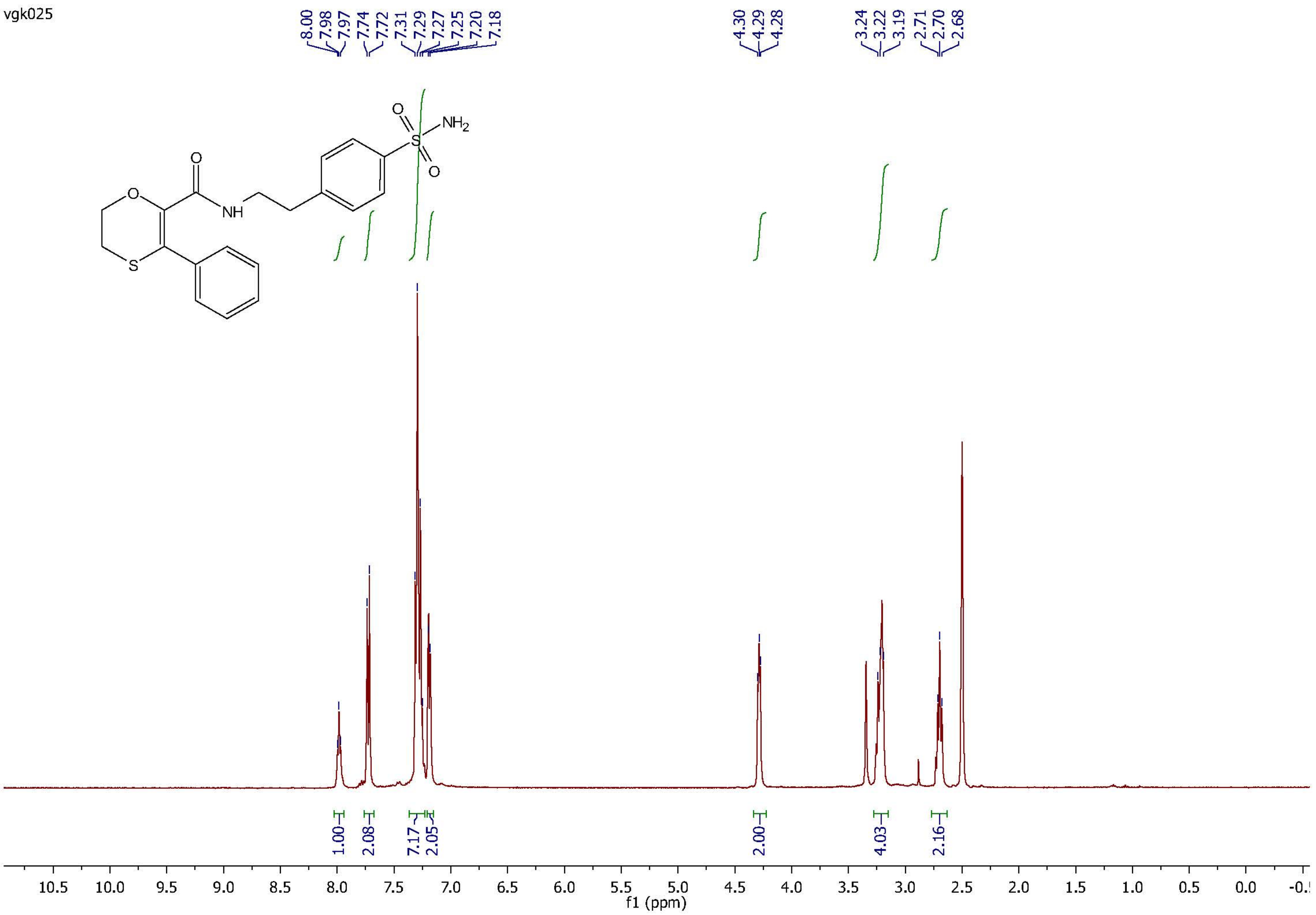

Figure S46. ^{1}H NMR spectrum of *N-{2-[4-(Aminosulfonyl)phenyl]ethyl}-3-phenyl-5,6-dihydro-1,4-oxathiine-2-carboxamide* (**14b**).

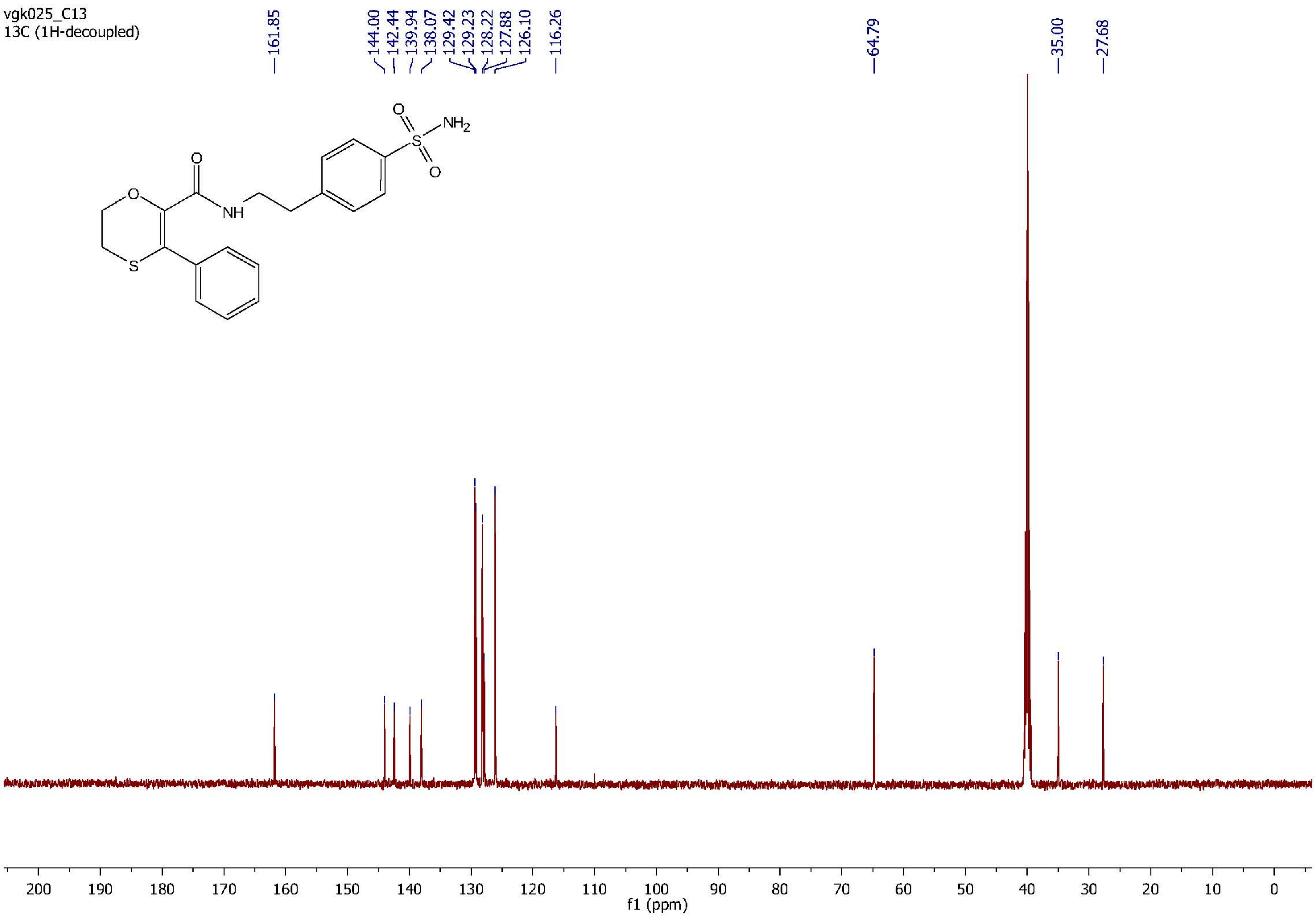

Figure S47. ^{13}C NMR spectrum of *N-{2-[4-(Aminosulfonyl)phenyl]ethyl}-3-phenyl-5,6-dihydro-1,4-oxathiine-2-carboxamide* (**14b**).

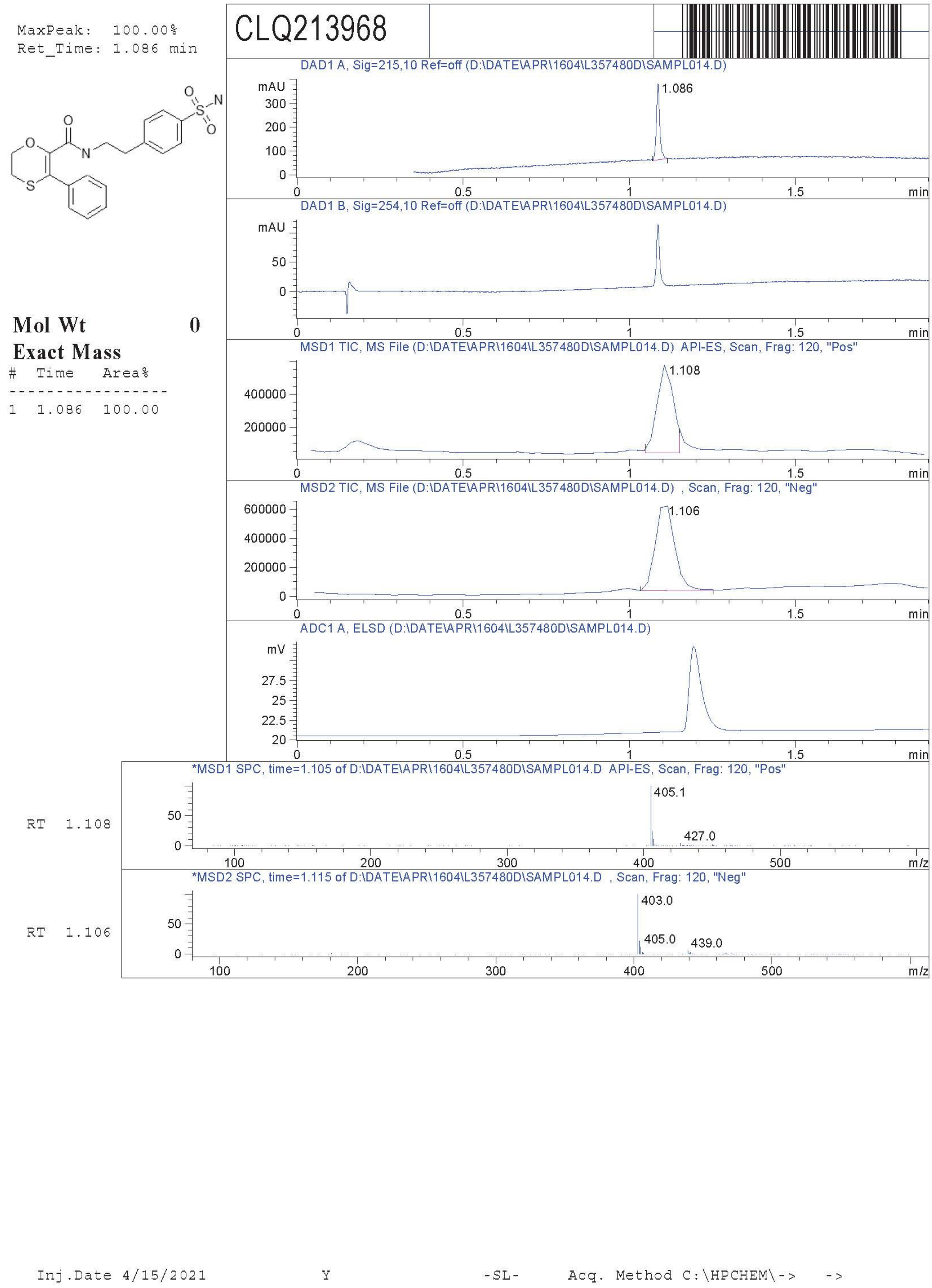

Figure S48. LCMS spectrum of *N-{2-[4-(Aminosulfonyl)phenyl]ethyl}-3-phenyl-5,6-dihydro-1,4-oxathiine-2-carboxamide* (**14b**).
